# Supplementary material for: Niche-processes induced differences in plant growth, carbon balance, stress resistance, and regeneration affect community assembly over succession
Source: PLoS One. 2020 Feb 28;15(2):e0229443. doi: 10.1371/journal.pone.0229443 (PMC7048279; doi:10.1371/journal.pone.0229443)
Supplement: S1 Table — The values for fitted ANCOVA lines (FAL) are also shown. (DOC) [file pone.0229443.s001.doc]

### **Table S1.** The long-transformed values of abundance (Abundance) and mean value of traits (Value) for each species found in each successional age in chronosequence 1 and 2, including specific leaf area (SLA; g cm^-2^), seed mass (SM; g), seed germination rate (SG;%), plant height (H, cm), photosynthesis rate (A; µmol m^-2^ s^-1^) and leaf proline content (Pro; mg/kg). The values for fitted ANCOVA lines (FAL) are also shown.

| Value | Abundance | FAL | Age | | Chronosequence | Trait |
| --- | --- | --- | --- | --- | --- | --- |
| 2.36 | 2.40 | 1.67 | 4 | | 1 | SLA |
| 2.44 | 0.85 | 1.40 | 4 | | 1 | SLA |
| 2.49 | 0.00 | 1.17 | 4 | | 1 | SLA |
| 2.58 | 1.65 | 0.84 | 4 | | 1 | SLA |
| 2.60 | 0.00 | 0.76 | 4 | | 1 | SLA |
| 2.30 | 2.84 | 1.91 | 4 | | 1 | SLA |
| 2.57 | 0.30 | 0.87 | 4 | | 1 | SLA |
| 2.50 | 1.69 | 1.15 | 4 | | 1 | SLA |
| 2.35 | 0.78 | 1.72 | 4 | | 1 | SLA |
| 2.50 | 0.30 | 1.13 | 4 | | 1 | SLA |
| 2.27 | 1.79 | 2.04 | 4 | | 1 | SLA |
| 2.43 | 1.79 | 1.41 | 4 | | 1 | SLA |
| 2.55 | 0.00 | 0.97 | 4 | | 1 | SLA |
| 2.04 | 2.39 | 2.92 | 4 | | 1 | SLA |
| 2.57 | 2.10 | 0.86 | 4 | | 1 | SLA |
| 2.56 | 1.90 | 0.90 | 4 | | 1 | SLA |
| 2.49 | 0.48 | 1.19 | 4 | | 1 | SLA |
| 2.54 | 0.00 | 0.98 | 4 | | 1 | SLA |
| 2.52 | 1.26 | 1.08 | 4 | | 1 | SLA |
| 2.57 | 1.18 | 0.86 | 4 | | 1 | SLA |
| 2.48 | 2.29 | 1.24 | 4 | | 1 | SLA |
| 2.54 | 0.70 | 0.98 | 4 | | 1 | SLA |
| 2.59 | 1.08 | 0.81 | 4 | | 1 | SLA |
| 2.54 | 0.30 | 0.98 | 4 | | 1 | SLA |
| 2.08 | 2.89 | 2.75 | 4 | | 1 | SLA |
| 2.38 | 2.16 | 1.59 | 4 | | 1 | SLA |
| 2.52 | 1.18 | 1.07 | 4 | | 1 | SLA |
| 2.44 | 1.89 | 1.37 | 4 | | 1 | SLA |
| 2.50 | 1.69 | 1.14 | 4 | | 1 | SLA |
| 2.38 | 2.56 | 1.62 | 4 | | 1 | SLA |
| 2.30 | 1.99 | 1.91 | 4 | | 1 | SLA |
| 2.42 | 0.30 | 1.47 | 4 | | 1 | SLA |
| 2.44 | 2.22 | 1.48 | 6 | | 1 | SLA |
| 2.20 | 2.18 | 2.05 | 6 | | 1 | SLA |
| 2.25 | 2.05 | 1.92 | 6 | | 1 | SLA |
| 2.13 | 2.62 | 2.19 | 6 | | 1 | SLA |
| 2.31 | 0.90 | 1.78 | 6 | | 1 | SLA |
| 2.62 | 1.00 | 1.09 | 6 | | 1 | SLA |
| 2.35 | 0.60 | 1.69 | 6 | | 1 | SLA |
| 2.48 | 0.48 | 1.39 | 6 | | 1 | SLA |
| 2.62 | 0.30 | 1.08 | 6 | | 1 | SLA |
| 2.64 | 0.30 | 1.04 | 6 | | 1 | SLA |
| 2.48 | 1.88 | 1.39 | 6 | | 1 | SLA |
| 2.39 | 1.84 | 1.60 | 6 | | 1 | SLA |
| 2.54 | 0.70 | 1.27 | 6 | | 1 | SLA |
| 2.42 | 1.68 | 1.53 | 6 | | 1 | SLA |
| 2.24 | 1.40 | 1.96 | 6 | | 1 | SLA |
| 2.52 | 1.04 | 1.30 | 6 | | 1 | SLA |
| 2.49 | 2.26 | 1.38 | 6 | | 1 | SLA |
| 2.38 | 3.00 | 1.62 | 6 | | 1 | SLA |
| 2.16 | 3.01 | 2.12 | 6 | | 1 | SLA |
| 2.60 | 1.95 | 1.14 | 6 | | 1 | SLA |
| 2.42 | 1.99 | 1.54 | 6 | | 1 | SLA |
| 2.59 | 2.02 | 1.15 | 6 | | 1 | SLA |
| 2.46 | 1.08 | 1.44 | 6 | | 1 | SLA |
| 2.51 | 1.89 | 1.34 | 6 | | 1 | SLA |
| 2.27 | 1.20 | 1.87 | 6 | | 1 | SLA |
| 2.30 | 1.00 | 1.82 | 6 | | 1 | SLA |
| 2.47 | 1.18 | 1.43 | 6 | | 1 | SLA |
| 2.44 | 1.38 | 1.50 | 6 | | 1 | SLA |
| 2.46 | 1.71 | 1.44 | 6 | | 1 | SLA |
| 2.34 | 1.53 | 1.71 | 6 | | 1 | SLA |
| 2.56 | 0.70 | 1.22 | 6 | | 1 | SLA |
| 2.64 | 1.59 | 1.03 | 6 | | 1 | SLA |
| 2.53 | 1.11 | 1.29 | 6 | | 1 | SLA |
| 2.36 | 0.90 | 1.55 | 10 | | 1 | SLA |
| 2.38 | 1.66 | 1.56 | 10 | | 1 | SLA |
| 2.37 | 1.48 | 1.56 | 10 | | 1 | SLA |
| 2.40 | 1.52 | 1.58 | 10 | | 1 | SLA |
| 2.27 | 1.20 | 1.50 | 10 | | 1 | SLA |
| 2.31 | 0.00 | 1.53 | 10 | | 1 | SLA |
| 2.45 | 1.80 | 1.61 | 10 | | 1 | SLA |
| 2.32 | 2.16 | 1.53 | 10 | | 1 | SLA |
| 2.28 | 2.50 | 1.51 | 10 | | 1 | SLA |
| 2.30 | 0.00 | 1.52 | 10 | | 1 | SLA |
| 2.39 | 1.68 | 1.57 | 10 | | 1 | SLA |
| 2.33 | 0.60 | 1.54 | 10 | | 1 | SLA |
| 2.55 | 0.00 | 1.67 | 10 | | 1 | SLA |
| 2.41 | 1.43 | 1.59 | 10 | | 1 | SLA |
| 2.42 | 2.05 | 1.59 | 10 | | 1 | SLA |
| 2.41 | 1.94 | 1.58 | 10 | | 1 | SLA |
| 2.38 | 1.58 | 1.56 | 10 | | 1 | SLA |
| 2.43 | 1.20 | 1.59 | 10 | | 1 | SLA |
| 2.25 | 2.34 | 1.49 | 10 | | 1 | SLA |
| 2.43 | 1.81 | 1.59 | 10 | | 1 | SLA |
| 2.58 | 1.65 | 1.69 | 10 | | 1 | SLA |
| 2.22 | 2.96 | 1.47 | 10 | | 1 | SLA |
| 2.51 | 2.47 | 1.64 | 10 | | 1 | SLA |
| 2.50 | 1.04 | 1.64 | 10 | | 1 | SLA |
| 2.28 | 1.98 | 1.51 | 10 | | 1 | SLA |
| 2.57 | 2.11 | 1.68 | 10 | | 1 | SLA |
| 2.45 | 2.11 | 1.61 | 10 | | 1 | SLA |
| 2.33 | 2.50 | 1.53 | 10 | | 1 | SLA |
| 2.40 | 1.32 | 1.58 | 10 | | 1 | SLA |
| 2.42 | 1.36 | 1.59 | 10 | | 1 | SLA |
| 2.10 | 1.87 | 1.40 | 10 | | 1 | SLA |
| 2.23 | 0.48 | 1.47 | 10 | | 1 | SLA |
| 2.59 | 3.16 | 1.69 | 10 | | 1 | SLA |
| 2.29 | 1.26 | 1.51 | 10 | | 1 | SLA |
| 2.58 | 2.22 | 1.68 | 10 | | 1 | SLA |
| 2.37 | 0.00 | 1.56 | 10 | | 1 | SLA |
| 2.34 | 2.52 | 1.54 | 10 | | 1 | SLA |
| 2.18 | 1.00 | 1.44 | 10 | | 1 | SLA |
| 2.61 | 1.15 | 1.70 | 10 | | 1 | SLA |
| 2.32 | 2.89 | 1.53 | 10 | | 1 | SLA |
| 2.40 | 1.30 | 1.58 | 10 | | 1 | SLA |
| 2.24 | 1.84 | 1.48 | 10 | | 1 | SLA |
| 2.45 | 1.26 | 1.61 | 10 | | 1 | SLA |
| 2.46 | 1.60 | 1.62 | 10 | | 1 | SLA |
| 2.54 | 2.47 | 1.66 | 10 | | 1 | SLA |
| 2.42 | 0.30 | 1.59 | 10 | | 1 | SLA |
| 2.01 | 1.04 | 1.35 | 10 | | 1 | SLA |
| 2.56 | 1.52 | 1.67 | 10 | | 1 | SLA |
| 2.23 | 2.56 | 1.38 | 13 | | 1 | SLA |
| 2.43 | 1.23 | 1.72 | 13 | | 1 | SLA |
| 2.22 | 1.94 | 1.36 | 13 | | 1 | SLA |
| 2.63 | 2.81 | 2.05 | 13 | | 1 | SLA |
| 2.46 | 2.35 | 1.77 | 13 | | 1 | SLA |
| 2.49 | 0.90 | 1.82 | 13 | | 1 | SLA |
| 2.48 | 1.52 | 1.79 | 13 | | 1 | SLA |
| 2.38 | 2.08 | 1.63 | 13 | | 1 | SLA |
| 2.53 | 1.20 | 1.87 | 13 | | 1 | SLA |
| 2.54 | 1.54 | 1.90 | 13 | | 1 | SLA |
| 2.58 | 2.42 | 1.97 | 13 | | 1 | SLA |
| 2.47 | 2.30 | 1.78 | 13 | | 1 | SLA |
| 2.45 | 2.78 | 1.75 | 13 | | 1 | SLA |
| 2.61 | 2.41 | 2.01 | 13 | | 1 | SLA |
| 2.41 | 2.38 | 1.69 | 13 | | 1 | SLA |
| 2.42 | 1.98 | 1.69 | 13 | | 1 | SLA |
| 2.57 | 0.30 | 1.95 | 13 | | 1 | SLA |
| 2.56 | 2.64 | 1.93 | 13 | | 1 | SLA |
| 2.32 | 1.72 | 1.53 | 13 | | 1 | SLA |
| 2.34 | 2.47 | 1.56 | 13 | | 1 | SLA |
| 2.53 | 2.09 | 1.89 | 13 | | 1 | SLA |
| 2.51 | 0.48 | 1.85 | 13 | | 1 | SLA |
| 2.36 | 2.55 | 1.60 | 13 | | 1 | SLA |
| 2.27 | 0.00 | 1.45 | 13 | | 1 | SLA |
| 2.47 | 1.00 | 1.78 | 13 | | 1 | SLA |
| 2.52 | 1.70 | 1.86 | 13 | | 1 | SLA |
| 2.49 | 1.85 | 1.82 | 13 | | 1 | SLA |
| 2.56 | 2.95 | 1.92 | 13 | | 1 | SLA |
| 2.51 | 1.30 | 1.85 | 13 | | 1 | SLA |
| 2.58 | 1.74 | 1.97 | 13 | | 1 | SLA |
| 2.61 | 1.93 | 2.01 | 13 | | 1 | SLA |
| 2.33 | 1.30 | 1.54 | 13 | | 1 | SLA |
| 2.54 | 1.48 | 1.90 | 13 | | 1 | SLA |
| 2.39 | 1.48 | 1.64 | 13 | | 1 | SLA |
| 2.07 | 0.60 | 1.11 | 13 | | 1 | SLA |
| 2.08 | 0.48 | 1.13 | 13 | | 1 | SLA |
| 2.39 | 2.78 | 1.73 | Undisturbed | | 1 | SLA |
| 2.38 | 2.12 | 1.70 | Undisturbed | | 1 | SLA |
| 2.23 | 1.67 | 1.34 | Undisturbed | | 1 | SLA |
| 2.43 | 2.49 | 1.82 | Undisturbed | | 1 | SLA |
| 2.33 | 1.26 | 1.59 | Undisturbed | | 1 | SLA |
| 2.15 | 0.00 | 1.15 | Undisturbed | | 1 | SLA |
| 2.32 | 1.78 | 1.56 | Undisturbed | | 1 | SLA |
| 2.45 | 2.82 | 1.88 | Undisturbed | | 1 | SLA |
| 2.22 | 2.49 | 1.33 | Undisturbed | | 1 | SLA |
| 2.21 | 1.11 | 1.30 | Undisturbed | | 1 | SLA |
| 2.27 | 0.48 | 1.45 | Undisturbed | | 1 | SLA |
| 2.29 | 2.49 | 1.49 | Undisturbed | | 1 | SLA |
| 2.10 | 1.77 | 1.03 | Undisturbed | | 1 | SLA |
| 2.33 | 1.71 | 1.58 | Undisturbed | | 1 | SLA |
| 2.31 | 1.46 | 1.55 | Undisturbed | | 1 | SLA |
| 2.47 | 2.49 | 1.92 | Undisturbed | | 1 | SLA |
| 2.23 | 1.11 | 1.33 | Undisturbed | | 1 | SLA |
| 2.43 | 2.49 | 1.82 | Undisturbed | | 1 | SLA |
| 2.53 | 1.81 | 2.07 | Undisturbed | | 1 | SLA |
| 2.51 | 2.59 | 2.03 | Undisturbed | | 1 | SLA |
| 2.27 | 2.49 | 1.43 | Undisturbed | | 1 | SLA |
| 2.39 | 2.21 | 1.73 | Undisturbed | | 1 | SLA |
| 2.40 | 2.32 | 1.76 | Undisturbed | | 1 | SLA |
| 2.10 | 0.00 | 1.03 | Undisturbed | | 1 | SLA |
| 2.24 | 0.00 | 1.37 | Undisturbed | | 1 | SLA |
| 2.15 | 2.30 | 1.14 | Undisturbed | | 1 | SLA |
| 2.24 | 2.47 | 1.37 | Undisturbed | | 1 | SLA |
| 2.63 | 2.17 | 2.31 | Undisturbed | | 1 | SLA |
| 2.26 | 1.59 | 1.41 | Undisturbed | | 1 | SLA |
| 2.18 | 1.95 | 1.23 | Undisturbed | | 1 | SLA |
| 2.65 | 2.70 | 2.35 | Undisturbed | | 1 | SLA |
| 2.41 | 2.28 | 1.79 | Undisturbed | | 1 | SLA |
| 2.57 | 1.88 | 2.18 | Undisturbed | | 1 | SLA |
| 2.23 | 0.78 | 1.34 | Undisturbed | | 1 | SLA |
| 2.32 | 1.79 | 1.56 | Undisturbed | | 1 | SLA |
| 2.45 | 1.04 | 1.88 | Undisturbed | | 1 | SLA |
| 2.20 | 1.04 | 1.28 | Undisturbed | | 1 | SLA |
| 2.20 | 0.30 | 1.28 | Undisturbed | | 1 | SLA |
| 2.48 | 0.30 | 1.94 | Undisturbed | | 1 | SLA |
| 2.37 | 2.16 | 1.67 | Undisturbed | | 1 | SLA |
| 2.67 | 3.00 | 2.42 | Undisturbed | | 1 | SLA |
| 2.43 | 1.42 | 1.83 | Undisturbed | | 1 | SLA |
| 2.25 | 1.04 | 1.40 | Undisturbed | | 1 | SLA |
| 2.32 | 1.42 | 1.57 | Undisturbed | | 1 | SLA |
| 2.41 | 0.30 | 1.78 | Undisturbed | | 1 | SLA |
| 2.27 | 1.54 | 1.45 | Undisturbed | | 1 | SLA |
| 2.55 | 0.00 | 2.13 | Undisturbed | | 1 | SLA |
| 2.38 | 2.08 | 1.70 | Undisturbed | | 1 | SLA |
| 2.53 | 2.36 | 2.07 | Undisturbed | | 1 | SLA |
| 2.15 | 0.48 | 1.15 | Undisturbed | | 1 | SLA |
| 2.13 | 1.49 | 1.10 | Undisturbed | | 1 | SLA |
| 2.17 | 0.30 | 1.20 | Undisturbed | | 1 | SLA |
| 2.49 | 0.85 | 1.97 | Undisturbed | | 1 | SLA |
| 2.48 | 2.49 | 1.96 | Undisturbed | | 1 | SLA |
| 2.57 | 0.00 | 1.02 | 4 | | 2 | SLA |
| 2.41 | 1.72 | 1.39 | 4 | | 2 | SLA |
| 2.52 | 0.95 | 1.15 | 4 | | 2 | SLA |
| 2.52 | 1.00 | 1.15 | 4 | | 2 | SLA |
| 2.34 | 1.42 | 1.59 | 4 | | 2 | SLA |
| 2.34 | 2.62 | 1.57 | 4 | | 2 | SLA |
| 2.44 | 1.76 | 1.34 | 4 | | 2 | SLA |
| 2.49 | 0.00 | 1.20 | 4 | | 2 | SLA |
| 2.58 | 2.03 | 0.98 | 4 | | 2 | SLA |
| 2.29 | 0.60 | 1.69 | 4 | | 2 | SLA |
| 1.96 | 3.02 | 2.52 | 4 | | 2 | SLA |
| 2.44 | 0.00 | 1.34 | 4 | | 2 | SLA |
| 2.50 | 1.53 | 1.18 | 4 | | 2 | SLA |
| 2.35 | 1.52 | 1.55 | 4 | | 2 | SLA |
| 2.51 | 0.00 | 1.16 | 4 | | 2 | SLA |
| 2.27 | 1.00 | 1.76 | 4 | | 2 | SLA |
| 2.43 | 1.40 | 1.35 | 4 | | 2 | SLA |
| 2.55 | 0.48 | 1.07 | 4 | | 2 | SLA |
| 2.61 | 2.79 | 0.90 | 4 | | 2 | SLA |
| 2.57 | 2.71 | 1.00 | 4 | | 2 | SLA |
| 2.56 | 1.88 | 1.02 | 4 | | 2 | SLA |
| 2.49 | 0.00 | 1.22 | 4 | | 2 | SLA |
| 2.54 | 0.00 | 1.07 | 4 | | 2 | SLA |
| 2.52 | 1.80 | 1.14 | 4 | | 2 | SLA |
| 2.57 | 1.11 | 1.00 | 4 | | 2 | SLA |
| 2.45 | 1.99 | 1.31 | 4 | | 2 | SLA |
| 2.54 | 0.30 | 1.08 | 4 | | 2 | SLA |
| 2.60 | 1.04 | 0.93 | 4 | | 2 | SLA |
| 2.57 | 0.30 | 1.02 | 4 | | 2 | SLA |
| 2.35 | 2.74 | 1.57 | 4 | | 2 | SLA |
| 2.50 | 0.60 | 1.19 | 4 | | 2 | SLA |
| 2.53 | 1.46 | 1.10 | 4 | | 2 | SLA |
| 2.52 | 0.95 | 1.14 | 4 | | 2 | SLA |
| 2.44 | 1.93 | 1.33 | 4 | | 2 | SLA |
| 2.50 | 1.83 | 1.18 | 4 | | 2 | SLA |
| 2.38 | 2.11 | 1.49 | 4 | | 2 | SLA |
| 2.30 | 2.10 | 1.67 | 4 | | 2 | SLA |
| 2.44 | 0.00 | 1.32 | 4 | | 2 | SLA |
| 2.44 | 1.26 | 1.36 | 6 | | 2 | SLA |
| 2.41 | 1.98 | 1.47 | 6 | | 2 | SLA |
| 2.45 | 2.32 | 1.36 | 6 | | 2 | SLA |
| 2.13 | 2.66 | 2.36 | 6 | | 2 | SLA |
| 2.62 | 0.95 | 0.79 | 6 | | 2 | SLA |
| 2.70 | 0.00 | 0.53 | 6 | | 2 | SLA |
| 2.72 | 0.00 | 0.49 | 6 | | 2 | SLA |
| 2.37 | 0.48 | 1.60 | 6 | | 2 | SLA |
| 2.48 | 1.52 | 1.23 | 6 | | 2 | SLA |
| 2.39 | 1.43 | 1.54 | 6 | | 2 | SLA |
| 2.54 | 1.18 | 1.06 | 6 | | 2 | SLA |
| 2.42 | 1.00 | 1.43 | 6 | | 2 | SLA |
| 2.24 | 1.34 | 2.03 | 6 | | 2 | SLA |
| 2.52 | 1.57 | 1.10 | 6 | | 2 | SLA |
| 2.49 | 2.36 | 1.21 | 6 | | 2 | SLA |
| 2.15 | 3.00 | 2.29 | 6 | | 2 | SLA |
| 2.16 | 2.90 | 2.26 | 6 | | 2 | SLA |
| 2.29 | 2.21 | 1.85 | 6 | | 2 | SLA |
| 2.42 | 1.43 | 1.43 | 6 | | 2 | SLA |
| 2.59 | 2.04 | 0.89 | 6 | | 2 | SLA |
| 2.46 | 0.60 | 1.30 | 6 | | 2 | SLA |
| 2.31 | 2.03 | 1.78 | 6 | | 2 | SLA |
| 2.27 | 1.43 | 1.91 | 6 | | 2 | SLA |
| 2.30 | 1.00 | 1.84 | 6 | | 2 | SLA |
| 2.47 | 0.30 | 1.28 | 6 | | 2 | SLA |
| 2.44 | 1.04 | 1.38 | 6 | | 2 | SLA |
| 2.46 | 1.46 | 1.30 | 6 | | 2 | SLA |
| 2.53 | 0.30 | 1.08 | 6 | | 2 | SLA |
| 2.34 | 1.26 | 1.68 | 6 | | 2 | SLA |
| 2.56 | 0.00 | 0.99 | 6 | | 2 | SLA |
| 2.64 | 1.72 | 0.71 | 6 | | 2 | SLA |
| 2.53 | 1.81 | 1.08 | 6 | | 2 | SLA |
| 2.36 | 1.81 | 1.73 | 10 | | 2 | SLA |
| 2.14 | 2.73 | 1.68 | 10 | | 2 | SLA |
| 2.31 | 0.30 | 1.72 | 10 | | 2 | SLA |
| 2.54 | 0.70 | 1.77 | 10 | | 2 | SLA |
| 2.59 | 0.70 | 1.78 | 10 | | 2 | SLA |
| 2.45 | 1.88 | 1.75 | 10 | | 2 | SLA |
| 2.04 | 2.79 | 1.65 | 10 | | 2 | SLA |
| 2.28 | 2.76 | 1.71 | 10 | | 2 | SLA |
| 2.30 | 1.90 | 1.71 | 10 | | 2 | SLA |
| 2.39 | 2.55 | 1.73 | 10 | | 2 | SLA |
| 2.55 | 0.00 | 1.77 | 10 | | 2 | SLA |
| 2.41 | 0.48 | 1.74 | 10 | | 2 | SLA |
| 2.42 | 1.97 | 1.74 | 10 | | 2 | SLA |
| 2.41 | 2.45 | 1.74 | 10 | | 2 | SLA |
| 2.38 | 2.05 | 1.73 | 10 | | 2 | SLA |
| 2.25 | 2.27 | 1.70 | 10 | | 2 | SLA |
| 2.43 | 2.27 | 1.74 | 10 | | 2 | SLA |
| 2.13 | 2.47 | 1.68 | 10 | | 2 | SLA |
| 2.22 | 2.72 | 1.69 | 10 | | 2 | SLA |
| 2.51 | 2.63 | 1.76 | 10 | | 2 | SLA |
| 1.93 | 0.00 | 1.63 | 10 | | 2 | SLA |
| 2.28 | 2.48 | 1.71 | 10 | | 2 | SLA |
| 2.57 | 2.57 | 1.78 | 10 | | 2 | SLA |
| 2.45 | 1.26 | 1.75 | 10 | | 2 | SLA |
| 2.33 | 2.23 | 1.72 | 10 | | 2 | SLA |
| 2.40 | 1.83 | 1.74 | 10 | | 2 | SLA |
| 2.67 | 2.87 | 1.80 | 10 | | 2 | SLA |
| 2.10 | 1.20 | 1.67 | 10 | | 2 | SLA |
| 1.27 | 0.30 | 1.48 | 10 | | 2 | SLA |
| 2.59 | 2.51 | 1.78 | 10 | | 2 | SLA |
| 2.29 | 2.00 | 1.71 | 10 | | 2 | SLA |
| 2.58 | 2.09 | 1.78 | 10 | | 2 | SLA |
| 2.34 | 2.31 | 1.72 | 10 | | 2 | SLA |
| 2.18 | 1.11 | 1.69 | 10 | | 2 | SLA |
| 2.16 | 0.48 | 1.68 | 10 | | 2 | SLA |
| 2.61 | 2.02 | 1.79 | 10 | | 2 | SLA |
| 2.32 | 2.21 | 1.72 | 10 | | 2 | SLA |
| 2.40 | 2.38 | 1.74 | 10 | | 2 | SLA |
| 2.24 | 1.04 | 1.70 | 10 | | 2 | SLA |
| 2.45 | 1.71 | 1.75 | 10 | | 2 | SLA |
| 2.50 | 0.78 | 1.76 | 10 | | 2 | SLA |
| 2.46 | 0.85 | 1.75 | 10 | | 2 | SLA |
| 2.54 | 2.67 | 1.77 | 10 | | 2 | SLA |
| 2.42 | 1.91 | 1.74 | 10 | | 2 | SLA |
| 2.17 | 1.49 | 1.68 | 10 | | 2 | SLA |
| 2.77 | 0.00 | 1.82 | 10 | | 2 | SLA |
| 2.72 | 0.00 | 1.81 | 10 | | 2 | SLA |
| 2.40 | 2.04 | 1.74 | 10 | | 2 | SLA |
| 2.23 | 2.85 | 1.70 | 10 | | 2 | SLA |
| 2.43 | 1.11 | 1.93 | 13 | | 2 | SLA |
| 2.42 | 1.86 | 1.92 | 13 | | 2 | SLA |
| 2.63 | 2.76 | 2.31 | 13 | | 2 | SLA |
| 2.46 | 2.56 | 1.99 | 13 | | 2 | SLA |
| 2.49 | 1.40 | 2.04 | 13 | | 2 | SLA |
| 1.94 | 0.95 | 1.00 | 13 | | 2 | SLA |
| 2.38 | 2.03 | 1.83 | 13 | | 2 | SLA |
| 2.53 | 1.89 | 2.11 | 13 | | 2 | SLA |
| 2.54 | 2.14 | 2.14 | 13 | | 2 | SLA |
| 2.58 | 2.30 | 2.22 | 13 | | 2 | SLA |
| 2.47 | 1.90 | 2.00 | 13 | | 2 | SLA |
| 2.76 | 2.81 | 2.56 | 13 | | 2 | SLA |
| 2.45 | 2.29 | 1.97 | 13 | | 2 | SLA |
| 2.48 | 2.55 | 2.03 | 13 | | 2 | SLA |
| 2.41 | 2.53 | 1.89 | 13 | | 2 | SLA |
| 2.42 | 2.15 | 1.90 | 13 | | 2 | SLA |
| 2.57 | 1.40 | 2.19 | 13 | | 2 | SLA |
| 2.56 | 2.68 | 2.18 | 13 | | 2 | SLA |
| 2.32 | 1.88 | 1.71 | 13 | | 2 | SLA |
| 2.34 | 2.77 | 1.75 | 13 | | 2 | SLA |
| 2.53 | 2.27 | 2.12 | 13 | | 2 | SLA |
| 2.51 | 1.86 | 2.09 | 13 | | 2 | SLA |
| 2.49 | 1.80 | 2.04 | 13 | | 2 | SLA |
| 2.36 | 2.59 | 1.79 | 13 | | 2 | SLA |
| 2.33 | 1.11 | 1.74 | 13 | | 2 | SLA |
| 2.27 | 1.04 | 1.63 | 13 | | 2 | SLA |
| 2.47 | 1.30 | 2.00 | 13 | | 2 | SLA |
| 2.52 | 1.84 | 2.09 | 13 | | 2 | SLA |
| 2.49 | 1.89 | 2.04 | 13 | | 2 | SLA |
| 2.75 | 2.93 | 2.53 | 13 | | 2 | SLA |
| 2.51 | 1.96 | 2.09 | 13 | | 2 | SLA |
| 2.58 | 1.91 | 2.21 | 13 | | 2 | SLA |
| 2.33 | 1.45 | 1.73 | 13 | | 2 | SLA |
| 2.54 | 1.43 | 2.13 | 13 | | 2 | SLA |
| 2.39 | 2.16 | 1.84 | 13 | | 2 | SLA |
| 2.07 | 1.45 | 1.24 | 13 | | 2 | SLA |
| 2.08 | 1.18 | 1.26 | 13 | | 2 | SLA |
| 2.39 | 2.52 | 1.85 | 13 | | 2 | SLA |
| 2.38 | 1.26 | 1.83 | 13 | | 2 | SLA |
| 2.23 | 1.96 | 1.38 | undisturbed | | 2 | SLA |
| 2.43 | 2.62 | 2.05 | undisturbed | | 2 | SLA |
| 2.29 | 0.00 | 1.59 | undisturbed | | 2 | SLA |
| 2.15 | 0.00 | 1.12 | undisturbed | | 2 | SLA |
| 2.08 | 0.48 | 0.91 | undisturbed | | 2 | SLA |
| 2.27 | 1.83 | 1.53 | undisturbed | | 2 | SLA |
| 2.45 | 2.64 | 2.14 | undisturbed | | 2 | SLA |
| 2.22 | 2.63 | 1.38 | undisturbed | | 2 | SLA |
| 2.21 | 1.46 | 1.33 | undisturbed | | 2 | SLA |
| 2.27 | 0.30 | 1.54 | undisturbed | | 2 | SLA |
| 2.29 | 2.19 | 1.60 | undisturbed | | 2 | SLA |
| 2.10 | 2.15 | 0.96 | undisturbed | | 2 | SLA |
| 2.33 | 1.94 | 1.71 | undisturbed | | 2 | SLA |
| 2.31 | 2.18 | 1.68 | undisturbed | | 2 | SLA |
| 2.47 | 2.77 | 2.19 | undisturbed | | 2 | SLA |
| 2.23 | 1.18 | 1.38 | undisturbed | | 2 | SLA |
| 2.43 | 2.51 | 2.06 | undisturbed | | 2 | SLA |
| 2.53 | 2.20 | 2.39 | undisturbed | | 2 | SLA |
| 2.24 | 0.00 | 1.43 | undisturbed | | 2 | SLA |
| 2.51 | 2.54 | 2.35 | undisturbed | | 2 | SLA |
| 2.27 | 2.01 | 1.51 | undisturbed | | 2 | SLA |
| 2.39 | 2.26 | 1.92 | undisturbed | | 2 | SLA |
| 2.40 | 2.49 | 1.97 | undisturbed | | 2 | SLA |
| 1.98 | 0.00 | 0.57 | undisturbed | | 2 | SLA |
| 1.87 | 0.00 | 0.19 | undisturbed | | 2 | SLA |
| 2.53 | 2.37 | 2.41 | undisturbed | | 2 | SLA |
| 2.24 | 2.12 | 1.44 | undisturbed | | 2 | SLA |
| 2.10 | 1.99 | 0.96 | undisturbed | | 2 | SLA |
| 2.26 | 1.86 | 1.49 | undisturbed | | 2 | SLA |
| 2.18 | 1.88 | 1.24 | undisturbed | | 2 | SLA |
| 2.65 | 2.74 | 2.79 | undisturbed | | 2 | SLA |
| 2.54 | 2.60 | 2.43 | undisturbed | | 2 | SLA |
| 1.88 | 0.00 | 0.20 | undisturbed | | 2 | SLA |
| 2.32 | 1.81 | 1.70 | undisturbed | | 2 | SLA |
| 1.91 | 0.30 | 0.33 | undisturbed | | 2 | SLA |
| 2.00 | 0.48 | 0.61 | undisturbed | | 2 | SLA |
| 1.78 | 0.00 | -0.13 | undisturbed | | 2 | SLA |
| 2.48 | 0.78 | 2.22 | undisturbed | | 2 | SLA |
| 2.37 | 2.00 | 1.85 | undisturbed | | 2 | SLA |
| 2.43 | 2.93 | 2.08 | undisturbed | | 2 | SLA |
| 2.43 | 1.00 | 2.07 | undisturbed | | 2 | SLA |
| 2.25 | 1.18 | 1.48 | undisturbed | | 2 | SLA |
| 2.32 | 2.00 | 1.71 | undisturbed | | 2 | SLA |
| 2.41 | 0.00 | 2.00 | undisturbed | | 2 | SLA |
| 2.29 | 2.20 | 1.61 | undisturbed | | 2 | SLA |
| 2.38 | 1.95 | 1.89 | undisturbed | | 2 | SLA |
| 2.53 | 2.14 | 2.39 | undisturbed | | 2 | SLA |
| 2.13 | 1.20 | 1.05 | undisturbed | | 2 | SLA |
| 2.17 | 0.00 | 1.20 | undisturbed | | 2 | SLA |
| 2.48 | 2.27 | 2.24 | undisturbed | | 2 | SLA |
| -0.10 | 1.92 | 1.26 | 4 | | 1 | SM |
| -0.24 | 2.00 | 1.30 | 4 | | 1 | SM |
| 0.26 | 1.30 | 1.17 | 4 | | 1 | SM |
| -0.41 | 1.92 | 1.34 | 4 | | 1 | SM |
| -1.05 | 1.52 | 1.50 | 4 | | 1 | SM |
| -0.35 | 2.40 | 1.32 | 4 | | 1 | SM |
| -0.15 | 1.92 | 1.27 | 4 | | 1 | SM |
| 0.53 | 0.85 | 1.10 | 4 | | 1 | SM |
| 0.72 | 0.00 | 1.06 | 4 | | 1 | SM |
| 0.09 | 0.00 | 1.21 | 4 | | 1 | SM |
| 0.17 | 2.29 | 1.19 | 4 | | 1 | SM |
| -0.29 | 1.65 | 1.31 | 4 | | 1 | SM |
| -0.28 | 0.00 | 1.31 | 4 | | 1 | SM |
| -0.22 | 2.84 | 1.29 | 4 | | 1 | SM |
| 0.52 | 0.30 | 1.11 | 4 | | 1 | SM |
| -0.04 | 1.69 | 1.25 | 4 | | 1 | SM |
| -0.48 | 0.78 | 1.36 | 4 | | 1 | SM |
| -1.43 | 0.30 | 1.59 | 4 | | 1 | SM |
| 0.13 | 0.48 | 1.20 | 4 | | 1 | SM |
| -1.11 | 1.46 | 1.51 | 4 | | 1 | SM |
| -0.53 | 1.79 | 1.37 | 4 | | 1 | SM |
| 0.06 | 2.57 | 1.22 | 4 | | 1 | SM |
| 0.08 | 0.30 | 1.22 | 4 | | 1 | SM |
| 0.68 | 1.79 | 1.07 | 4 | | 1 | SM |
| -4.05 | 2.29 | 2.24 | 4 | | 1 | SM |
| -0.77 | 0.00 | 1.43 | 4 | | 1 | SM |
| 0.33 | 0.60 | 1.15 | 4 | | 1 | SM |
| 0.30 | 0.30 | 1.16 | 4 | | 1 | SM |
| 0.20 | 0.85 | 1.19 | 4 | | 1 | SM |
| -0.59 | 3.10 | 1.38 | 4 | | 1 | SM |
| 0.62 | 1.90 | 1.08 | 4 | | 1 | SM |
| -0.11 | 0.48 | 1.26 | 4 | | 1 | SM |
| -1.07 | 2.48 | 1.50 | 4 | | 1 | SM |
| 0.34 | 0.00 | 1.15 | 4 | | 1 | SM |
| 0.16 | 1.95 | 1.20 | 4 | | 1 | SM |
| 0.10 | 2.24 | 1.21 | 4 | | 1 | SM |
| 0.94 | 0.48 | 1.00 | 4 | | 1 | SM |
| -0.95 | 2.29 | 1.47 | 4 | | 1 | SM |
| 0.34 | 0.30 | 1.15 | 4 | | 1 | SM |
| 0.28 | 0.00 | 1.17 | 4 | | 1 | SM |
| 1.25 | 1.96 | 0.93 | 4 | | 1 | SM |
| -0.54 | 1.91 | 1.37 | 4 | | 1 | SM |
| 0.35 | 0.70 | 1.15 | 4 | | 1 | SM |
| -0.35 | 1.26 | 1.32 | 4 | | 1 | SM |
| -0.16 | 0.00 | 1.28 | 4 | | 1 | SM |
| 0.52 | 1.08 | 1.11 | 4 | | 1 | SM |
| 0.30 | 1.54 | 1.16 | 4 | | 1 | SM |
| 0.61 | 1.32 | 1.08 | 4 | | 1 | SM |
| -0.20 | 0.30 | 1.29 | 4 | | 1 | SM |
| 1.02 | 0.70 | 0.98 | 4 | | 1 | SM |
| 0.09 | 2.89 | 1.21 | 4 | | 1 | SM |
| 0.34 | 2.16 | 1.15 | 4 | | 1 | SM |
| -0.84 | 1.18 | 1.45 | 4 | | 1 | SM |
| -0.90 | 1.89 | 1.46 | 4 | | 1 | SM |
| 1.18 | 1.69 | 0.95 | 4 | | 1 | SM |
| 0.08 | 1.99 | 1.22 | 4 | | 1 | SM |
| -0.15 | 0.00 | 1.27 | 4 | | 1 | SM |
| -0.63 | 0.30 | 1.39 | 4 | | 1 | SM |
| 0.26 | 0.00 | 1.17 | 4 | | 1 | SM |
| -0.11 | 2.22 | 1.39 | 6 | | 1 | SM |
| -0.10 | 0.78 | 1.38 | 6 | | 1 | SM |
| -0.67 | 1.86 | 1.52 | 6 | | 1 | SM |
| -0.24 | 0.48 | 1.42 | 6 | | 1 | SM |
| -0.41 | 2.18 | 1.46 | 6 | | 1 | SM |
| -1.05 | 2.05 | 1.61 | 6 | | 1 | SM |
| -0.35 | 2.04 | 1.44 | 6 | | 1 | SM |
| 0.53 | 2.62 | 1.23 | 6 | | 1 | SM |
| 0.72 | 0.90 | 1.19 | 6 | | 1 | SM |
| -0.56 | 1.00 | 1.49 | 6 | | 1 | SM |
| 0.17 | 0.48 | 1.32 | 6 | | 1 | SM |
| -0.29 | 0.60 | 1.43 | 6 | | 1 | SM |
| 0.21 | 2.03 | 1.31 | 6 | | 1 | SM |
| -0.28 | 0.48 | 1.43 | 6 | | 1 | SM |
| -0.22 | 0.30 | 1.41 | 6 | | 1 | SM |
| 0.52 | 0.30 | 1.24 | 6 | | 1 | SM |
| -0.04 | 1.88 | 1.37 | 6 | | 1 | SM |
| -0.48 | 1.84 | 1.48 | 6 | | 1 | SM |
| -1.43 | 0.70 | 1.70 | 6 | | 1 | SM |
| 0.13 | 1.68 | 1.33 | 6 | | 1 | SM |
| -1.11 | 1.04 | 1.63 | 6 | | 1 | SM |
| -0.53 | 1.40 | 1.49 | 6 | | 1 | SM |
| 0.06 | 1.70 | 1.35 | 6 | | 1 | SM |
| 0.08 | 1.04 | 1.34 | 6 | | 1 | SM |
| 0.68 | 0.00 | 1.20 | 6 | | 1 | SM |
| -4.05 | 2.30 | 2.33 | 6 | | 1 | SM |
| 0.33 | 2.26 | 1.28 | 6 | | 1 | SM |
| 0.30 | 1.18 | 1.29 | 6 | | 1 | SM |
| 0.20 | 1.23 | 1.31 | 6 | | 1 | SM |
| -0.25 | 3.01 | 1.42 | 6 | | 1 | SM |
| 0.62 | 1.95 | 1.21 | 6 | | 1 | SM |
| -1.07 | 2.71 | 1.62 | 6 | | 1 | SM |
| 0.34 | 2.02 | 1.28 | 6 | | 1 | SM |
| 0.16 | 1.99 | 1.32 | 6 | | 1 | SM |
| 0.10 | 2.57 | 1.34 | 6 | | 1 | SM |
| 0.94 | 1.54 | 1.13 | 6 | | 1 | SM |
| -0.95 | 1.60 | 1.59 | 6 | | 1 | SM |
| 0.34 | 0.00 | 1.28 | 6 | | 1 | SM |
| 0.28 | 2.05 | 1.29 | 6 | | 1 | SM |
| 1.25 | 0.00 | 1.06 | 6 | | 1 | SM |
| -0.54 | 2.14 | 1.49 | 6 | | 1 | SM |
| 0.35 | 1.08 | 1.28 | 6 | | 1 | SM |
| -0.35 | 1.89 | 1.44 | 6 | | 1 | SM |
| 1.34 | 1.20 | 1.04 | 6 | | 1 | SM |
| -0.63 | 1.00 | 1.51 | 6 | | 1 | SM |
| 0.52 | 1.18 | 1.23 | 6 | | 1 | SM |
| -0.89 | 1.30 | 1.57 | 6 | | 1 | SM |
| 0.30 | 1.54 | 1.29 | 6 | | 1 | SM |
| 0.61 | 1.38 | 1.21 | 6 | | 1 | SM |
| -0.20 | 1.71 | 1.41 | 6 | | 1 | SM |
| 0.05 | 0.78 | 1.35 | 6 | | 1 | SM |
| 1.02 | 0.30 | 1.12 | 6 | | 1 | SM |
| 0.52 | 1.00 | 1.23 | 6 | | 1 | SM |
| -0.13 | 1.04 | 1.39 | 6 | | 1 | SM |
| 0.09 | 2.32 | 1.34 | 6 | | 1 | SM |
| 0.34 | 1.53 | 1.28 | 6 | | 1 | SM |
| -0.84 | 1.04 | 1.56 | 6 | | 1 | SM |
| -0.90 | 1.38 | 1.58 | 6 | | 1 | SM |
| 1.18 | 0.70 | 1.08 | 6 | | 1 | SM |
| 0.08 | 1.59 | 1.34 | 6 | | 1 | SM |
| -0.15 | 0.30 | 1.40 | 6 | | 1 | SM |
| -0.63 | 1.11 | 1.51 | 6 | | 1 | SM |
| -0.11 | 2.20 | 1.39 | 10 | | 1 | SM |
| -0.67 | 1.34 | 1.23 | 10 | | 1 | SM |
| 0.09 | 0.90 | 1.45 | 10 | | 1 | SM |
| -0.24 | 0.00 | 1.35 | 10 | | 1 | SM |
| -1.04 | 0.00 | 1.13 | 10 | | 1 | SM |
| 0.26 | 0.70 | 1.50 | 10 | | 1 | SM |
| -0.05 | 1.66 | 1.41 | 10 | | 1 | SM |
| -0.41 | 1.52 | 1.30 | 10 | | 1 | SM |
| -1.05 | 0.30 | 1.12 | 10 | | 1 | SM |
| -0.35 | 1.20 | 1.32 | 10 | | 1 | SM |
| -0.15 | 0.00 | 1.38 | 10 | | 1 | SM |
| 0.53 | 1.80 | 1.57 | 10 | | 1 | SM |
| 0.72 | 2.16 | 1.63 | 10 | | 1 | SM |
| 0.09 | 0.30 | 1.45 | 10 | | 1 | SM |
| 0.17 | 2.50 | 1.47 | 10 | | 1 | SM |
| 0.46 | 0.00 | 1.55 | 10 | | 1 | SM |
| -0.29 | 0.95 | 1.34 | 10 | | 1 | SM |
| 0.21 | 1.59 | 1.48 | 10 | | 1 | SM |
| -0.28 | 0.30 | 1.34 | 10 | | 1 | SM |
| -0.22 | 1.68 | 1.36 | 10 | | 1 | SM |
| 0.52 | 0.60 | 1.57 | 10 | | 1 | SM |
| -0.04 | 0.00 | 1.41 | 10 | | 1 | SM |
| -0.29 | 1.43 | 1.34 | 10 | | 1 | SM |
| -0.48 | 2.05 | 1.29 | 10 | | 1 | SM |
| 0.13 | 1.94 | 1.46 | 10 | | 1 | SM |
| -1.11 | 1.58 | 1.11 | 10 | | 1 | SM |
| -1.43 | 1.20 | 1.01 | 10 | | 1 | SM |
| -0.53 | 2.34 | 1.27 | 10 | | 1 | SM |
| 0.06 | 1.80 | 1.44 | 10 | | 1 | SM |
| 0.08 | 1.81 | 1.45 | 10 | | 1 | SM |
| -4.05 | 0.95 | 0.27 | 10 | | 1 | SM |
| -0.77 | 0.00 | 1.20 | 10 | | 1 | SM |
| -0.09 | 1.65 | 1.40 | 10 | | 1 | SM |
| 0.30 | 2.96 | 1.51 | 10 | | 1 | SM |
| 0.20 | 1.92 | 1.48 | 10 | | 1 | SM |
| 0.41 | 2.47 | 1.54 | 10 | | 1 | SM |
| -0.19 | 1.51 | 1.37 | 10 | | 1 | SM |
| -0.11 | 1.04 | 1.39 | 10 | | 1 | SM |
| -1.07 | 1.98 | 1.12 | 10 | | 1 | SM |
| 0.34 | 2.11 | 1.52 | 10 | | 1 | SM |
| 0.16 | 2.11 | 1.47 | 10 | | 1 | SM |
| 0.10 | 2.50 | 1.45 | 10 | | 1 | SM |
| -0.95 | 1.32 | 1.15 | 10 | | 1 | SM |
| 0.34 | 1.36 | 1.52 | 10 | | 1 | SM |
| 0.28 | 1.87 | 1.50 | 10 | | 1 | SM |
| -0.76 | 0.48 | 1.21 | 10 | | 1 | SM |
| 1.25 | 3.16 | 1.78 | 10 | | 1 | SM |
| -0.54 | 1.26 | 1.27 | 10 | | 1 | SM |
| 0.35 | 0.90 | 1.52 | 10 | | 1 | SM |
| -0.35 | 2.22 | 1.32 | 10 | | 1 | SM |
| -0.16 | 0.00 | 1.38 | 10 | | 1 | SM |
| 1.34 | 2.52 | 1.80 | 10 | | 1 | SM |
| -0.63 | 1.00 | 1.24 | 10 | | 1 | SM |
| 0.52 | 1.08 | 1.57 | 10 | | 1 | SM |
| -0.89 | 1.42 | 1.17 | 10 | | 1 | SM |
| 0.30 | 1.68 | 1.51 | 10 | | 1 | SM |
| 0.61 | 1.15 | 1.60 | 10 | | 1 | SM |
| -0.20 | 2.89 | 1.37 | 10 | | 1 | SM |
| 0.05 | 1.30 | 1.44 | 10 | | 1 | SM |
| 1.02 | 1.87 | 1.71 | 10 | | 1 | SM |
| 0.52 | 1.84 | 1.57 | 10 | | 1 | SM |
| -0.55 | 1.26 | 1.27 | 10 | | 1 | SM |
| -0.13 | 1.30 | 1.38 | 10 | | 1 | SM |
| 0.09 | 1.36 | 1.45 | 10 | | 1 | SM |
| 0.34 | 1.60 | 1.52 | 10 | | 1 | SM |
| -0.84 | 2.47 | 1.18 | 10 | | 1 | SM |
| -0.90 | 0.30 | 1.17 | 10 | | 1 | SM |
| -0.15 | 0.85 | 1.38 | 10 | | 1 | SM |
| 0.55 | 1.04 | 1.58 | 10 | | 1 | SM |
| -0.63 | 1.52 | 1.24 | 10 | | 1 | SM |
| 0.26 | 0.00 | 1.49 | 10 | | 1 | SM |
| -0.11 | 2.56 | 1.44 | 13 | | 1 | SM |
| -0.67 | 0.48 | 1.44 | 13 | | 1 | SM |
| -0.16 | 1.18 | 1.44 | 13 | | 1 | SM |
| 0.09 | 1.62 | 1.44 | 13 | | 1 | SM |
| -0.24 | 1.67 | 1.44 | 13 | | 1 | SM |
| -1.04 | 0.48 | 1.44 | 13 | | 1 | SM |
| -0.19 | 1.08 | 1.44 | 13 | | 1 | SM |
| -0.05 | 1.96 | 1.44 | 13 | | 1 | SM |
| -0.41 | 1.23 | 1.44 | 13 | | 1 | SM |
| -0.35 | 0.70 | 1.44 | 13 | | 1 | SM |
| -0.15 | 1.40 | 1.44 | 13 | | 1 | SM |
| 0.53 | 1.94 | 1.43 | 13 | | 1 | SM |
| 0.72 | 1.72 | 1.43 | 13 | | 1 | SM |
| 0.09 | 2.81 | 1.44 | 13 | | 1 | SM |
| 0.17 | 2.35 | 1.44 | 13 | | 1 | SM |
| -0.29 | 0.90 | 1.44 | 13 | | 1 | SM |
| 0.21 | 1.52 | 1.44 | 13 | | 1 | SM |
| -0.28 | 0.95 | 1.44 | 13 | | 1 | SM |
| -0.22 | 2.08 | 1.44 | 13 | | 1 | SM |
| -0.29 | 0.60 | 1.44 | 13 | | 1 | SM |
| -0.26 | 0.00 | 1.44 | 13 | | 1 | SM |
| -0.23 | 0.30 | 1.44 | 13 | | 1 | SM |
| -0.48 | 1.20 | 1.44 | 13 | | 1 | SM |
| 0.13 | 1.54 | 1.44 | 13 | | 1 | SM |
| -1.11 | 2.42 | 1.44 | 13 | | 1 | SM |
| -0.53 | 1.87 | 1.44 | 13 | | 1 | SM |
| 0.06 | 2.11 | 1.44 | 13 | | 1 | SM |
| 0.08 | 1.11 | 1.44 | 13 | | 1 | SM |
| -4.05 | 2.30 | 1.46 | 13 | | 1 | SM |
| -0.09 | 1.74 | 1.44 | 13 | | 1 | SM |
| 0.30 | 2.78 | 1.44 | 13 | | 1 | SM |
| 0.20 | 2.06 | 1.44 | 13 | | 1 | SM |
| 0.41 | 2.41 | 1.44 | 13 | | 1 | SM |
| -0.19 | 0.70 | 1.44 | 13 | | 1 | SM |
| -1.07 | 2.38 | 1.44 | 13 | | 1 | SM |
| 0.34 | 1.98 | 1.44 | 13 | | 1 | SM |
| 0.16 | 0.30 | 1.44 | 13 | | 1 | SM |
| 0.10 | 2.65 | 1.44 | 13 | | 1 | SM |
| 0.94 | 0.60 | 1.43 | 13 | | 1 | SM |
| -0.95 | 2.64 | 1.44 | 13 | | 1 | SM |
| 0.34 | 1.67 | 1.44 | 13 | | 1 | SM |
| 0.28 | 1.72 | 1.44 | 13 | | 1 | SM |
| -0.76 | 0.78 | 1.44 | 13 | | 1 | SM |
| 1.25 | 2.47 | 1.43 | 13 | | 1 | SM |
| -0.54 | 2.09 | 1.44 | 13 | | 1 | SM |
| 0.35 | 0.48 | 1.44 | 13 | | 1 | SM |
| -0.16 | 0.30 | 1.44 | 13 | | 1 | SM |
| 1.34 | 2.55 | 1.43 | 13 | | 1 | SM |
| -0.62 | 0.00 | 1.44 | 13 | | 1 | SM |
| 0.52 | 1.00 | 1.43 | 13 | | 1 | SM |
| -0.89 | 1.70 | 1.44 | 13 | | 1 | SM |
| 0.30 | 1.85 | 1.44 | 13 | | 1 | SM |
| 0.51 | 0.90 | 1.43 | 13 | | 1 | SM |
| 0.61 | 2.95 | 1.43 | 13 | | 1 | SM |
| -0.20 | 1.30 | 1.44 | 13 | | 1 | SM |
| 0.05 | 1.74 | 1.44 | 13 | | 1 | SM |
| 1.02 | 1.71 | 1.43 | 13 | | 1 | SM |
| 0.52 | 0.60 | 1.43 | 13 | | 1 | SM |
| -0.55 | 1.11 | 1.44 | 13 | | 1 | SM |
| 0.25 | 0.00 | 1.44 | 13 | | 1 | SM |
| -0.90 | 0.70 | 1.44 | 13 | | 1 | SM |
| -0.13 | 2.11 | 1.44 | 13 | | 1 | SM |
| 0.09 | 1.91 | 1.44 | 13 | | 1 | SM |
| 0.17 | 1.30 | 1.44 | 13 | | 1 | SM |
| 0.34 | 1.48 | 1.44 | 13 | | 1 | SM |
| -0.84 | 2.20 | 1.44 | 13 | | 1 | SM |
| -0.90 | 1.48 | 1.44 | 13 | | 1 | SM |
| 0.86 | 0.60 | 1.43 | 13 | | 1 | SM |
| -0.15 | 0.95 | 1.44 | 13 | | 1 | SM |
| 0.55 | 0.48 | 1.43 | 13 | | 1 | SM |
| -0.63 | 1.04 | 1.44 | 13 | | 1 | SM |
| 0.26 | 1.15 | 1.44 | 13 | | 1 | SM |
| 0.25 | 0.30 | 1.44 | 13 | | 1 | SM |
| -0.11 | 2.78 | 1.48 | undisturbed | | 1 | SM |
| -0.30 | 2.12 | 1.38 | undisturbed | | 1 | SM |
| 0.09 | 1.30 | 1.59 | undisturbed | | 1 | SM |
| -0.24 | 2.17 | 1.41 | undisturbed | | 1 | SM |
| -1.04 | 0.00 | 0.98 | undisturbed | | 1 | SM |
| -1.74 | 0.78 | 0.60 | undisturbed | | 1 | SM |
| -0.19 | 1.67 | 1.44 | undisturbed | | 1 | SM |
| -0.05 | 2.49 | 1.51 | undisturbed | | 1 | SM |
| -0.41 | 0.00 | 1.32 | undisturbed | | 1 | SM |
| -0.46 | 0.00 | 1.29 | undisturbed | | 1 | SM |
| -0.15 | 1.78 | 1.46 | undisturbed | | 1 | SM |
| 0.53 | 1.43 | 1.83 | undisturbed | | 1 | SM |
| 0.07 | 1.04 | 1.58 | undisturbed | | 1 | SM |
| 0.09 | 2.82 | 1.59 | undisturbed | | 1 | SM |
| 0.17 | 2.49 | 1.63 | undisturbed | | 1 | SM |
| 0.46 | 1.11 | 1.79 | undisturbed | | 1 | SM |
| -0.29 | 1.42 | 1.38 | undisturbed | | 1 | SM |
| 0.21 | 0.90 | 1.65 | undisturbed | | 1 | SM |
| -0.28 | 0.48 | 1.39 | undisturbed | | 1 | SM |
| 0.20 | 2.49 | 1.65 | undisturbed | | 1 | SM |
| -0.29 | 1.77 | 1.38 | undisturbed | | 1 | SM |
| -0.26 | 0.00 | 1.40 | undisturbed | | 1 | SM |
| -0.48 | 1.71 | 1.28 | undisturbed | | 1 | SM |
| 0.13 | 1.46 | 1.61 | undisturbed | | 1 | SM |
| 0.34 | 2.49 | 1.72 | undisturbed | | 1 | SM |
| -0.53 | 1.11 | 1.25 | undisturbed | | 1 | SM |
| 0.06 | 2.49 | 1.57 | undisturbed | | 1 | SM |
| 0.08 | 1.81 | 1.58 | undisturbed | | 1 | SM |
| 0.32 | 2.59 | 1.71 | undisturbed | | 1 | SM |
| -0.09 | 2.49 | 1.49 | undisturbed | | 1 | SM |
| 0.30 | 2.42 | 1.70 | undisturbed | | 1 | SM |
| 0.20 | 2.21 | 1.65 | undisturbed | | 1 | SM |
| 0.41 | 2.32 | 1.76 | undisturbed | | 1 | SM |
| 0.62 | 0.30 | 1.87 | undisturbed | | 1 | SM |
| -0.19 | 1.93 | 1.44 | undisturbed | | 1 | SM |
| -0.11 | 0.00 | 1.48 | undisturbed | | 1 | SM |
| 0.46 | 2.30 | 1.79 | undisturbed | | 1 | SM |
| 0.34 | 1.61 | 1.72 | undisturbed | | 1 | SM |
| 0.16 | 2.47 | 1.63 | undisturbed | | 1 | SM |
| 0.94 | 1.00 | 2.05 | undisturbed | | 1 | SM |
| -0.95 | 2.17 | 1.02 | undisturbed | | 1 | SM |
| 0.34 | 1.59 | 1.72 | undisturbed | | 1 | SM |
| 0.28 | 1.95 | 1.69 | undisturbed | | 1 | SM |
| -0.76 | 0.48 | 1.13 | undisturbed | | 1 | SM |
| 1.25 | 2.70 | 2.21 | undisturbed | | 1 | SM |
| -0.54 | 2.28 | 1.25 | undisturbed | | 1 | SM |
| 0.35 | 1.88 | 1.73 | undisturbed | | 1 | SM |
| -0.35 | 1.18 | 1.35 | undisturbed | | 1 | SM |
| -0.16 | 0.78 | 1.45 | undisturbed | | 1 | SM |
| 1.34 | 1.79 | 2.26 | undisturbed | | 1 | SM |
| -0.63 | 1.04 | 1.20 | undisturbed | | 1 | SM |
| 0.16 | 1.04 | 1.63 | undisturbed | | 1 | SM |
| -0.62 | 0.30 | 1.21 | undisturbed | | 1 | SM |
| 0.52 | 0.30 | 1.82 | undisturbed | | 1 | SM |
| -0.89 | 1.74 | 1.06 | undisturbed | | 1 | SM |
| 0.30 | 2.16 | 1.70 | undisturbed | | 1 | SM |
| 0.61 | 3.00 | 1.87 | undisturbed | | 1 | SM |
| -0.20 | 1.75 | 1.43 | undisturbed | | 1 | SM |
| 0.05 | 1.42 | 1.57 | undisturbed | | 1 | SM |
| 1.02 | 2.05 | 2.09 | undisturbed | | 1 | SM |
| 0.52 | 1.04 | 1.82 | undisturbed | | 1 | SM |
| -0.55 | 0.00 | 1.24 | undisturbed | | 1 | SM |
| 0.25 | 0.70 | 1.67 | undisturbed | | 1 | SM |
| -0.90 | 1.30 | 1.05 | undisturbed | | 1 | SM |
| -0.13 | 2.59 | 1.47 | undisturbed | | 1 | SM |
| 0.09 | 2.19 | 1.59 | undisturbed | | 1 | SM |
| -0.88 | 0.30 | 1.07 | undisturbed | | 1 | SM |
| 0.17 | 1.54 | 1.63 | undisturbed | | 1 | SM |
| -0.66 | 0.00 | 1.18 | undisturbed | | 1 | SM |
| -0.84 | 2.08 | 1.08 | undisturbed | | 1 | SM |
| -0.90 | 2.36 | 1.05 | undisturbed | | 1 | SM |
| -0.15 | 1.49 | 1.46 | undisturbed | | 1 | SM |
| 0.55 | 0.30 | 1.83 | undisturbed | | 1 | SM |
| -0.63 | 0.85 | 1.20 | undisturbed | | 1 | SM |
| 0.26 | 2.49 | 1.68 | undisturbed | | 1 | SM |
| -1.14 | 0.48 | 0.93 | undisturbed | | 1 | SM |
| -1.49 | 0.00 | 0.73 | undisturbed | | 1 | SM |
| -0.11 | 0.00 | 1.52 | 4 | | 2 | SM |
| -0.10 | 1.72 | 1.51 | 4 | | 2 | SM |
| -0.67 | 2.20 | 1.92 | 4 | | 2 | SM |
| 0.26 | 1.00 | 1.26 | 4 | | 2 | SM |
| -0.41 | 1.42 | 1.74 | 4 | | 2 | SM |
| -1.05 | 2.57 | 2.18 | 4 | | 2 | SM |
| -0.35 | 2.62 | 1.69 | 4 | | 2 | SM |
| 0.53 | 1.08 | 1.07 | 4 | | 2 | SM |
| 0.72 | 0.00 | 0.93 | 4 | | 2 | SM |
| 0.09 | 0.78 | 1.38 | 4 | | 2 | SM |
| -0.19 | 2.31 | 1.58 | 4 | | 2 | SM |
| -0.29 | 2.49 | 1.65 | 4 | | 2 | SM |
| -0.28 | 0.60 | 1.64 | 4 | | 2 | SM |
| -0.22 | 3.02 | 1.60 | 4 | | 2 | SM |
| 0.52 | 0.00 | 1.08 | 4 | | 2 | SM |
| -0.04 | 0.48 | 1.47 | 4 | | 2 | SM |
| -0.29 | 0.30 | 1.65 | 4 | | 2 | SM |
| -0.48 | 1.52 | 1.78 | 4 | | 2 | SM |
| -1.43 | 1.28 | 2.45 | 4 | | 2 | SM |
| 0.13 | 0.70 | 1.35 | 4 | | 2 | SM |
| -1.11 | 1.46 | 2.22 | 4 | | 2 | SM |
| -0.53 | 1.00 | 1.82 | 4 | | 2 | SM |
| 0.06 | 1.83 | 1.40 | 4 | | 2 | SM |
| 0.08 | 0.60 | 1.38 | 4 | | 2 | SM |
| 0.68 | 1.40 | 0.97 | 4 | | 2 | SM |
| 0.33 | 2.79 | 1.21 | 4 | | 2 | SM |
| 0.30 | 0.00 | 1.23 | 4 | | 2 | SM |
| -0.25 | 3.05 | 1.62 | 4 | | 2 | SM |
| 0.62 | 1.88 | 1.01 | 4 | | 2 | SM |
| -0.11 | 1.15 | 1.52 | 4 | | 2 | SM |
| 0.34 | 0.00 | 1.20 | 4 | | 2 | SM |
| 0.16 | 1.80 | 1.33 | 4 | | 2 | SM |
| 0.10 | 2.47 | 1.38 | 4 | | 2 | SM |
| -0.95 | 2.78 | 2.12 | 4 | | 2 | SM |
| 0.25 | 1.20 | 1.27 | 4 | | 2 | SM |
| 0.35 | 0.30 | 1.20 | 4 | | 2 | SM |
| -0.35 | 1.18 | 1.69 | 4 | | 2 | SM |
| -0.16 | 1.23 | 1.56 | 4 | | 2 | SM |
| 0.52 | 1.04 | 1.07 | 4 | | 2 | SM |
| -0.89 | 3.02 | 2.07 | 4 | | 2 | SM |
| 0.30 | 0.48 | 1.23 | 4 | | 2 | SM |
| 0.61 | 1.34 | 1.01 | 4 | | 2 | SM |
| 0.09 | 2.74 | 1.38 | 4 | | 2 | SM |
| 0.12 | 0.60 | 1.36 | 4 | | 2 | SM |
| 0.34 | 2.23 | 1.21 | 4 | | 2 | SM |
| -0.84 | 2.49 | 2.04 | 4 | | 2 | SM |
| -0.90 | 2.59 | 2.08 | 4 | | 2 | SM |
| 1.18 | 1.82 | 0.61 | 4 | | 2 | SM |
| 0.08 | 2.10 | 1.39 | 4 | | 2 | SM |
| -0.15 | 2.29 | 1.55 | 4 | | 2 | SM |
| -0.63 | 2.23 | 1.89 | 4 | | 2 | SM |
| -0.20 | 2.14 | 1.58 | 4 | | 2 | SM |
| 0.34 | 0.00 | 1.21 | 4 | | 2 | SM |
| -0.11 | 1.26 | 1.20 | 6 | | 2 | SM |
| -0.10 | 1.00 | 1.20 | 6 | | 2 | SM |
| -0.67 | 1.52 | 1.22 | 6 | | 2 | SM |
| -0.24 | 0.30 | 1.21 | 6 | | 2 | SM |
| -0.41 | 1.98 | 1.21 | 6 | | 2 | SM |
| -1.05 | 2.32 | 1.24 | 6 | | 2 | SM |
| -0.35 | 1.75 | 1.21 | 6 | | 2 | SM |
| -0.15 | 0.85 | 1.21 | 6 | | 2 | SM |
| 0.53 | 1.15 | 1.18 | 6 | | 2 | SM |
| -0.56 | 0.95 | 1.22 | 6 | | 2 | SM |
| 0.17 | 1.62 | 1.19 | 6 | | 2 | SM |
| 0.21 | 1.48 | 1.19 | 6 | | 2 | SM |
| -0.28 | 0.00 | 1.21 | 6 | | 2 | SM |
| -0.22 | 0.00 | 1.21 | 6 | | 2 | SM |
| 0.52 | 0.48 | 1.18 | 6 | | 2 | SM |
| -0.04 | 1.52 | 1.20 | 6 | | 2 | SM |
| -0.29 | 0.30 | 1.21 | 6 | | 2 | SM |
| -0.48 | 1.43 | 1.22 | 6 | | 2 | SM |
| -1.43 | 1.18 | 1.25 | 6 | | 2 | SM |
| 0.13 | 1.00 | 1.20 | 6 | | 2 | SM |
| -1.11 | 0.90 | 1.24 | 6 | | 2 | SM |
| -0.53 | 1.34 | 1.22 | 6 | | 2 | SM |
| 0.06 | 1.46 | 1.20 | 6 | | 2 | SM |
| 0.08 | 1.57 | 1.20 | 6 | | 2 | SM |
| 0.68 | 0.00 | 1.18 | 6 | | 2 | SM |
| 0.33 | 2.97 | 1.19 | 6 | | 2 | SM |
| 0.30 | 1.20 | 1.19 | 6 | | 2 | SM |
| 0.20 | 1.11 | 1.19 | 6 | | 2 | SM |
| 0.41 | 2.90 | 1.19 | 6 | | 2 | SM |
| 0.62 | 2.21 | 1.18 | 6 | | 2 | SM |
| -0.19 | 0.90 | 1.21 | 6 | | 2 | SM |
| -1.07 | 1.58 | 1.24 | 6 | | 2 | SM |
| -0.11 | 0.48 | 1.20 | 6 | | 2 | SM |
| 0.34 | 1.91 | 1.19 | 6 | | 2 | SM |
| 0.16 | 1.43 | 1.19 | 6 | | 2 | SM |
| 0.10 | 2.40 | 1.20 | 6 | | 2 | SM |
| 0.94 | 1.11 | 1.17 | 6 | | 2 | SM |
| -0.95 | 1.04 | 1.23 | 6 | | 2 | SM |
| 0.34 | 1.18 | 1.19 | 6 | | 2 | SM |
| 0.28 | 1.86 | 1.19 | 6 | | 2 | SM |
| 0.25 | 2.04 | 1.19 | 6 | | 2 | SM |
| -0.54 | 0.85 | 1.22 | 6 | | 2 | SM |
| 0.35 | 0.60 | 1.19 | 6 | | 2 | SM |
| -0.35 | 2.03 | 1.21 | 6 | | 2 | SM |
| 1.34 | 1.43 | 1.15 | 6 | | 2 | SM |
| -0.63 | 1.00 | 1.22 | 6 | | 2 | SM |
| 0.52 | 0.30 | 1.18 | 6 | | 2 | SM |
| -0.89 | 1.15 | 1.23 | 6 | | 2 | SM |
| 0.30 | 1.52 | 1.19 | 6 | | 2 | SM |
| 0.61 | 1.04 | 1.18 | 6 | | 2 | SM |
| -0.20 | 1.46 | 1.21 | 6 | | 2 | SM |
| 0.05 | 0.30 | 1.20 | 6 | | 2 | SM |
| 0.52 | 1.40 | 1.18 | 6 | | 2 | SM |
| -0.13 | 0.00 | 1.20 | 6 | | 2 | SM |
| 0.09 | 1.65 | 1.20 | 6 | | 2 | SM |
| 0.12 | 0.30 | 1.20 | 6 | | 2 | SM |
| 0.34 | 1.26 | 1.19 | 6 | | 2 | SM |
| -0.84 | 0.70 | 1.23 | 6 | | 2 | SM |
| -0.90 | 1.54 | 1.23 | 6 | | 2 | SM |
| 1.18 | 0.00 | 1.16 | 6 | | 2 | SM |
| 0.08 | 1.72 | 1.20 | 6 | | 2 | SM |
| -0.15 | 0.00 | 1.21 | 6 | | 2 | SM |
| -0.63 | 1.81 | 1.22 | 6 | 2 | | SM |
| -0.11 | 2.47 | 1.60 | 10 | 2 | | SM |
| 0.09 | 1.81 | 1.66 | 10 | 2 | | SM |
| 0.26 | 2.14 | 1.71 | 10 | 2 | | SM |
| -0.05 | 2.73 | 1.62 | 10 | | 2 | SM |
| -0.41 | 0.70 | 1.52 | 10 | | 2 | SM |
| -0.35 | 0.70 | 1.54 | 10 | | 2 | SM |
| 0.53 | 1.88 | 1.78 | 10 | | 2 | SM |
| 0.72 | 2.79 | 1.83 | 10 | | 2 | SM |
| 0.09 | 1.81 | 1.66 | 10 | | 2 | SM |
| 0.17 | 2.76 | 1.68 | 10 | | 2 | SM |
| 0.46 | 1.90 | 1.76 | 10 | | 2 | SM |
| -0.29 | 1.00 | 1.56 | 10 | | 2 | SM |
| -0.28 | 1.28 | 1.56 | 10 | | 2 | SM |
| -0.22 | 2.55 | 1.57 | 10 | | 2 | SM |
| -0.04 | 0.00 | 1.63 | 10 | | 2 | SM |
| -0.29 | 0.48 | 1.56 | 10 | | 2 | SM |
| -0.23 | 1.97 | 1.57 | 10 | | 2 | SM |
| -0.48 | 2.45 | 1.50 | 10 | | 2 | SM |
| -1.11 | 0.00 | 1.33 | 10 | | 2 | SM |
| -0.53 | 2.27 | 1.49 | 10 | | 2 | SM |
| 0.06 | 2.33 | 1.65 | 10 | | 2 | SM |
| 0.08 | 2.27 | 1.66 | 10 | | 2 | SM |
| -4.05 | 0.30 | 0.53 | 10 | | 2 | SM |
| -0.77 | 0.00 | 1.43 | 10 | | 2 | SM |
| -0.09 | 2.47 | 1.61 | 10 | | 2 | SM |
| 0.30 | 2.72 | 1.72 | 10 | | 2 | SM |
| 0.20 | 1.83 | 1.69 | 10 | | 2 | SM |
| 0.41 | 2.63 | 1.75 | 10 | | 2 | SM |
| 0.62 | 2.13 | 1.81 | 10 | | 2 | SM |
| -0.19 | 0.90 | 1.58 | 10 | | 2 | SM |
| -1.07 | 2.48 | 1.34 | 10 | | 2 | SM |
| 0.34 | 2.57 | 1.73 | 10 | | 2 | SM |
| 0.16 | 1.26 | 1.68 | 10 | | 2 | SM |
| 0.10 | 2.23 | 1.66 | 10 | | 2 | SM |
| 0.94 | 1.28 | 1.89 | 10 | | 2 | SM |
| -0.95 | 1.83 | 1.37 | 10 | | 2 | SM |
| 0.34 | 2.87 | 1.73 | 10 | | 2 | SM |
| 0.28 | 1.20 | 1.71 | 10 | | 2 | SM |
| -0.76 | 0.30 | 1.43 | 10 | | 2 | SM |
| 0.25 | 2.51 | 1.70 | 10 | | 2 | SM |
| -0.54 | 2.00 | 1.49 | 10 | | 2 | SM |
| 0.35 | 0.60 | 1.73 | 10 | | 2 | SM |
| -0.35 | 2.09 | 1.54 | 10 | | 2 | SM |
| -0.16 | 0.00 | 1.59 | 10 | | 2 | SM |
| 1.34 | 2.31 | 2.00 | 10 | | 2 | SM |
| -0.63 | 1.11 | 1.46 | 10 | | 2 | SM |
| -0.62 | 0.48 | 1.47 | 10 | | 2 | SM |
| 0.52 | 1.18 | 1.78 | 10 | | 2 | SM |
| -0.89 | 2.25 | 1.39 | 10 | | 2 | SM |
| 0.30 | 0.78 | 1.72 | 10 | | 2 | SM |
| 0.61 | 2.02 | 1.80 | 10 | | 2 | SM |
| -0.20 | 2.21 | 1.58 | 10 | | 2 | SM |
| 0.05 | 2.38 | 1.65 | 10 | | 2 | SM |
| 0.52 | 1.04 | 1.78 | 10 | | 2 | SM |
| -0.55 | 1.71 | 1.49 | 10 | | 2 | SM |
| -0.90 | 2.25 | 1.39 | 10 | | 2 | SM |
| -0.13 | 1.83 | 1.60 | 10 | | 2 | SM |
| 0.09 | 2.37 | 1.66 | 10 | | 2 | SM |
| 0.12 | 0.00 | 1.67 | 10 | | 2 | SM |
| 0.17 | 0.78 | 1.68 | 10 | | 2 | SM |
| 0.34 | 0.85 | 1.73 | 10 | | 2 | SM |
| -0.84 | 2.67 | 1.40 | 10 | | 2 | SM |
| -0.90 | 1.91 | 1.39 | 10 | | 2 | SM |
| 0.86 | 1.49 | 1.87 | 10 | | 2 | SM |
| -0.15 | 0.48 | 1.59 | 10 | | 2 | SM |
| 0.55 | 0.00 | 1.79 | 10 | | 2 | SM |
| -0.63 | 0.95 | 1.46 | 10 | | 2 | SM |
| -0.11 | 2.85 | 1.63 | 13 | | 2 | SM |
| -0.67 | 0.30 | 1.57 | 13 | | 2 | SM |
| 0.09 | 2.06 | 1.65 | 13 | | 2 | SM |
| -0.24 | 0.00 | 1.61 | 13 | | 2 | SM |
| 0.26 | 1.32 | 1.67 | 13 | | 2 | SM |
| -0.05 | 2.69 | 1.63 | 13 | | 2 | SM |
| -0.41 | 1.11 | 1.60 | 13 | | 2 | SM |
| -0.35 | 0.60 | 1.60 | 13 | | 2 | SM |
| 0.53 | 1.86 | 1.70 | 13 | | 2 | SM |
| 0.72 | 2.19 | 1.72 | 13 | | 2 | SM |
| 0.09 | 2.76 | 1.65 | 13 | | 2 | SM |
| 0.17 | 2.56 | 1.66 | 13 | | 2 | SM |
| 0.46 | 2.11 | 1.69 | 13 | | 2 | SM |
| -0.29 | 1.40 | 1.61 | 13 | | 2 | SM |
| 0.21 | 0.95 | 1.66 | 13 | | 2 | SM |
| -0.28 | 0.85 | 1.61 | 13 | | 2 | SM |
| -0.22 | 2.03 | 1.62 | 13 | | 2 | SM |
| -0.29 | 0.90 | 1.61 | 13 | | 2 | SM |
| -0.26 | 0.78 | 1.61 | 13 | | 2 | SM |
| -0.48 | 1.89 | 1.59 | 13 | | 2 | SM |
| 0.13 | 2.14 | 1.65 | 13 | | 2 | SM |
| -1.11 | 2.30 | 1.52 | 13 | | 2 | SM |
| -0.53 | 1.66 | 1.58 | 13 | | 2 | SM |
| 0.06 | 2.58 | 1.65 | 13 | | 2 | SM |
| 0.08 | 0.00 | 1.65 | 13 | | 2 | SM |
| -4.05 | 1.90 | 1.20 | 13 | | 2 | SM |
| -0.77 | 0.60 | 1.56 | 13 | | 2 | SM |
| 0.30 | 2.81 | 1.67 | 13 | | 2 | SM |
| 0.20 | 2.29 | 1.66 | 13 | | 2 | SM |
| 0.41 | 2.55 | 1.68 | 13 | | 2 | SM |
| -0.19 | 0.70 | 1.62 | 13 | | 2 | SM |
| -1.07 | 2.53 | 1.52 | 13 | | 2 | SM |
| 0.34 | 2.15 | 1.68 | 13 | | 2 | SM |
| 0.16 | 1.40 | 1.66 | 13 | | 2 | SM |
| 0.10 | 2.35 | 1.65 | 13 | | 2 | SM |
| -0.95 | 2.68 | 1.54 | 13 | | 2 | SM |
| 0.34 | 1.76 | 1.68 | 13 | | 2 | SM |
| 0.28 | 1.88 | 1.67 | 13 | | 2 | SM |
| -0.76 | 0.00 | 1.56 | 13 | | 2 | SM |
| 0.25 | 2.77 | 1.67 | 13 | | 2 | SM |
| -0.54 | 2.27 | 1.58 | 13 | | 2 | SM |
| -0.16 | 1.43 | 1.62 | 13 | | 2 | SM |
| 0.35 | 1.86 | 1.68 | 13 | | 2 | SM |
| -0.35 | 1.80 | 1.60 | 13 | | 2 | SM |
| -0.16 | 0.70 | 1.62 | 13 | | 2 | SM |
| 1.34 | 2.59 | 1.78 | 13 | | 2 | SM |
| -0.63 | 1.11 | 1.57 | 13 | | 2 | SM |
| -0.62 | 1.04 | 1.57 | 13 | | 2 | SM |
| 0.52 | 1.30 | 1.70 | 13 | | 2 | SM |
| -0.89 | 1.84 | 1.54 | 13 | | 2 | SM |
| 0.30 | 1.89 | 1.67 | 13 | | 2 | SM |
| 0.51 | 1.26 | 1.69 | 13 | | 2 | SM |
| 0.61 | 2.93 | 1.71 | 13 | | 2 | SM |
| -0.20 | 1.96 | 1.62 | 13 | | 2 | SM |
| 0.05 | 1.91 | 1.65 | 13 | | 2 | SM |
| 0.52 | 1.77 | 1.70 | 13 | | 2 | SM |
| -0.55 | 0.60 | 1.58 | 13 | | 2 | SM |
| -0.90 | 1.61 | 1.54 | 13 | | 2 | SM |
| -0.13 | 1.61 | 1.63 | 13 | | 2 | SM |
| 0.09 | 1.93 | 1.65 | 13 | | 2 | SM |
| 0.17 | 1.45 | 1.66 | 13 | | 2 | SM |
| 0.34 | 1.43 | 1.68 | 13 | | 2 | SM |
| -0.84 | 2.79 | 1.55 | 13 | | 2 | SM |
| -0.90 | 2.16 | 1.54 | 13 | | 2 | SM |
| 0.86 | 1.45 | 1.73 | 13 | | 2 | SM |
| -0.15 | 1.15 | 1.62 | 13 | | 2 | SM |
| 0.55 | 1.18 | 1.70 | 13 | | 2 | SM |
| -0.63 | 1.65 | 1.57 | 13 | | 2 | SM |
| 0.51 | 0.00 | 1.69 | 13 | | 2 | SM |
| -1.04 | 0.48 | 1.53 | 13 | | 2 | SM |
| 0.25 | 0.00 | 1.67 | 13 | | 2 | SM |
| -0.11 | 2.52 | 1.57 | undisturbed | | 2 | SM |
| -0.30 | 1.26 | 1.56 | undisturbed | | 2 | SM |
| 0.09 | 2.31 | 1.58 | undisturbed | | 2 | SM |
| -0.24 | 1.89 | 1.56 | undisturbed | | 2 | SM |
| -1.04 | 0.00 | 1.51 | undisturbed | | 2 | SM |
| 0.26 | 1.46 | 1.59 | undisturbed | | 2 | SM |
| -0.19 | 1.04 | 1.56 | undisturbed | | 2 | SM |
| -0.05 | 2.62 | 1.57 | undisturbed | | 2 | SM |
| -0.41 | 0.70 | 1.55 | undisturbed | | 2 | SM |
| -0.46 | 0.95 | 1.55 | undisturbed | | 2 | SM |
| -0.15 | 1.36 | 1.56 | undisturbed | | 2 | SM |
| 0.53 | 1.42 | 1.61 | undisturbed | | 2 | SM |
| 0.72 | 1.83 | 1.62 | undisturbed | | 2 | SM |
| 0.07 | 1.04 | 1.58 | undisturbed | | 2 | SM |
| 0.09 | 2.64 | 1.58 | undisturbed | | 2 | SM |
| 0.17 | 2.63 | 1.58 | undisturbed | | 2 | SM |
| 0.46 | 2.72 | 1.60 | undisturbed | | 2 | SM |
| -0.29 | 0.30 | 1.56 | undisturbed | | 2 | SM |
| 0.21 | 1.08 | 1.59 | undisturbed | | 2 | SM |
| -0.28 | 1.20 | 1.56 | undisturbed | | 2 | SM |
| -0.22 | 1.75 | 1.56 | undisturbed | | 2 | SM |
| -0.29 | 2.15 | 1.56 | undisturbed | | 2 | SM |
| -0.23 | 0.00 | 1.56 | undisturbed | | 2 | SM |
| -0.48 | 1.94 | 1.54 | undisturbed | | 2 | SM |
| 0.13 | 2.18 | 1.58 | undisturbed | | 2 | SM |
| -0.11 | 2.77 | 1.57 | undisturbed | | 2 | SM |
| -0.53 | 1.18 | 1.54 | undisturbed | | 2 | SM |
| 0.06 | 2.51 | 1.58 | undisturbed | | 2 | SM |
| 0.08 | 2.20 | 1.58 | undisturbed | | 2 | SM |
| -4.05 | 2.54 | 1.33 | undisturbed | | 2 | SM |
| -0.09 | 2.01 | 1.57 | undisturbed | | 2 | SM |
| 0.30 | 2.55 | 1.59 | undisturbed | | 2 | SM |
| 0.20 | 2.26 | 1.59 | undisturbed | | 2 | SM |
| 0.41 | 2.49 | 1.60 | undisturbed | | 2 | SM |
| -0.11 | 0.00 | 1.57 | undisturbed | | 2 | SM |
| -1.07 | 2.37 | 1.51 | undisturbed | | 2 | SM |
| 0.34 | 1.59 | 1.60 | undisturbed | | 2 | SM |
| 0.10 | 2.12 | 1.58 | undisturbed | | 2 | SM |
| 0.94 | 0.30 | 1.63 | undisturbed | | 2 | SM |
| -0.95 | 1.99 | 1.52 | undisturbed | | 2 | SM |
| 0.34 | 1.86 | 1.59 | undisturbed | | 2 | SM |
| 0.28 | 1.88 | 1.59 | undisturbed | | 2 | SM |
| -0.76 | 0.00 | 1.53 | undisturbed | | 2 | SM |
| 0.25 | 2.74 | 1.59 | undisturbed | | 2 | SM |
| -0.54 | 1.61 | 1.54 | undisturbed | | 2 | SM |
| 0.35 | 1.42 | 1.60 | undisturbed | | 2 | SM |
| -0.35 | 1.60 | 1.55 | undisturbed | | 2 | SM |
| 1.34 | 1.81 | 1.66 | undisturbed | | 2 | SM |
| -0.63 | 0.30 | 1.54 | undisturbed | | 2 | SM |
| 0.16 | 0.48 | 1.58 | undisturbed | | 2 | SM |
| -0.62 | 0.00 | 1.54 | undisturbed | | 2 | SM |
| 0.52 | 0.78 | 1.61 | undisturbed | | 2 | SM |
| -0.89 | 2.18 | 1.52 | undisturbed | | 2 | SM |
| 0.30 | 2.00 | 1.59 | undisturbed | | 2 | SM |
| 0.61 | 2.93 | 1.61 | undisturbed | | 2 | SM |
| -0.20 | 2.10 | 1.56 | undisturbed | | 2 | SM |
| 0.05 | 1.00 | 1.58 | undisturbed | | 2 | SM |
| 0.52 | 1.18 | 1.61 | undisturbed | | 2 | SM |
| -0.55 | 0.00 | 1.54 | undisturbed | | 2 | SM |
| 0.25 | 0.30 | 1.59 | undisturbed | | 2 | SM |
| -0.90 | 1.34 | 1.52 | undisturbed | | 2 | SM |
| -0.13 | 2.62 | 1.57 | undisturbed | | 2 | SM |
| 0.09 | 1.92 | 1.58 | undisturbed | | 2 | SM |
| 0.12 | 0.00 | 1.58 | undisturbed | | 2 | SM |
| 0.17 | 2.20 | 1.58 | undisturbed | | 2 | SM |
| -0.84 | 1.95 | 1.52 | undisturbed | | 2 | SM |
| -0.90 | 2.14 | 1.52 | undisturbed | | 2 | SM |
| -0.15 | 1.20 | 1.56 | undisturbed | | 2 | SM |
| 0.26 | 2.27 | 1.59 | undisturbed | | 2 | SM |
| -0.26 | 0.00 | 1.56 | undisturbed | | 2 | SM |
| -1.48 | 1.92 | 1.42 | 4 | | 1 | SG |
| 0.00 | 1.52 | 0.91 | 4 | | 1 | SG |
| -0.80 | 2.40 | 1.18 | 4 | | 1 | SG |
| -0.01 | 1.92 | 0.91 | 4 | | 1 | SG |
| -0.12 | 0.85 | 0.95 | 4 | | 1 | SG |
| -0.23 | 0.00 | 0.99 | 4 | | 1 | SG |
| -0.30 | 0.00 | 1.01 | 4 | | 1 | SG |
| -1.57 | 2.29 | 1.45 | 4 | | 1 | SG |
| -0.17 | 1.65 | 0.97 | 4 | | 1 | SG |
| -0.27 | 0.00 | 1.00 | 4 | | 1 | SG |
| -1.57 | 0.30 | 1.45 | 4 | | 1 | SG |
| -0.62 | 1.69 | 1.12 | 4 | | 1 | SG |
| -0.76 | 0.78 | 1.17 | 4 | | 1 | SG |
| -0.17 | 0.30 | 0.97 | 4 | | 1 | SG |
| -0.68 | 0.48 | 1.14 | 4 | | 1 | SG |
| -0.24 | 1.46 | 0.99 | 4 | | 1 | SG |
| -0.26 | 1.79 | 1.00 | 4 | | 1 | SG |
| -2.60 | 2.57 | 1.80 | 4 | | 1 | SG |
| -1.00 | 0.30 | 1.25 | 4 | | 1 | SG |
| -0.88 | 1.79 | 1.21 | 4 | | 1 | SG |
| -0.44 | 0.00 | 1.06 | 4 | | 1 | SG |
| -1.57 | 2.39 | 1.45 | 4 | | 1 | SG |
| -0.31 | 0.30 | 1.01 | 4 | | 1 | SG |
| -2.18 | 0.85 | 1.66 | 4 | | 1 | SG |
| -0.95 | 3.10 | 1.23 | 4 | | 1 | SG |
| -0.71 | 1.90 | 1.15 | 4 | | 1 | SG |
| -1.18 | 0.48 | 1.31 | 4 | | 1 | SG |
| -0.76 | 0.48 | 1.17 | 4 | | 1 | SG |
| -0.88 | 0.00 | 1.21 | 4 | | 1 | SG |
| -1.03 | 1.95 | 1.26 | 4 | | 1 | SG |
| -0.80 | 0.48 | 1.18 | 4 | | 1 | SG |
| -0.32 | 0.30 | 1.02 | 4 | | 1 | SG |
| -0.01 | 0.00 | 0.91 | 4 | | 1 | SG |
| -1.33 | 1.96 | 1.37 | 4 | | 1 | SG |
| -0.73 | 0.70 | 1.16 | 4 | | 1 | SG |
| -0.05 | 1.26 | 0.93 | 4 | | 1 | SG |
| -0.38 | 0.00 | 1.04 | 4 | | 1 | SG |
| -0.03 | 1.08 | 0.92 | 4 | | 1 | SG |
| -0.05 | 1.54 | 0.93 | 4 | | 1 | SG |
| -0.17 | 1.32 | 0.97 | 4 | | 1 | SG |
| -1.70 | 0.30 | 1.49 | 4 | | 1 | SG |
| -0.34 | 2.89 | 1.03 | 4 | | 1 | SG |
| -0.59 | 2.16 | 1.11 | 4 | | 1 | SG |
| -0.52 | 1.18 | 1.09 | 4 | | 1 | SG |
| -0.13 | 1.89 | 0.96 | 4 | | 1 | SG |
| -0.40 | 1.69 | 1.05 | 4 | | 1 | SG |
| -0.01 | 0.30 | 0.91 | 4 | | 1 | SG |
| -0.36 | 0.00 | 1.03 | 4 | | 1 | SG |
| -1.33 | 2.22 | 1.59 | 6 | | 1 | SG |
| -0.64 | 1.86 | 1.49 | 6 | | 1 | SG |
| -1.48 | 2.18 | 1.61 | 6 | | 1 | SG |
| 0.00 | 2.05 | 1.39 | 6 | | 1 | SG |
| -0.80 | 2.04 | 1.51 | 6 | | 1 | SG |
| -0.12 | 2.62 | 1.41 | 6 | | 1 | SG |
| -0.23 | 0.90 | 1.42 | 6 | | 1 | SG |
| -0.04 | 1.00 | 1.40 | 6 | | 1 | SG |
| -1.57 | 2.59 | 1.63 | 6 | | 1 | SG |
| -0.17 | 0.60 | 1.42 | 6 | | 1 | SG |
| -0.27 | 0.48 | 1.43 | 6 | | 1 | SG |
| -1.57 | 0.30 | 1.63 | 6 | | 1 | SG |
| -0.62 | 1.88 | 1.48 | 6 | | 1 | SG |
| -0.76 | 1.84 | 1.51 | 6 | | 1 | SG |
| -0.17 | 0.70 | 1.42 | 6 | | 1 | SG |
| -0.68 | 1.68 | 1.49 | 6 | | 1 | SG |
| -0.24 | 1.04 | 1.43 | 6 | | 1 | SG |
| -0.26 | 1.40 | 1.43 | 6 | | 1 | SG |
| -2.30 | 1.70 | 1.74 | 6 | | 1 | SG |
| -1.00 | 1.04 | 1.54 | 6 | | 1 | SG |
| -0.88 | 0.00 | 1.52 | 6 | | 1 | SG |
| -1.57 | 2.26 | 1.63 | 6 | | 1 | SG |
| -0.31 | 3.00 | 1.44 | 6 | | 1 | SG |
| -2.18 | 1.23 | 1.72 | 6 | | 1 | SG |
| -0.95 | 3.01 | 1.53 | 6 | | 1 | SG |
| -0.71 | 1.95 | 1.50 | 6 | | 1 | SG |
| -0.76 | 1.23 | 1.51 | 6 | | 1 | SG |
| -0.88 | 2.02 | 1.52 | 6 | | 1 | SG |
| -1.03 | 1.99 | 1.55 | 6 | | 1 | SG |
| -0.80 | 1.54 | 1.51 | 6 | | 1 | SG |
| -0.32 | 0.00 | 1.44 | 6 | | 1 | SG |
| -0.01 | 2.05 | 1.39 | 6 | | 1 | SG |
| -1.33 | 2.02 | 1.59 | 6 | | 1 | SG |
| -0.73 | 1.08 | 1.50 | 6 | | 1 | SG |
| -0.05 | 1.89 | 1.40 | 6 | | 1 | SG |
| -0.38 | 1.20 | 1.45 | 6 | | 1 | SG |
| -0.03 | 1.18 | 1.39 | 6 | | 1 | SG |
| -0.03 | 1.30 | 1.39 | 6 | | 1 | SG |
| -0.05 | 1.54 | 1.40 | 6 | | 1 | SG |
| -0.17 | 1.38 | 1.42 | 6 | | 1 | SG |
| -1.70 | 1.71 | 1.65 | 6 | | 1 | SG |
| -2.30 | 0.78 | 1.74 | 6 | | 1 | SG |
| -0.14 | 1.00 | 1.41 | 6 | | 1 | SG |
| -0.12 | 1.04 | 1.41 | 6 | | 1 | SG |
| -0.34 | 2.32 | 1.44 | 6 | | 1 | SG |
| -0.59 | 1.53 | 1.48 | 6 | | 1 | SG |
| -0.52 | 1.04 | 1.47 | 6 | | 1 | SG |
| -0.13 | 1.38 | 1.41 | 6 | | 1 | SG |
| -0.40 | 0.70 | 1.45 | 6 | | 1 | SG |
| -0.01 | 1.11 | 1.39 | 6 | | 1 | SG |
| -1.33 | 1.20 | 1.09 | 10 | | 1 | SG |
| -0.64 | 1.34 | 1.49 | 10 | | 1 | SG |
| -1.33 | 0.90 | 1.09 | 10 | | 1 | SG |
| -2.30 | 0.00 | 0.53 | 10 | | 1 | SG |
| -1.13 | 0.00 | 1.21 | 10 | | 1 | SG |
| -2.30 | 0.70 | 0.53 | 10 | | 1 | SG |
| -0.02 | 2.16 | 1.85 | 10 | | 1 | SG |
| -1.48 | 0.48 | 1.01 | 10 | | 1 | SG |
| -0.80 | 1.20 | 1.40 | 10 | | 1 | SG |
| -0.01 | 1.32 | 1.86 | 10 | | 1 | SG |
| -0.12 | 1.80 | 1.80 | 10 | | 1 | SG |
| -0.23 | 2.16 | 1.73 | 10 | | 1 | SG |
| -0.30 | 1.36 | 1.69 | 10 | | 1 | SG |
| -1.57 | 2.50 | 0.95 | 10 | | 1 | SG |
| -1.57 | 0.60 | 0.95 | 10 | | 1 | SG |
| -1.40 | 0.00 | 1.05 | 10 | | 1 | SG |
| -0.07 | 2.10 | 1.82 | 10 | | 1 | SG |
| -0.76 | 1.04 | 1.42 | 10 | | 1 | SG |
| -0.68 | 1.94 | 1.47 | 10 | | 1 | SG |
| -0.24 | 1.58 | 1.72 | 10 | | 1 | SG |
| -0.26 | 2.34 | 1.72 | 10 | | 1 | SG |
| -1.00 | 1.18 | 1.28 | 10 | | 1 | SG |
| -1.57 | 0.60 | 0.95 | 10 | | 1 | SG |
| -0.31 | 2.96 | 1.69 | 10 | | 1 | SG |
| -0.95 | 1.46 | 1.32 | 10 | | 1 | SG |
| -0.28 | 1.51 | 1.70 | 10 | | 1 | SG |
| -0.76 | 1.98 | 1.42 | 10 | | 1 | SG |
| -0.88 | 1.08 | 1.36 | 10 | | 1 | SG |
| -1.03 | 1.08 | 1.27 | 10 | | 1 | SG |
| -0.32 | 1.36 | 1.68 | 10 | | 1 | SG |
| -0.01 | 1.87 | 1.86 | 10 | | 1 | SG |
| -0.64 | 1.52 | 1.49 | 10 | | 1 | SG |
| -1.33 | 0.60 | 1.09 | 10 | | 1 | SG |
| -0.73 | 0.90 | 1.44 | 10 | | 1 | SG |
| -0.05 | 2.22 | 1.83 | 10 | | 1 | SG |
| -0.38 | 2.52 | 1.64 | 10 | | 1 | SG |
| -0.03 | 1.56 | 1.85 | 10 | | 1 | SG |
| -0.03 | 1.42 | 1.85 | 10 | | 1 | SG |
| -0.05 | 1.68 | 1.84 | 10 | | 1 | SG |
| -0.17 | 1.97 | 1.77 | 10 | | 1 | SG |
| -1.70 | 2.89 | 0.88 | 10 | | 1 | SG |
| -0.14 | 1.84 | 1.78 | 10 | | 1 | SG |
| -0.23 | 1.26 | 1.73 | 10 | | 1 | SG |
| -0.12 | 1.30 | 1.80 | 10 | | 1 | SG |
| -0.34 | 1.36 | 1.67 | 10 | | 1 | SG |
| -0.59 | 1.60 | 1.53 | 10 | | 1 | SG |
| -0.52 | 2.47 | 1.56 | 10 | | 1 | SG |
| -0.13 | 1.79 | 1.79 | 10 | | 1 | SG |
| -0.01 | 1.52 | 1.86 | 10 | | 1 | SG |
| -0.36 | 1.71 | 1.65 | 10 | | 1 | SG |
| -1.33 | 0.48 | 1.11 | 13 | | 1 | SG |
| -0.64 | 0.48 | 1.36 | 13 | | 1 | SG |
| -0.95 | 1.18 | 1.25 | 13 | | 1 | SG |
| -1.33 | 1.62 | 1.11 | 13 | | 1 | SG |
| -2.30 | 0.00 | 0.75 | 13 | | 1 | SG |
| -1.13 | 0.48 | 1.18 | 13 | | 1 | SG |
| -0.04 | 1.08 | 1.59 | 13 | | 1 | SG |
| -0.02 | 1.96 | 1.60 | 13 | | 1 | SG |
| -1.48 | 0.48 | 1.05 | 13 | | 1 | SG |
| -0.80 | 0.70 | 1.31 | 13 | | 1 | SG |
| -0.01 | 1.40 | 1.60 | 13 | | 1 | SG |
| -0.12 | 1.94 | 1.56 | 13 | | 1 | SG |
| -0.23 | 1.72 | 1.52 | 13 | | 1 | SG |
| -0.30 | 2.81 | 1.49 | 13 | | 1 | SG |
| -1.57 | 0.30 | 1.02 | 13 | | 1 | SG |
| -0.17 | 1.99 | 1.54 | 13 | | 1 | SG |
| -0.27 | 0.95 | 1.50 | 13 | | 1 | SG |
| -0.07 | 0.60 | 1.58 | 13 | | 1 | SG |
| -0.07 | 2.14 | 1.58 | 13 | | 1 | SG |
| -0.47 | 2.30 | 1.43 | 13 | | 1 | SG |
| -0.76 | 1.20 | 1.32 | 13 | | 1 | SG |
| -0.68 | 1.54 | 1.35 | 13 | | 1 | SG |
| -0.24 | 2.42 | 1.51 | 13 | | 1 | SG |
| -0.26 | 1.87 | 1.51 | 13 | | 1 | SG |
| -2.30 | 0.00 | 0.75 | 13 | | 1 | SG |
| -1.00 | 1.11 | 1.23 | 13 | | 1 | SG |
| -2.30 | 0.70 | 0.75 | 13 | | 1 | SG |
| -0.31 | 2.78 | 1.49 | 13 | | 1 | SG |
| -2.18 | 2.06 | 0.79 | 13 | | 1 | SG |
| -0.95 | 2.41 | 1.25 | 13 | | 1 | SG |
| -0.28 | 0.70 | 1.50 | 13 | | 1 | SG |
| -0.76 | 2.38 | 1.32 | 13 | | 1 | SG |
| -0.88 | 1.98 | 1.28 | 13 | | 1 | SG |
| -1.03 | 0.30 | 1.22 | 13 | | 1 | SG |
| -0.80 | 0.60 | 1.31 | 13 | | 1 | SG |
| -0.32 | 1.67 | 1.48 | 13 | | 1 | SG |
| -0.01 | 1.72 | 1.60 | 13 | | 1 | SG |
| -0.64 | 0.78 | 1.36 | 13 | | 1 | SG |
| -1.33 | 2.47 | 1.11 | 13 | | 1 | SG |
| -0.73 | 0.48 | 1.33 | 13 | | 1 | SG |
| -0.05 | 0.30 | 1.59 | 13 | | 1 | SG |
| -0.38 | 2.55 | 1.46 | 13 | | 1 | SG |
| -1.18 | 0.00 | 1.17 | 13 | | 1 | SG |
| -0.03 | 1.00 | 1.59 | 13 | | 1 | SG |
| -0.03 | 1.70 | 1.59 | 13 | | 1 | SG |
| -0.05 | 1.85 | 1.59 | 13 | | 1 | SG |
| -0.17 | 2.95 | 1.54 | 13 | | 1 | SG |
| -1.70 | 1.30 | 0.97 | 13 | | 1 | SG |
| -2.30 | 1.74 | 0.75 | 13 | | 1 | SG |
| -0.14 | 0.60 | 1.55 | 13 | | 1 | SG |
| -0.23 | 1.11 | 1.52 | 13 | | 1 | SG |
| -0.11 | 0.70 | 1.56 | 13 | | 1 | SG |
| -0.12 | 2.11 | 1.56 | 13 | | 1 | SG |
| -0.34 | 1.91 | 1.48 | 13 | | 1 | SG |
| -0.32 | 1.30 | 1.48 | 13 | | 1 | SG |
| -0.59 | 1.48 | 1.39 | 13 | | 1 | SG |
| -0.52 | 2.20 | 1.41 | 13 | | 1 | SG |
| -0.13 | 1.48 | 1.55 | 13 | | 1 | SG |
| -0.51 | 0.60 | 1.41 | 13 | | 1 | SG |
| -0.01 | 1.04 | 1.60 | 13 | | 1 | SG |
| -0.36 | 1.15 | 1.47 | 13 | | 1 | SG |
| -1.33 | 0.48 | 1.37 | undisturbed | | 1 | SG |
| -1.03 | 1.11 | 1.40 | undisturbed | | 1 | SG |
| -1.33 | 0.30 | 1.37 | undisturbed | | 1 | SG |
| -2.30 | 0.00 | 1.27 | undisturbed | | 1 | SG |
| -1.13 | 0.00 | 1.39 | undisturbed | | 1 | SG |
| -2.30 | 0.78 | 1.27 | undisturbed | | 1 | SG |
| -0.04 | 1.67 | 1.50 | undisturbed | | 1 | SG |
| -0.02 | 2.49 | 1.50 | undisturbed | | 1 | SG |
| -1.48 | 0.00 | 1.36 | undisturbed | | 1 | SG |
| -0.01 | 1.78 | 1.50 | undisturbed | | 1 | SG |
| -0.12 | 1.43 | 1.49 | undisturbed | | 1 | SG |
| -0.07 | 1.04 | 1.50 | undisturbed | | 1 | SG |
| -0.30 | 2.82 | 1.48 | undisturbed | | 1 | SG |
| -1.57 | 2.49 | 1.35 | undisturbed | | 1 | SG |
| -0.17 | 1.42 | 1.49 | undisturbed | | 1 | SG |
| -0.27 | 0.48 | 1.48 | undisturbed | | 1 | SG |
| -0.07 | 1.77 | 1.50 | undisturbed | | 1 | SG |
| -0.07 | 0.00 | 1.50 | undisturbed | | 1 | SG |
| -0.76 | 1.71 | 1.43 | undisturbed | | 1 | SG |
| -0.68 | 1.46 | 1.44 | undisturbed | | 1 | SG |
| -0.24 | 2.49 | 1.48 | undisturbed | | 1 | SG |
| -0.26 | 1.11 | 1.48 | undisturbed | | 1 | SG |
| -2.30 | 2.49 | 1.27 | undisturbed | | 1 | SG |
| -1.00 | 1.81 | 1.41 | undisturbed | | 1 | SG |
| -2.30 | 2.49 | 1.27 | undisturbed | | 1 | SG |
| -0.31 | 2.42 | 1.47 | undisturbed | | 1 | SG |
| -2.18 | 2.21 | 1.29 | undisturbed | | 1 | SG |
| -0.95 | 2.32 | 1.41 | undisturbed | | 1 | SG |
| -0.71 | 0.30 | 1.43 | undisturbed | | 1 | SG |
| -0.28 | 1.93 | 1.48 | undisturbed | | 1 | SG |
| -1.18 | 0.00 | 1.39 | undisturbed | | 1 | SG |
| -0.76 | 2.30 | 1.43 | undisturbed | | 1 | SG |
| -0.88 | 1.61 | 1.42 | undisturbed | | 1 | SG |
| -0.80 | 1.00 | 1.43 | undisturbed | | 1 | SG |
| -0.32 | 1.59 | 1.47 | undisturbed | | 1 | SG |
| -0.01 | 1.95 | 1.50 | undisturbed | | 1 | SG |
| -0.64 | 0.48 | 1.44 | undisturbed | | 1 | SG |
| -1.33 | 2.70 | 1.37 | undisturbed | | 1 | SG |
| -0.73 | 1.88 | 1.43 | undisturbed | | 1 | SG |
| -0.05 | 1.18 | 1.50 | undisturbed | | 1 | SG |
| -0.05 | 0.78 | 1.50 | undisturbed | | 1 | SG |
| -0.38 | 1.79 | 1.47 | undisturbed | | 1 | SG |
| -1.18 | 0.30 | 1.39 | undisturbed | | 1 | SG |
| -0.03 | 0.30 | 1.50 | undisturbed | | 1 | SG |
| -0.03 | 1.74 | 1.50 | undisturbed | | 1 | SG |
| -0.05 | 2.16 | 1.50 | undisturbed | | 1 | SG |
| -0.17 | 3.00 | 1.49 | undisturbed | | 1 | SG |
| -1.70 | 1.75 | 1.34 | undisturbed | | 1 | SG |
| -2.30 | 1.42 | 1.27 | undisturbed | | 1 | SG |
| -0.14 | 1.04 | 1.49 | undisturbed | | 1 | SG |
| -0.23 | 0.00 | 1.48 | undisturbed | | 1 | SG |
| -0.11 | 1.30 | 1.49 | undisturbed | | 1 | SG |
| -0.12 | 2.59 | 1.49 | undisturbed | | 1 | SG |
| -0.34 | 2.19 | 1.47 | undisturbed | | 1 | SG |
| -0.32 | 1.54 | 1.47 | undisturbed | | 1 | SG |
| -0.59 | 0.00 | 1.45 | undisturbed | | 1 | SG |
| -0.52 | 2.08 | 1.45 | undisturbed | | 1 | SG |
| -0.13 | 2.36 | 1.49 | undisturbed | | 1 | SG |
| -0.51 | 0.48 | 1.45 | undisturbed | | 1 | SG |
| -0.01 | 0.85 | 1.50 | undisturbed | | 1 | SG |
| -0.36 | 2.49 | 1.47 | undisturbed | | 1 | SG |
| -1.33 | 0.00 | 1.38 | 4 | | 2 | SG |
| -0.64 | 0.95 | 1.06 | 4 | | 2 | SG |
| -1.48 | 1.42 | 1.45 | 4 | | 2 | SG |
| 0.00 | 0.00 | 0.77 | 4 | | 2 | SG |
| -0.80 | 2.62 | 1.13 | 4 | | 2 | SG |
| -0.12 | 1.76 | 0.82 | 4 | | 2 | SG |
| -0.23 | 0.00 | 0.87 | 4 | | 2 | SG |
| -0.30 | 0.78 | 0.90 | 4 | | 2 | SG |
| -1.57 | 2.31 | 1.50 | 4 | | 2 | SG |
| -0.17 | 2.03 | 0.84 | 4 | | 2 | SG |
| -0.27 | 0.60 | 0.89 | 4 | | 2 | SG |
| -1.57 | 0.00 | 1.50 | 4 | | 2 | SG |
| -0.62 | 1.53 | 1.05 | 4 | | 2 | SG |
| -0.07 | 0.30 | 0.80 | 4 | | 2 | SG |
| -0.76 | 1.52 | 1.12 | 4 | | 2 | SG |
| -0.17 | 0.00 | 0.84 | 4 | | 2 | SG |
| -0.68 | 0.70 | 1.08 | 4 | | 2 | SG |
| -0.24 | 0.30 | 0.88 | 4 | | 2 | SG |
| -0.26 | 1.00 | 0.88 | 4 | | 2 | SG |
| -2.60 | 1.83 | 1.97 | 4 | | 2 | SG |
| -1.00 | 0.60 | 1.23 | 4 | | 2 | SG |
| -0.88 | 1.40 | 1.17 | 4 | | 2 | SG |
| -0.44 | 0.48 | 0.97 | 4 | | 2 | SG |
| -1.57 | 2.79 | 1.50 | 4 | | 2 | SG |
| -0.31 | 0.00 | 0.91 | 4 | | 2 | SG |
| -0.95 | 2.71 | 1.20 | 4 | | 2 | SG |
| -0.71 | 1.88 | 1.10 | 4 | | 2 | SG |
| -1.18 | 0.00 | 1.31 | 4 | | 2 | SG |
| -0.88 | 0.00 | 1.17 | 4 | | 2 | SG |
| -1.03 | 1.80 | 1.24 | 4 | | 2 | SG |
| -1.33 | 1.75 | 1.38 | 4 | | 2 | SG |
| -0.73 | 0.30 | 1.10 | 4 | | 2 | SG |
| -0.05 | 1.18 | 0.79 | 4 | | 2 | SG |
| -0.38 | 0.00 | 0.94 | 4 | | 2 | SG |
| -0.03 | 1.04 | 0.78 | 4 | | 2 | SG |
| -0.03 | 0.30 | 0.78 | 4 | | 2 | SG |
| -0.05 | 0.48 | 0.79 | 4 | | 2 | SG |
| -0.17 | 1.34 | 0.84 | 4 | | 2 | SG |
| -0.34 | 2.74 | 0.92 | 4 | | 2 | SG |
| -0.59 | 2.23 | 1.04 | 4 | | 2 | SG |
| -0.52 | 0.95 | 1.01 | 4 | | 2 | SG |
| -0.13 | 1.93 | 0.83 | 4 | | 2 | SG |
| -0.40 | 1.83 | 0.95 | 4 | | 2 | SG |
| -0.01 | 0.00 | 0.77 | 4 | | 2 | SG |
| -0.32 | 0.00 | 0.92 | 4 | | 2 | SG |
| -1.33 | 1.26 | 1.33 | 6 | | 2 | SG |
| -0.64 | 1.52 | 1.29 | 6 | | 2 | SG |
| -1.13 | 1.98 | 1.32 | 6 | | 2 | SG |
| -2.30 | 2.32 | 1.39 | 6 | | 2 | SG |
| -0.04 | 1.75 | 1.26 | 6 | | 2 | SG |
| -0.02 | 0.85 | 1.26 | 6 | | 2 | SG |
| -1.48 | 2.66 | 1.34 | 6 | | 2 | SG |
| 0.00 | 0.95 | 1.26 | 6 | | 2 | SG |
| -0.80 | 2.61 | 1.30 | 6 | | 2 | SG |
| -0.01 | 1.48 | 1.26 | 6 | | 2 | SG |
| -0.12 | 0.00 | 1.26 | 6 | | 2 | SG |
| -0.23 | 0.00 | 1.27 | 6 | | 2 | SG |
| -0.07 | 0.48 | 1.26 | 6 | | 2 | SG |
| -0.04 | 1.52 | 1.26 | 6 | | 2 | SG |
| -0.30 | 0.30 | 1.27 | 6 | | 2 | SG |
| -1.57 | 1.43 | 1.35 | 6 | | 2 | SG |
| -0.17 | 1.18 | 1.27 | 6 | | 2 | SG |
| -0.27 | 1.00 | 1.27 | 6 | | 2 | SG |
| -1.57 | 0.90 | 1.35 | 6 | | 2 | SG |
| -0.62 | 1.34 | 1.29 | 6 | | 2 | SG |
| -0.07 | 1.46 | 1.26 | 6 | | 2 | SG |
| -0.07 | 1.57 | 1.26 | 6 | | 2 | SG |
| -0.47 | 0.00 | 1.28 | 6 | | 2 | SG |
| -0.76 | 2.36 | 1.30 | 6 | | 2 | SG |
| -0.17 | 3.00 | 1.27 | 6 | | 2 | SG |
| -0.68 | 1.11 | 1.30 | 6 | | 2 | SG |
| -0.24 | 2.90 | 1.27 | 6 | | 2 | SG |
| -0.26 | 2.21 | 1.27 | 6 | | 2 | SG |
| -2.30 | 0.90 | 1.39 | 6 | | 2 | SG |
| -1.00 | 1.58 | 1.31 | 6 | | 2 | SG |
| -0.88 | 0.48 | 1.31 | 6 | | 2 | SG |
| -0.44 | 1.91 | 1.28 | 6 | | 2 | SG |
| -2.30 | 1.43 | 1.39 | 6 | | 2 | SG |
| -1.57 | 2.40 | 1.35 | 6 | | 2 | SG |
| -0.31 | 1.11 | 1.27 | 6 | | 2 | SG |
| -2.18 | 1.04 | 1.38 | 6 | | 2 | SG |
| -0.95 | 1.18 | 1.31 | 6 | | 2 | SG |
| -0.71 | 1.86 | 1.30 | 6 | | 2 | SG |
| -0.28 | 2.04 | 1.27 | 6 | | 2 | SG |
| -1.18 | 0.85 | 1.32 | 6 | | 2 | SG |
| -0.76 | 0.60 | 1.30 | 6 | | 2 | SG |
| -0.88 | 2.03 | 1.31 | 6 | | 2 | SG |
| -1.03 | 1.43 | 1.32 | 6 | | 2 | SG |
| -0.80 | 1.00 | 1.30 | 6 | | 2 | SG |
| -0.32 | 0.30 | 1.27 | 6 | | 2 | SG |
| -0.01 | 1.15 | 1.26 | 6 | | 2 | SG |
| -0.64 | 1.52 | 1.29 | 6 | | 2 | SG |
| -1.33 | 1.04 | 1.33 | 6 | | 2 | SG |
| -0.73 | 1.46 | 1.30 | 6 | | 2 | SG |
| -0.05 | 0.30 | 1.26 | 6 | | 2 | SG |
| -0.05 | 2.20 | 1.26 | 6 | | 2 | SG |
| -0.38 | 1.40 | 1.28 | 6 | | 2 | SG |
| -1.18 | 0.00 | 1.32 | 6 | | 2 | SG |
| -0.03 | 1.65 | 1.26 | 6 | | 2 | SG |
| -0.03 | 0.30 | 1.26 | 6 | | 2 | SG |
| -0.05 | 1.26 | 1.26 | 6 | | 2 | SG |
| -0.17 | 0.70 | 1.26 | 6 | | 2 | SG |
| -1.70 | 1.54 | 1.36 | 6 | | 2 | SG |
| -2.30 | 0.00 | 1.39 | 6 | | 2 | SG |
| -0.14 | 1.72 | 1.26 | 6 | | 2 | SG |
| -0.23 | 0.00 | 1.27 | 6 | | 2 | SG |
| -0.11 | 1.81 | 1.26 | 6 | | 2 | SG |
| -0.12 | 2.47 | 1.98 | 10 | | 2 | SG |
| -0.34 | 1.81 | 1.81 | 10 | | 2 | SG |
| -0.32 | 0.00 | 1.82 | 10 | | 2 | SG |
| -0.59 | 2.73 | 1.60 | 10 | | 2 | SG |
| -0.52 | 0.70 | 1.66 | 10 | | 2 | SG |
| -0.13 | 0.70 | 1.97 | 10 | | 2 | SG |
| -0.51 | 1.88 | 1.66 | 10 | | 2 | SG |
| -0.40 | 2.79 | 1.76 | 10 | | 2 | SG |
| -0.01 | 1.81 | 2.07 | 10 | | 2 | SG |
| -0.36 | 2.76 | 1.79 | 10 | | 2 | SG |
| -0.33 | 2.47 | 1.81 | 10 | | 2 | SG |
| -0.33 | 1.81 | 1.81 | 10 | | 2 | SG |
| -0.02 | 2.73 | 2.06 | 10 | | 2 | SG |
| -1.48 | 0.70 | 0.88 | 10 | | 2 | SG |
| -0.80 | 0.70 | 1.43 | 10 | | 2 | SG |
| -0.12 | 1.88 | 1.98 | 10 | | 2 | SG |
| -0.23 | 2.79 | 1.90 | 10 | | 2 | SG |
| -0.30 | 1.81 | 1.84 | 10 | | 2 | SG |
| -0.17 | 2.00 | 1.94 | 10 | | 2 | SG |
| -0.27 | 1.83 | 1.86 | 10 | | 2 | SG |
| -0.62 | 1.61 | 1.58 | 10 | | 2 | SG |
| -0.07 | 1.80 | 2.03 | 10 | | 2 | SG |
| -0.76 | 1.08 | 1.46 | 10 | | 2 | SG |
| -0.68 | 1.34 | 1.52 | 10 | | 2 | SG |
| -0.24 | 2.05 | 1.88 | 10 | | 2 | SG |
| -0.26 | 2.27 | 1.87 | 10 | | 2 | SG |
| -1.00 | 0.00 | 1.27 | 10 | | 2 | SG |
| -0.44 | 2.27 | 1.72 | 10 | | 2 | SG |
| -0.31 | 2.72 | 1.83 | 10 | | 2 | SG |
| -0.95 | 2.63 | 1.31 | 10 | | 2 | SG |
| -0.71 | 0.00 | 1.50 | 10 | | 2 | SG |
| -0.28 | 0.90 | 1.85 | 10 | | 2 | SG |
| -0.76 | 2.48 | 1.46 | 10 | | 2 | SG |
| -0.88 | 2.57 | 1.37 | 10 | | 2 | SG |
| -1.03 | 1.26 | 1.24 | 10 | | 2 | SG |
| -0.80 | 1.28 | 1.43 | 10 | | 2 | SG |
| -0.32 | 2.87 | 1.82 | 10 | | 2 | SG |
| -0.01 | 2.03 | 2.07 | 10 | | 2 | SG |
| -0.64 | 0.30 | 1.56 | 10 | | 2 | SG |
| -1.33 | 2.51 | 1.00 | 10 | | 2 | SG |
| -0.73 | 0.60 | 1.49 | 10 | | 2 | SG |
| -0.05 | 2.09 | 2.04 | 10 | | 2 | SG |
| -0.05 | 1.96 | 2.04 | 10 | | 2 | SG |
| -0.38 | 2.31 | 1.77 | 10 | | 2 | SG |
| -1.18 | 0.48 | 1.12 | 10 | | 2 | SG |
| -0.03 | 1.18 | 2.06 | 10 | | 2 | SG |
| -0.03 | 2.25 | 2.06 | 10 | | 2 | SG |
| -0.05 | 0.78 | 2.04 | 10 | | 2 | SG |
| -0.17 | 2.02 | 1.95 | 10 | | 2 | SG |
| -1.70 | 0.00 | 0.70 | 10 | | 2 | SG |
| -0.14 | 1.04 | 1.96 | 10 | | 2 | SG |
| -0.23 | 1.71 | 1.90 | 10 | | 2 | SG |
| -0.11 | 2.25 | 1.99 | 10 | | 2 | SG |
| -0.12 | 1.83 | 1.98 | 10 | | 2 | SG |
| -0.34 | 2.37 | 1.81 | 10 | | 2 | SG |
| -0.32 | 1.56 | 1.82 | 10 | | 2 | SG |
| -0.59 | 1.43 | 1.60 | 10 | | 2 | SG |
| -0.52 | 2.67 | 1.66 | 10 | | 2 | SG |
| -0.13 | 1.91 | 1.97 | 10 | | 2 | SG |
| -0.51 | 1.49 | 1.66 | 10 | | 2 | SG |
| -0.40 | 1.61 | 1.76 | 10 | | 2 | SG |
| -0.01 | 2.20 | 2.07 | 10 | | 2 | SG |
| -1.33 | 1.23 | 1.00 | 10 | | 2 | SG |
| -0.64 | 1.51 | 1.56 | 10 | | 2 | SG |
| -1.33 | 1.08 | 1.00 | 10 | | 2 | SG |
| -2.30 | 0.00 | 0.20 | 10 | | 2 | SG |
| -2.30 | 0.30 | 0.20 | 10 | | 2 | SG |
| -0.02 | 2.69 | 2.06 | 10 | | 2 | SG |
| -1.48 | 1.11 | 1.18 | 13 | | 2 | SG |
| -0.80 | 1.53 | 1.54 | 13 | | 2 | SG |
| -0.12 | 1.86 | 1.89 | 13 | | 2 | SG |
| -0.23 | 2.19 | 1.83 | 13 | | 2 | SG |
| -0.30 | 2.76 | 1.80 | 13 | | 2 | SG |
| -0.17 | 1.40 | 1.86 | 13 | | 2 | SG |
| -0.27 | 1.57 | 1.81 | 13 | | 2 | SG |
| -0.07 | 1.94 | 1.92 | 13 | | 2 | SG |
| -0.07 | 2.03 | 1.92 | 13 | | 2 | SG |
| -0.76 | 1.89 | 1.55 | 13 | | 2 | SG |
| -0.68 | 2.14 | 1.59 | 13 | | 2 | SG |
| -0.24 | 2.30 | 1.83 | 13 | | 2 | SG |
| -0.26 | 1.66 | 1.82 | 13 | | 2 | SG |
| -2.30 | 0.48 | 0.75 | 13 | | 2 | SG |
| -1.00 | 0.00 | 1.43 | 13 | | 2 | SG |
| -0.44 | 0.60 | 1.72 | 13 | | 2 | SG |
| -0.31 | 2.81 | 1.79 | 13 | | 2 | SG |
| -2.18 | 2.29 | 0.81 | 13 | | 2 | SG |
| -0.95 | 2.55 | 1.46 | 13 | | 2 | SG |
| -0.28 | 0.70 | 1.81 | 13 | | 2 | SG |
| -0.76 | 2.53 | 1.55 | 13 | | 2 | SG |
| -0.88 | 2.15 | 1.49 | 13 | | 2 | SG |
| -1.03 | 1.40 | 1.41 | 13 | | 2 | SG |
| -0.32 | 1.76 | 1.78 | 13 | | 2 | SG |
| -0.01 | 1.88 | 1.95 | 13 | | 2 | SG |
| -0.64 | 0.00 | 1.62 | 13 | | 2 | SG |
| -0.95 | 1.43 | 1.46 | 13 | | 2 | SG |
| -0.73 | 1.86 | 1.57 | 13 | | 2 | SG |
| -0.05 | 1.80 | 1.93 | 13 | | 2 | SG |
| -0.05 | 1.93 | 1.93 | 13 | | 2 | SG |
| -0.38 | 2.59 | 1.75 | 13 | | 2 | SG |
| -1.18 | 1.04 | 1.34 | 13 | | 2 | SG |
| -0.03 | 1.30 | 1.94 | 13 | | 2 | SG |
| -0.03 | 1.84 | 1.94 | 13 | | 2 | SG |
| -0.05 | 1.89 | 1.93 | 13 | | 2 | SG |
| -0.17 | 2.93 | 1.87 | 13 | | 2 | SG |
| -1.70 | 1.96 | 1.06 | 13 | | 2 | SG |
| -2.30 | 1.91 | 0.75 | 13 | | 2 | SG |
| -0.14 | 1.77 | 1.88 | 13 | | 2 | SG |
| -0.23 | 0.60 | 1.83 | 13 | | 2 | SG |
| -0.11 | 1.61 | 1.89 | 13 | | 2 | SG |
| -0.12 | 1.61 | 1.89 | 13 | | 2 | SG |
| -0.34 | 1.93 | 1.78 | 13 | | 2 | SG |
| -0.32 | 1.45 | 1.78 | 13 | | 2 | SG |
| -0.59 | 1.43 | 1.65 | 13 | | 2 | SG |
| -0.52 | 2.79 | 1.68 | 13 | | 2 | SG |
| -0.13 | 2.16 | 1.88 | 13 | | 2 | SG |
| -0.51 | 1.45 | 1.68 | 13 | | 2 | SG |
| -0.01 | 1.65 | 1.95 | 13 | | 2 | SG |
| -1.33 | 0.48 | 1.25 | 13 | | 2 | SG |
| -1.03 | 1.26 | 1.41 | 13 | | 2 | SG |
| -1.33 | 0.30 | 1.25 | 13 | | 2 | SG |
| -2.30 | 0.85 | 0.75 | 13 | | 2 | SG |
| -1.13 | 0.00 | 1.36 | 13 | | 2 | SG |
| -2.30 | 0.30 | 0.75 | 13 | | 2 | SG |
| -0.04 | 1.96 | 1.93 | 13 | | 2 | SG |
| -0.02 | 2.62 | 1.94 | 13 | | 2 | SG |
| -1.48 | 0.00 | 1.18 | 13 | | 2 | SG |
| 0.00 | 1.96 | 1.95 | 13 | | 2 | SG |
| -0.01 | 1.97 | 1.95 | 13 | | 2 | SG |
| -0.12 | 1.42 | 1.89 | 13 | | 2 | SG |
| -0.23 | 1.83 | 1.83 | 13 | | 2 | SG |
| -0.07 | 2.00 | 1.92 | 13 | | 2 | SG |
| -0.30 | 2.64 | 1.80 | 13 | | 2 | SG |
| -1.57 | 0.00 | 1.13 | 13 | | 2 | SG |
| -0.17 | 1.96 | 1.86 | 13 | | 2 | SG |
| -0.27 | 1.91 | 1.81 | 13 | | 2 | SG |
| -0.07 | 2.15 | 1.92 | 13 | | 2 | SG |
| -0.76 | 1.94 | 1.55 | 13 | | 2 | SG |
| -0.68 | 2.18 | 1.59 | 13 | | 2 | SG |
| -0.24 | 2.77 | 1.83 | 13 | | 2 | SG |
| -0.26 | 1.18 | 1.82 | 13 | | 2 | SG |
| -2.30 | 2.51 | 1.18 | undisturbed | | 2 | SG |
| -1.00 | 2.20 | 1.52 | undisturbed | | 2 | SG |
| -2.30 | 2.01 | 1.18 | undisturbed | | 2 | SG |
| -0.31 | 2.55 | 1.70 | undisturbed | | 2 | SG |
| -2.18 | 2.26 | 1.21 | undisturbed | | 2 | SG |
| -0.95 | 2.49 | 1.54 | undisturbed | | 2 | SG |
| -0.71 | 0.00 | 1.60 | undisturbed | | 2 | SG |
| -1.18 | 0.00 | 1.48 | undisturbed | | 2 | SG |
| -0.76 | 2.37 | 1.58 | undisturbed | | 2 | SG |
| -0.88 | 1.59 | 1.55 | undisturbed | | 2 | SG |
| -0.80 | 0.30 | 1.57 | undisturbed | | 2 | SG |
| -0.32 | 1.86 | 1.70 | undisturbed | | 2 | SG |
| -0.01 | 1.88 | 1.78 | undisturbed | | 2 | SG |
| -0.64 | 0.00 | 1.61 | undisturbed | | 2 | SG |
| -1.33 | 2.74 | 1.43 | undisturbed | | 2 | SG |
| -0.73 | 0.00 | 1.59 | undisturbed | | 2 | SG |
| -0.05 | 1.60 | 1.77 | undisturbed | | 2 | SG |
| -0.38 | 1.81 | 1.68 | undisturbed | | 2 | SG |
| -1.18 | 0.00 | 1.48 | undisturbed | | 2 | SG |
| -0.03 | 1.93 | 1.78 | undisturbed | | 2 | SG |
| -0.03 | 2.18 | 1.78 | undisturbed | | 2 | SG |
| -0.05 | 2.00 | 1.77 | undisturbed | | 2 | SG |
| -0.17 | 2.93 | 1.74 | undisturbed | | 2 | SG |
| -1.70 | 0.00 | 1.34 | undisturbed | | 2 | SG |
| -2.30 | 0.30 | 1.18 | undisturbed | | 2 | SG |
| -0.14 | 1.18 | 1.75 | undisturbed | | 2 | SG |
| -0.23 | 1.49 | 1.72 | undisturbed | | 2 | SG |
| -0.11 | 1.34 | 1.75 | undisturbed | | 2 | SG |
| -0.12 | 2.62 | 1.75 | undisturbed | | 2 | SG |
| -0.34 | 1.92 | 1.70 | undisturbed | | 2 | SG |
| -0.32 | 2.20 | 1.70 | undisturbed | | 2 | SG |
| -0.59 | 1.20 | 1.63 | undisturbed | | 2 | SG |
| -0.52 | 1.95 | 1.65 | undisturbed | | 2 | SG |
| -0.13 | 2.14 | 1.75 | undisturbed | | 2 | SG |
| -0.36 | 2.27 | 1.69 | undisturbed | | 2 | SG |
| 0.78 | 0.00 | 1.49 | 4 | | 1 | H |
| 1.20 | 2.84 | 1.58 | 4 | | 1 | H |
| 1.28 | 0.30 | 1.59 | 4 | | 1 | H |
| 1.05 | 1.69 | 1.55 | 4 | | 1 | H |
| 1.20 | 0.78 | 1.58 | 4 | | 1 | H |
| 1.50 | 0.30 | 1.64 | 4 | | 1 | H |
| 0.73 | 1.79 | 1.48 | 4 | | 1 | H |
| 1.32 | 1.79 | 1.60 | 4 | | 1 | H |
| 1.36 | 2.39 | 1.61 | 4 | | 1 | H |
| 1.33 | 3.10 | 1.61 | 4 | | 1 | H |
| 1.22 | 0.48 | 1.58 | 4 | | 1 | H |
| 1.59 | 1.28 | 1.66 | 4 | | 1 | H |
| 1.54 | 2.29 | 1.65 | 4 | | 1 | H |
| 0.40 | 1.96 | 1.42 | 4 | | 1 | H |
| 1.07 | 0.70 | 1.55 | 4 | | 1 | H |
| 1.26 | 1.26 | 1.59 | 4 | | 1 | H |
| 1.73 | 0.70 | 1.69 | 4 | | 1 | H |
| 1.42 | 2.89 | 1.62 | 4 | | 1 | H |
| 1.20 | 2.16 | 1.58 | 4 | | 1 | H |
| 1.33 | 1.18 | 1.61 | 4 | | 1 | H |
| 0.95 | 1.89 | 1.53 | 4 | | 1 | H |
| 1.64 | 2.56 | 1.67 | 4 | | 1 | H |
| 1.07 | 1.69 | 1.55 | 4 | | 1 | H |
| 1.23 | 1.99 | 1.58 | 4 | | 1 | H |
| 0.63 | 0.48 | 1.02 | 6 | | 1 | H |
| 1.41 | 1.88 | 1.65 | 6 | | 1 | H |
| 1.29 | 1.84 | 1.55 | 6 | | 1 | H |
| 1.75 | 0.70 | 1.93 | 6 | | 1 | H |
| 0.58 | 1.04 | 0.98 | 6 | | 1 | H |
| 0.88 | 1.40 | 1.22 | 6 | | 1 | H |
| 0.83 | 1.04 | 1.18 | 6 | | 1 | H |
| 1.00 | 0.00 | 1.32 | 6 | | 1 | H |
| 1.37 | 2.26 | 1.62 | 6 | | 1 | H |
| 1.60 | 3.00 | 1.80 | 6 | | 1 | H |
| 1.09 | 3.01 | 1.39 | 6 | | 1 | H |
| 1.77 | 1.99 | 1.94 | 6 | | 1 | H |
| 1.93 | 2.57 | 2.07 | 6 | | 1 | H |
| 0.93 | 1.08 | 1.26 | 6 | | 1 | H |
| 1.76 | 1.89 | 1.93 | 6 | | 1 | H |
| 1.59 | 1.20 | 1.80 | 6 | | 1 | H |
| 1.93 | 1.38 | 2.07 | 6 | | 1 | H |
| 1.27 | 1.53 | 1.54 | 6 | | 1 | H |
| 1.25 | 1.38 | 1.52 | 6 | | 1 | H |
| 1.19 | 1.59 | 1.47 | 6 | | 1 | H |
| 1.19 | 0.00 | 1.71 | 10 | | 1 | H |
| 1.35 | 1.43 | 1.74 | 10 | | 1 | H |
| 1.62 | 2.05 | 1.79 | 10 | | 1 | H |
| 1.46 | 1.20 | 1.76 | 10 | | 1 | H |
| 1.24 | 2.34 | 1.72 | 10 | | 1 | H |
| 1.12 | 1.81 | 1.70 | 10 | | 1 | H |
| 1.74 | 2.96 | 1.82 | 10 | | 1 | H |
| 0.94 | 2.47 | 1.67 | 10 | | 1 | H |
| 1.43 | 1.04 | 1.76 | 10 | | 1 | H |
| 0.95 | 1.98 | 1.67 | 10 | | 1 | H |
| 1.75 | 2.11 | 1.82 | 10 | | 1 | H |
| 1.75 | 2.11 | 1.82 | 10 | | 1 | H |
| 1.91 | 2.50 | 1.85 | 10 | | 1 | H |
| 1.20 | 1.32 | 1.72 | 10 | | 1 | H |
| 0.92 | 3.16 | 1.66 | 10 | | 1 | H |
| 1.02 | 1.26 | 1.68 | 10 | | 1 | H |
| 1.44 | 0.90 | 1.76 | 10 | | 1 | H |
| 1.56 | 2.22 | 1.78 | 10 | | 1 | H |
| 1.39 | 0.00 | 1.75 | 10 | | 1 | H |
| 1.54 | 2.52 | 1.78 | 10 | | 1 | H |
| 1.77 | 1.00 | 1.82 | 10 | | 1 | H |
| 1.74 | 2.89 | 1.82 | 10 | | 1 | H |
| 1.57 | 1.30 | 1.79 | 10 | | 1 | H |
| 1.98 | 1.87 | 1.86 | 10 | | 1 | H |
| 1.85 | 1.84 | 1.84 | 10 | | 1 | H |
| 1.36 | 1.60 | 1.75 | 10 | | 1 | H |
| 1.30 | 2.47 | 1.73 | 10 | | 1 | H |
| 1.73 | 1.04 | 1.82 | 10 | | 1 | H |
| 1.28 | 1.72 | 1.68 | 13 | | 1 | H |
| 1.11 | 2.81 | 1.79 | 13 | | 1 | H |
| 0.85 | 2.35 | 1.96 | 13 | | 1 | H |
| 1.07 | 0.95 | 1.82 | 13 | | 1 | H |
| 1.12 | 2.08 | 1.79 | 13 | | 1 | H |
| 1.51 | 0.60 | 1.53 | 13 | | 1 | H |
| 1.04 | 1.20 | 1.84 | 13 | | 1 | H |
| 0.60 | 2.42 | 2.13 | 13 | | 1 | H |
| 1.52 | 1.87 | 1.52 | 13 | | 1 | H |
| 0.70 | 2.11 | 2.06 | 13 | | 1 | H |
| 1.30 | 2.30 | 1.67 | 13 | | 1 | H |
| 1.26 | 1.74 | 1.69 | 13 | | 1 | H |
| 1.75 | 2.78 | 1.37 | 13 | | 1 | H |
| 1.56 | 2.06 | 1.50 | 13 | | 1 | H |
| 1.45 | 2.41 | 1.57 | 13 | | 1 | H |
| 1.08 | 2.38 | 1.81 | 13 | | 1 | H |
| 1.70 | 0.30 | 1.40 | 13 | | 1 | H |
| 1.75 | 2.65 | 1.37 | 13 | | 1 | H |
| 1.07 | 2.64 | 1.82 | 13 | | 1 | H |
| 0.78 | 1.67 | 2.01 | 13 | | 1 | H |
| 1.02 | 1.72 | 1.85 | 13 | | 1 | H |
| 1.54 | 0.78 | 1.51 | 13 | | 1 | H |
| 0.60 | 2.47 | 2.13 | 13 | | 1 | H |
| 1.05 | 2.09 | 1.83 | 13 | | 1 | H |
| 1.48 | 0.48 | 1.55 | 13 | | 1 | H |
| 1.33 | 2.55 | 1.65 | 13 | | 1 | H |
| 1.26 | 0.00 | 1.69 | 13 | | 1 | H |
| 1.80 | 1.00 | 1.34 | 13 | | 1 | H |
| 1.55 | 1.70 | 1.50 | 13 | | 1 | H |
| 1.53 | 2.95 | 1.52 | 13 | | 1 | H |
| 1.02 | 1.30 | 1.85 | 13 | | 1 | H |
| 1.49 | 1.74 | 1.54 | 13 | | 1 | H |
| 1.98 | 1.71 | 1.22 | 13 | | 1 | H |
| 1.78 | 0.60 | 1.35 | 13 | | 1 | H |
| 1.00 | 0.70 | 1.86 | 13 | | 1 | H |
| 1.59 | 1.91 | 1.48 | 13 | | 1 | H |
| 0.99 | 1.93 | 1.87 | 13 | | 1 | H |
| 1.76 | 1.30 | 1.36 | 13 | | 1 | H |
| 0.93 | 1.48 | 1.91 | 13 | | 1 | H |
| 0.90 | 2.20 | 1.93 | 13 | | 1 | H |
| 1.20 | 1.48 | 1.73 | 13 | | 1 | H |
| 1.57 | 0.60 | 1.49 | 13 | | 1 | H |
| 1.85 | 0.48 | 1.31 | 13 | | 1 | H |
| 1.65 | 1.04 | 1.44 | 13 | | 1 | H |
| 1.10 | 2.49 | 1.61 | Undisturbed | | 1 | H |
| 1.18 | 1.26 | 1.60 | Undisturbed | | 1 | H |
| 1.23 | 1.78 | 1.59 | Undisturbed | | 1 | H |
| 1.60 | 1.43 | 1.54 | Undisturbed | | 1 | H |
| 0.30 | 1.04 | 1.71 | Undisturbed | | 1 | H |
| 1.36 | 2.82 | 1.57 | Undisturbed | | 1 | H |
| 0.98 | 2.49 | 1.62 | Undisturbed | | 1 | H |
| 1.00 | 1.11 | 1.62 | Undisturbed | | 1 | H |
| 0.48 | 1.41 | 1.69 | Undisturbed | | 1 | H |
| 1.67 | 0.90 | 1.53 | Undisturbed | | 1 | H |
| 0.85 | 0.48 | 1.64 | Undisturbed | | 1 | H |
| 1.00 | 2.49 | 1.62 | Undisturbed | | 1 | H |
| 1.32 | 1.77 | 1.58 | Undisturbed | | 1 | H |
| 0.78 | 1.71 | 1.65 | Undisturbed | | 1 | H |
| 0.99 | 1.46 | 1.62 | Undisturbed | | 1 | H |
| 0.70 | 2.49 | 1.66 | Undisturbed | | 1 | H |
| 1.57 | 1.11 | 1.54 | Undisturbed | | 1 | H |
| 1.28 | 1.81 | 1.58 | Undisturbed | | 1 | H |
| 1.48 | 0.00 | 1.56 | Undisturbed | | 1 | H |
| 1.15 | 2.59 | 1.60 | Undisturbed | | 1 | H |
| 1.40 | 2.49 | 1.57 | Undisturbed | | 1 | H |
| 1.82 | 2.42 | 1.51 | Undisturbed | | 1 | H |
| 1.41 | 2.21 | 1.56 | Undisturbed | | 1 | H |
| 0.91 | 2.32 | 1.63 | Undisturbed | | 1 | H |
| 0.30 | 1.93 | 1.71 | Undisturbed | | 1 | H |
| 1.26 | 0.00 | 1.58 | Undisturbed | | 1 | H |
| 0.30 | 2.30 | 1.71 | Undisturbed | | 1 | H |
| 1.55 | 1.61 | 1.55 | Undisturbed | | 1 | H |
| 1.78 | 2.47 | 1.52 | Undisturbed | | 1 | H |
| 1.00 | 1.00 | 1.62 | Undisturbed | | 1 | H |
| 1.00 | 2.17 | 1.62 | Undisturbed | | 1 | H |
| 0.97 | 1.59 | 1.62 | Undisturbed | | 1 | H |
| 0.93 | 1.95 | 1.63 | Undisturbed | | 1 | H |
| 1.81 | 0.48 | 1.51 | Undisturbed | | 1 | H |
| 1.33 | 2.70 | 1.58 | Undisturbed | | 1 | H |
| 1.02 | 2.28 | 1.62 | Undisturbed | | 1 | H |
| 0.87 | 1.88 | 1.64 | Undisturbed | | 1 | H |
| 0.48 | 1.18 | 1.69 | Undisturbed | | 1 | H |
| 1.22 | 0.78 | 1.59 | Undisturbed | | 1 | H |
| 1.45 | 1.79 | 1.56 | Undisturbed | | 1 | H |
| 1.50 | 1.04 | 1.55 | Undisturbed | | 1 | H |
| 1.52 | 1.04 | 1.55 | Undisturbed | | 1 | H |
| 1.00 | 0.30 | 1.62 | Undisturbed | | 1 | H |
| 1.64 | 0.30 | 1.53 | Undisturbed | | 1 | H |
| 1.32 | 1.74 | 1.58 | Undisturbed | | 1 | H |
| 1.05 | 2.16 | 1.61 | Undisturbed | | 1 | H |
| 1.51 | 3.00 | 1.55 | Undisturbed | | 1 | H |
| 1.08 | 1.75 | 1.61 | Undisturbed | | 1 | H |
| 1.09 | 1.41 | 1.61 | Undisturbed | | 1 | H |
| 1.91 | 2.05 | 1.50 | Undisturbed | | 1 | H |
| 1.20 | 1.30 | 1.59 | Undisturbed | | 1 | H |
| 1.71 | 2.19 | 1.52 | Undisturbed | | 1 | H |
| 0.48 | 1.41 | 1.69 | Undisturbed | | 1 | H |
| 1.10 | 0.30 | 1.61 | Undisturbed | | 1 | H |
| 1.72 | 1.54 | 1.52 | Undisturbed | | 1 | H |
| 0.92 | 0.00 | 1.63 | Undisturbed | | 1 | H |
| 1.08 | 2.36 | 1.61 | Undisturbed | | 1 | H |
| 1.54 | 1.49 | 1.55 | Undisturbed | | 1 | H |
| 1.82 | 0.30 | 1.51 | Undisturbed | | 1 | H |
| 1.65 | 0.85 | 1.53 | Undisturbed | | 1 | H |
| 1.22 | 2.49 | 1.59 | Undisturbed | | 1 | H |
| 1.21 | 3.02 | 1.56 | 4 | | 2 | H |
| 1.28 | 0.00 | 1.56 | 4 | | 2 | H |
| 1.05 | 1.53 | 1.55 | 4 | | 2 | H |
| 1.20 | 1.52 | 1.56 | 4 | | 2 | H |
| 1.50 | 0.00 | 1.57 | 4 | | 2 | H |
| 0.73 | 1.00 | 1.53 | 4 | | 2 | H |
| 1.32 | 1.40 | 1.56 | 4 | | 2 | H |
| 1.14 | 2.79 | 1.55 | 4 | | 2 | H |
| 1.06 | 3.05 | 1.55 | 4 | | 2 | H |
| 1.22 | 0.00 | 1.56 | 4 | | 2 | H |
| 1.59 | 1.80 | 1.58 | 4 | | 2 | H |
| 1.54 | 1.99 | 1.57 | 4 | | 2 | H |
| 0.40 | 1.75 | 1.52 | 4 | | 2 | H |
| 1.07 | 0.30 | 1.55 | 4 | | 2 | H |
| 1.26 | 1.18 | 1.56 | 4 | | 2 | H |
| 1.42 | 2.74 | 1.57 | 4 | | 2 | H |
| 1.17 | 0.60 | 1.56 | 4 | | 2 | H |
| 1.20 | 2.23 | 1.56 | 4 | | 2 | H |
| 1.33 | 0.95 | 1.56 | 4 | | 2 | H |
| 1.37 | 1.93 | 1.57 | 4 | | 2 | H |
| 1.64 | 2.11 | 1.58 | 4 | | 2 | H |
| 1.07 | 1.83 | 1.55 | 4 | | 2 | H |
| 1.23 | 2.10 | 1.56 | 4 | | 2 | H |
| 1.41 | 1.52 | 1.74 | 6 | | 2 | H |
| 1.29 | 1.43 | 1.66 | 6 | | 2 | H |
| 1.75 | 1.18 | 1.97 | 6 | | 2 | H |
| 0.58 | 0.90 | 1.19 | 6 | | 2 | H |
| 0.88 | 1.34 | 1.39 | 6 | | 2 | H |
| 0.83 | 1.57 | 1.36 | 6 | | 2 | H |
| 1.37 | 2.36 | 1.71 | 6 | | 2 | H |
| 1.60 | 3.00 | 1.87 | 6 | | 2 | H |
| 1.09 | 2.90 | 1.53 | 6 | | 2 | H |
| 1.77 | 1.43 | 1.98 | 6 | | 2 | H |
| 1.93 | 2.40 | 2.09 | 6 | | 2 | H |
| 0.93 | 0.60 | 1.42 | 6 | | 2 | H |
| 1.76 | 2.03 | 1.97 | 6 | | 2 | H |
| 1.59 | 1.43 | 1.86 | 6 | | 2 | H |
| 1.27 | 1.26 | 1.65 | 6 | | 2 | H |
| 1.25 | 1.54 | 1.63 | 6 | | 2 | H |
| 1.19 | 1.72 | 1.59 | 6 | | 2 | H |
| 1.28 | 2.19 | 1.90 | 10 | | 2 | H |
| 1.11 | 2.76 | 2.00 | 10 | | 2 | H |
| 0.85 | 2.56 | 2.15 | 10 | | 2 | H |
| 1.18 | 2.11 | 1.96 | 10 | | 2 | H |
| 1.07 | 0.85 | 2.02 | 10 | | 2 | H |
| 1.12 | 2.03 | 1.99 | 10 | | 2 | H |
| 1.51 | 0.90 | 1.77 | 10 | | 2 | H |
| 1.04 | 1.89 | 2.04 | 10 | | 2 | H |
| 0.60 | 2.30 | 2.29 | 10 | | 2 | H |
| 1.52 | 1.66 | 1.76 | 10 | | 2 | H |
| 0.70 | 2.58 | 2.23 | 10 | | 2 | H |
| 1.30 | 1.90 | 1.89 | 10 | | 2 | H |
| 1.02 | 0.60 | 2.05 | 10 | | 2 | H |
| 1.75 | 2.81 | 1.63 | 10 | | 2 | H |
| 1.56 | 2.29 | 1.74 | 10 | | 2 | H |
| 1.45 | 2.55 | 1.80 | 10 | | 2 | H |
| 1.08 | 2.53 | 2.01 | 10 | | 2 | H |
| 1.70 | 1.40 | 1.66 | 10 | | 2 | H |
| 1.75 | 2.35 | 1.63 | 10 | | 2 | H |
| 1.07 | 2.68 | 2.02 | 10 | | 2 | H |
| 0.78 | 1.76 | 2.19 | 10 | | 2 | H |
| 1.02 | 1.88 | 2.05 | 10 | | 2 | H |
| 1.54 | 0.00 | 1.75 | 10 | | 2 | H |
| 0.60 | 2.77 | 2.29 | 10 | | 2 | H |
| 1.05 | 2.27 | 2.03 | 10 | | 2 | H |
| 1.36 | 1.43 | 1.85 | 10 | | 2 | H |
| 1.48 | 1.86 | 1.78 | 10 | | 2 | H |
| 1.44 | 1.80 | 1.81 | 10 | | 2 | H |
| 1.33 | 2.59 | 1.87 | 10 | | 2 | H |
| 1.66 | 1.11 | 1.68 | 10 | | 2 | H |
| 1.26 | 1.04 | 1.91 | 10 | | 2 | H |
| 1.80 | 1.30 | 1.60 | 10 | | 2 | H |
| 1.55 | 1.84 | 1.74 | 10 | | 2 | H |
| 1.53 | 2.93 | 1.76 | 10 | | 2 | H |
| 1.02 | 1.96 | 2.05 | 10 | | 2 | H |
| 1.49 | 1.91 | 1.78 | 10 | | 2 | H |
| 1.78 | 1.77 | 1.61 | 10 | | 2 | H |
| 1.00 | 1.61 | 2.06 | 10 | | 2 | H |
| 1.59 | 1.93 | 1.72 | 10 | | 2 | H |
| 1.76 | 1.45 | 1.62 | 10 | | 2 | H |
| 0.93 | 1.43 | 2.10 | 10 | | 2 | H |
| 0.90 | 2.79 | 2.12 | 10 | | 2 | H |
| 1.20 | 2.16 | 1.94 | 10 | | 2 | H |
| 1.57 | 1.45 | 1.73 | 10 | | 2 | H |
| 1.85 | 1.18 | 1.57 | 10 | | 2 | H |
| 1.65 | 1.65 | 1.69 | 10 | | 2 | H |
| 1.35 | 0.48 | 1.96 | 13 | | 2 | H |
| 1.62 | 1.97 | 1.89 | 13 | | 2 | H |
| 1.24 | 2.27 | 1.99 | 13 | | 2 | H |
| 1.12 | 2.27 | 2.02 | 13 | | 2 | H |
| 1.74 | 2.47 | 1.86 | 13 | | 2 | H |
| 0.94 | 2.72 | 2.07 | 13 | | 2 | H |
| 0.95 | 2.63 | 2.07 | 13 | | 2 | H |
| 1.75 | 2.48 | 1.86 | 13 | | 2 | H |
| 1.75 | 2.57 | 1.86 | 13 | | 2 | H |
| 1.91 | 1.26 | 1.82 | 13 | | 2 | H |
| 1.20 | 2.23 | 2.00 | 13 | | 2 | H |
| 0.92 | 1.83 | 2.08 | 13 | | 2 | H |
| 1.02 | 2.51 | 2.05 | 13 | | 2 | H |
| 1.44 | 2.00 | 1.94 | 13 | | 2 | H |
| 1.56 | 0.60 | 1.91 | 13 | | 2 | H |
| 1.54 | 2.09 | 1.91 | 13 | | 2 | H |
| 1.77 | 2.31 | 1.86 | 13 | | 2 | H |
| 1.74 | 1.11 | 1.86 | 13 | | 2 | H |
| 1.57 | 2.21 | 1.91 | 13 | | 2 | H |
| 1.85 | 2.38 | 1.83 | 13 | | 2 | H |
| 1.36 | 1.04 | 1.96 | 13 | | 2 | H |
| 1.30 | 0.85 | 1.98 | 13 | | 2 | H |
| 1.70 | 2.67 | 1.87 | 13 | | 2 | H |
| 1.73 | 1.49 | 1.87 | 13 | | 2 | H |
| 1.10 | 0.30 | 1.57 | Undisturbed | | 2 | H |
| 1.18 | 1.96 | 1.55 | Undisturbed | | 2 | H |
| 1.23 | 2.62 | 1.53 | Undisturbed | | 2 | H |
| 1.60 | 0.00 | 1.42 | Undisturbed | | 2 | H |
| 1.22 | 0.48 | 1.53 | Undisturbed | | 2 | H |
| 0.30 | 1.41 | 1.81 | Undisturbed | | 2 | H |
| 1.36 | 1.83 | 1.49 | Undisturbed | | 2 | H |
| 0.98 | 1.04 | 1.61 | Undisturbed | | 2 | H |
| 1.00 | 2.64 | 1.60 | Undisturbed | | 2 | H |
| 0.48 | 2.63 | 1.76 | Undisturbed | | 2 | H |
| 1.67 | 1.46 | 1.40 | Undisturbed | | 2 | H |
| 0.85 | 0.30 | 1.64 | Undisturbed | | 2 | H |
| 1.00 | 0.30 | 1.60 | Undisturbed | | 2 | H |
| 1.32 | 0.30 | 1.50 | Undisturbed | | 2 | H |
| 0.78 | 2.19 | 1.67 | Undisturbed | | 2 | H |
| 0.99 | 2.15 | 1.60 | Undisturbed | | 2 | H |
| 0.70 | 1.94 | 1.69 | Undisturbed | | 2 | H |
| 1.57 | 2.18 | 1.43 | Undisturbed | | 2 | H |
| 1.28 | 2.77 | 1.52 | Undisturbed | | 2 | H |
| 1.15 | 1.18 | 1.55 | Undisturbed | | 2 | H |
| 1.40 | 2.20 | 1.48 | Undisturbed | | 2 | H |
| 1.82 | 2.54 | 1.35 | Undisturbed | | 2 | H |
| 1.41 | 2.01 | 1.48 | Undisturbed | | 2 | H |
| 0.91 | 2.55 | 1.63 | Undisturbed | | 2 | H |
| 1.26 | 2.26 | 1.52 | Undisturbed | | 2 | H |
| 0.30 | 2.49 | 1.81 | Undisturbed | | 2 | H |
| 1.55 | 0.00 | 1.43 | Undisturbed | | 2 | H |
| 1.78 | 2.37 | 1.36 | Undisturbed | | 2 | H |
| 1.00 | 1.59 | 1.60 | Undisturbed | | 2 | H |
| 1.00 | 2.12 | 1.60 | Undisturbed | | 2 | H |
| 0.97 | 0.30 | 1.61 | Undisturbed | | 2 | H |
| 0.93 | 1.99 | 1.62 | Undisturbed | | 2 | H |
| 1.81 | 1.86 | 1.36 | Undisturbed | | 2 | H |
| 1.33 | 1.88 | 1.50 | Undisturbed | | 2 | H |
| 1.02 | 0.00 | 1.59 | Undisturbed | | 2 | H |
| 0.87 | 2.74 | 1.64 | Undisturbed | | 2 | H |
| 0.48 | 2.60 | 1.76 | Undisturbed | | 2 | H |
| 1.45 | 0.00 | 1.46 | Undisturbed | | 2 | H |
| 1.50 | 1.60 | 1.45 | Undisturbed | | 2 | H |
| 1.52 | 1.81 | 1.44 | Undisturbed | | 2 | H |
| 1.00 | 0.30 | 1.60 | Undisturbed | | 2 | H |
| 1.64 | 0.48 | 1.41 | Undisturbed | | 2 | H |
| 1.32 | 0.00 | 1.50 | Undisturbed | | 2 | H |
| 1.05 | 0.78 | 1.58 | Undisturbed | | 2 | H |
| 1.51 | 2.18 | 1.45 | Undisturbed | | 2 | H |
| 1.08 | 2.00 | 1.58 | Undisturbed | | 2 | H |
| 1.09 | 2.93 | 1.57 | Undisturbed | | 2 | H |
| 1.20 | 2.10 | 1.54 | Undisturbed | | 2 | H |
| 1.71 | 1.00 | 1.39 | Undisturbed | | 2 | H |
| 0.48 | 1.34 | 1.76 | Undisturbed | | 2 | H |
| 1.10 | 1.92 | 1.57 | Undisturbed | | 2 | H |
| 1.72 | 2.00 | 1.38 | Undisturbed | | 2 | H |
| 0.92 | 0.00 | 1.62 | Undisturbed | | 2 | H |
| 1.08 | 2.20 | 1.58 | Undisturbed | | 2 | H |
| 1.59 | 1.95 | 1.42 | Undisturbed | | 2 | H |
| 1.54 | 2.14 | 1.44 | Undisturbed | | 2 | H |
| 1.82 | 0.30 | 1.35 | Undisturbed | | 2 | H |
| 1.22 | 1.20 | 1.53 | Undisturbed | | 2 | H |
| 1.16 | 1.92 | 1.54 | 4 | | 1 | A |
| 1.30 | 1.30 | 1.60 | 4 | | 1 | A |
| 0.78 | 1.92 | 1.37 | 4 | | 1 | A |
| 1.17 | 1.52 | 1.54 | 4 | | 1 | A |
| 1.31 | 2.40 | 1.60 | 4 | | 1 | A |
| 1.33 | 0.85 | 1.61 | 4 | | 1 | A |
| 0.77 | 2.29 | 1.37 | 4 | | 1 | A |
| 0.87 | 1.65 | 1.41 | 4 | | 1 | A |
| 0.79 | 0.00 | 1.38 | 4 | | 1 | A |
| 1.13 | 2.84 | 1.52 | 4 | | 1 | A |
| 1.22 | 0.30 | 1.57 | 4 | | 1 | A |
| 1.17 | 1.69 | 1.54 | 4 | | 1 | A |
| 1.15 | 0.78 | 1.53 | 4 | | 1 | A |
| 1.13 | 0.30 | 1.52 | 4 | | 1 | A |
| 1.25 | 1.79 | 1.58 | 4 | | 1 | A |
| 0.90 | 2.57 | 1.43 | 4 | | 1 | A |
| 1.07 | 1.79 | 1.50 | 4 | | 1 | A |
| 1.41 | 0.00 | 1.64 | 4 | | 1 | A |
| 1.28 | 2.39 | 1.59 | 4 | | 1 | A |
| 1.21 | 3.10 | 1.56 | 4 | | 1 | A |
| 0.84 | 1.90 | 1.40 | 4 | | 1 | A |
| 1.03 | 0.48 | 1.48 | 4 | | 1 | A |
| 1.06 | 0.00 | 1.50 | 4 | | 1 | A |
| 1.19 | 1.95 | 1.55 | 4 | | 1 | A |
| 1.28 | 2.24 | 1.59 | 4 | | 1 | A |
| 0.76 | 0.48 | 1.36 | 4 | | 1 | A |
| 1.41 | 2.29 | 1.65 | 4 | | 1 | A |
| 1.06 | 1.96 | 1.49 | 4 | | 1 | A |
| 1.09 | 0.70 | 1.51 | 4 | | 1 | A |
| 1.07 | 1.26 | 1.50 | 4 | | 1 | A |
| 1.27 | 1.08 | 1.59 | 4 | | 1 | A |
| 1.13 | 0.70 | 1.52 | 4 | | 1 | A |
| 1.24 | 2.89 | 1.57 | 4 | | 1 | A |
| 1.27 | 2.16 | 1.59 | 4 | | 1 | A |
| 0.98 | 1.18 | 1.46 | 4 | | 1 | A |
| 1.22 | 1.89 | 1.56 | 4 | | 1 | A |
| 1.43 | 2.56 | 1.66 | 4 | | 1 | A |
| 0.89 | 1.69 | 1.42 | 4 | | 1 | A |
| 1.31 | 1.99 | 1.60 | 4 | | 1 | A |
| 1.46 | 0.30 | 1.67 | 4 | | 1 | A |
| 1.08 | 1.84 | 1.57 | 6 | | 1 | A |
| 1.15 | 0.70 | 1.60 | 6 | | 1 | A |
| 1.20 | 1.68 | 1.62 | 6 | | 1 | A |
| 0.87 | 1.04 | 1.48 | 6 | | 1 | A |
| 1.13 | 1.40 | 1.59 | 6 | | 1 | A |
| 1.01 | 1.04 | 1.54 | 6 | | 1 | A |
| 1.04 | 0.00 | 1.55 | 6 | | 1 | A |
| 1.03 | 2.26 | 1.55 | 6 | | 1 | A |
| 1.25 | 2.26 | 1.64 | 6 | | 1 | A |
| 1.29 | 3.01 | 1.65 | 6 | | 1 | A |
| 1.18 | 1.95 | 1.61 | 6 | | 1 | A |
| 1.33 | 1.81 | 1.67 | 6 | | 1 | A |
| 1.06 | 1.99 | 1.56 | 6 | | 1 | A |
| 1.28 | 2.57 | 1.65 | 6 | | 1 | A |
| 1.17 | 1.60 | 1.61 | 6 | | 1 | A |
| 1.04 | 2.02 | 1.55 | 6 | | 1 | A |
| 1.23 | 1.08 | 1.63 | 6 | | 1 | A |
| 1.10 | 1.89 | 1.58 | 6 | | 1 | A |
| 1.35 | 1.20 | 1.68 | 6 | | 1 | A |
| 1.40 | 1.18 | 1.70 | 6 | | 1 | A |
| 1.08 | 1.38 | 1.57 | 6 | | 1 | A |
| 1.00 | 1.71 | 1.54 | 6 | | 1 | A |
| 1.20 | 1.38 | 1.62 | 6 | | 1 | A |
| 1.46 | 1.00 | 1.73 | 6 | | 1 | A |
| 1.07 | 2.32 | 1.56 | 6 | | 1 | A |
| 1.20 | 1.53 | 1.62 | 6 | | 1 | A |
| 1.05 | 1.38 | 1.56 | 6 | | 1 | A |
| 1.13 | 1.59 | 1.59 | 6 | | 1 | A |
| 1.29 | 1.20 | 1.49 | 10 | | 1 | A |
| 1.03 | 0.90 | 1.84 | 10 | | 1 | A |
| 1.24 | 1.66 | 1.56 | 10 | | 1 | A |
| 1.34 | 1.48 | 1.41 | 10 | | 1 | A |
| 0.70 | 1.52 | 2.28 | 10 | | 1 | A |
| 1.31 | 1.20 | 1.45 | 10 | | 1 | A |
| 1.37 | 1.80 | 1.37 | 10 | | 1 | A |
| 1.32 | 2.16 | 1.44 | 10 | | 1 | A |
| 1.19 | 2.07 | 1.62 | 10 | | 1 | A |
| 1.44 | 0.30 | 1.28 | 10 | | 1 | A |
| 1.06 | 1.68 | 1.80 | 10 | | 1 | A |
| 1.09 | 0.60 | 1.76 | 10 | | 1 | A |
| 1.43 | 0.00 | 1.29 | 10 | | 1 | A |
| 1.28 | 1.43 | 1.50 | 10 | | 1 | A |
| 1.43 | 2.05 | 1.29 | 10 | | 1 | A |
| 1.12 | 1.94 | 1.72 | 10 | | 1 | A |
| 1.13 | 1.58 | 1.70 | 10 | | 1 | A |
| 1.13 | 1.20 | 1.71 | 10 | | 1 | A |
| 1.24 | 2.34 | 1.55 | 10 | | 1 | A |
| 1.10 | 1.81 | 1.74 | 10 | | 1 | A |
| 1.04 | 2.00 | 1.82 | 10 | | 1 | A |
| 0.98 | 2.96 | 1.91 | 10 | | 1 | A |
| 1.09 | 1.92 | 1.75 | 10 | | 1 | A |
| 1.00 | 2.47 | 1.88 | 10 | | 1 | A |
| 1.17 | 1.04 | 1.65 | 10 | | 1 | A |
| 1.17 | 1.98 | 1.65 | 10 | | 1 | A |
| 1.16 | 2.11 | 1.67 | 10 | | 1 | A |
| 1.19 | 2.11 | 1.62 | 10 | | 1 | A |
| 1.17 | 2.50 | 1.65 | 10 | | 1 | A |
| 1.22 | 1.32 | 1.58 | 10 | | 1 | A |
| 1.17 | 1.36 | 1.65 | 10 | | 1 | A |
| 1.06 | 1.87 | 1.79 | 10 | | 1 | A |
| 0.88 | 3.16 | 2.04 | 10 | | 1 | A |
| 1.09 | 1.26 | 1.75 | 10 | | 1 | A |
| 1.33 | 0.90 | 1.43 | 10 | | 1 | A |
| 1.13 | 2.22 | 1.71 | 10 | | 1 | A |
| 1.37 | 0.00 | 1.38 | 10 | | 1 | A |
| 1.25 | 2.52 | 1.53 | 10 | | 1 | A |
| 1.17 | 1.00 | 1.64 | 10 | | 1 | A |
| 1.59 | 1.08 | 1.08 | 10 | | 1 | A |
| 1.28 | 1.42 | 1.50 | 10 | | 1 | A |
| 1.22 | 2.89 | 1.58 | 10 | | 1 | A |
| 0.94 | 1.30 | 1.97 | 10 | | 1 | A |
| 1.14 | 1.87 | 1.69 | 10 | | 1 | A |
| 1.64 | 1.84 | 1.01 | 10 | | 1 | A |
| 1.10 | 1.30 | 1.75 | 10 | | 1 | A |
| 1.15 | 1.60 | 1.68 | 10 | | 1 | A |
| 1.19 | 2.47 | 1.62 | 10 | | 1 | A |
| 1.20 | 0.30 | 1.61 | 10 | | 1 | A |
| 1.39 | 1.04 | 1.35 | 10 | | 1 | A |
| 0.89 | 2.56 | 2.09 | 13 | | 1 | A |
| 1.05 | 1.62 | 1.86 | 13 | | 1 | A |
| 1.22 | 1.08 | 1.62 | 13 | | 1 | A |
| 0.91 | 1.96 | 2.07 | 13 | | 1 | A |
| 1.34 | 0.00 | 1.44 | 13 | | 1 | A |
| 1.59 | 1.94 | 1.09 | 13 | | 1 | A |
| 1.19 | 1.72 | 1.66 | 13 | | 1 | A |
| 1.04 | 2.81 | 1.88 | 13 | | 1 | A |
| 1.12 | 2.35 | 1.76 | 13 | | 1 | A |
| 1.35 | 0.95 | 1.44 | 13 | | 1 | A |
| 1.10 | 2.08 | 1.80 | 13 | | 1 | A |
| 1.12 | 0.60 | 1.77 | 13 | | 1 | A |
| 1.32 | 1.20 | 1.48 | 13 | | 1 | A |
| 0.89 | 2.42 | 2.09 | 13 | | 1 | A |
| 1.14 | 1.87 | 1.73 | 13 | | 1 | A |
| 0.98 | 2.30 | 1.97 | 13 | | 1 | A |
| 1.28 | 1.74 | 1.54 | 13 | | 1 | A |
| 1.24 | 2.78 | 1.59 | 13 | | 1 | A |
| 1.14 | 2.06 | 1.73 | 13 | | 1 | A |
| 1.25 | 2.41 | 1.57 | 13 | | 1 | A |
| 1.28 | 2.38 | 1.54 | 13 | | 1 | A |
| 1.16 | 1.98 | 1.70 | 13 | | 1 | A |
| 1.10 | 0.30 | 1.79 | 13 | | 1 | A |
| 1.47 | 2.65 | 1.26 | 13 | | 1 | A |
| 1.10 | 2.64 | 1.79 | 13 | | 1 | A |
| 1.22 | 1.67 | 1.62 | 13 | | 1 | A |
| 1.24 | 1.72 | 1.59 | 13 | | 1 | A |
| 1.52 | 0.78 | 1.19 | 13 | | 1 | A |
| 1.03 | 2.47 | 1.90 | 13 | | 1 | A |
| 1.21 | 2.09 | 1.63 | 13 | | 1 | A |
| 1.37 | 0.48 | 1.41 | 13 | | 1 | A |
| 1.16 | 2.55 | 1.70 | 13 | | 1 | A |
| 1.02 | 0.00 | 1.91 | 13 | | 1 | A |
| 1.61 | 1.00 | 1.06 | 13 | | 1 | A |
| 1.23 | 1.70 | 1.60 | 13 | | 1 | A |
| 1.19 | 2.95 | 1.66 | 13 | | 1 | A |
| 1.21 | 1.30 | 1.63 | 13 | | 1 | A |
| 0.98 | 1.74 | 1.97 | 13 | | 1 | A |
| 1.06 | 1.71 | 1.85 | 13 | | 1 | A |
| 1.40 | 0.60 | 1.36 | 13 | | 1 | A |
| 1.00 | 0.70 | 1.94 | 13 | | 1 | A |
| 1.25 | 1.91 | 1.57 | 13 | | 1 | A |
| 1.05 | 1.93 | 1.87 | 13 | | 1 | A |
| 1.31 | 1.30 | 1.49 | 13 | | 1 | A |
| 1.08 | 1.48 | 1.82 | 13 | | 1 | A |
| 1.10 | 2.20 | 1.80 | 13 | | 1 | A |
| 1.05 | 1.48 | 1.87 | 13 | | 1 | A |
| 1.28 | 0.60 | 1.54 | 13 | | 1 | A |
| 1.65 | 0.48 | 1.00 | 13 | | 1 | A |
| 1.59 | 1.04 | 1.10 | 13 | | 1 | A |
| 1.30 | 0.00 | 1.36 | Undisturbed | | 1 | A |
| 1.13 | 1.78 | 1.55 | Undisturbed | | 1 | A |
| 1.41 | 1.43 | 1.23 | Undisturbed | | 1 | A |
| 1.17 | 1.04 | 1.50 | Undisturbed | | 1 | A |
| 0.95 | 2.82 | 1.76 | Undisturbed | | 1 | A |
| 0.99 | 2.49 | 1.71 | Undisturbed | | 1 | A |
| 0.98 | 1.11 | 1.72 | Undisturbed | | 1 | A |
| 0.98 | 1.42 | 1.72 | Undisturbed | | 1 | A |
| 1.70 | 0.90 | 0.91 | Undisturbed | | 1 | A |
| 1.24 | 0.48 | 1.43 | Undisturbed | | 1 | A |
| 1.24 | 2.49 | 1.43 | Undisturbed | | 1 | A |
| 1.10 | 1.77 | 1.59 | Undisturbed | | 1 | A |
| 1.23 | 1.71 | 1.43 | Undisturbed | | 1 | A |
| 1.18 | 1.46 | 1.49 | Undisturbed | | 1 | A |
| 0.56 | 2.49 | 2.21 | Undisturbed | | 1 | A |
| 1.11 | 1.11 | 1.58 | Undisturbed | | 1 | A |
| 1.20 | 1.81 | 1.47 | Undisturbed | | 1 | A |
| 1.19 | 0.00 | 1.48 | Undisturbed | | 1 | A |
| 0.78 | 2.59 | 1.95 | Undisturbed | | 1 | A |
| 1.18 | 2.49 | 1.49 | Undisturbed | | 1 | A |
| 1.24 | 2.42 | 1.42 | Undisturbed | | 1 | A |
| 1.29 | 2.21 | 1.38 | Undisturbed | | 1 | A |
| 1.35 | 2.32 | 1.30 | Undisturbed | | 1 | A |
| 1.03 | 0.30 | 1.67 | Undisturbed | | 1 | A |
| 0.59 | 1.93 | 2.17 | Undisturbed | | 1 | A |
| 1.12 | 0.00 | 1.56 | Undisturbed | | 1 | A |
| 0.88 | 2.30 | 1.84 | Undisturbed | | 1 | A |
| 1.13 | 1.61 | 1.55 | Undisturbed | | 1 | A |
| 1.26 | 2.47 | 1.41 | Undisturbed | | 1 | A |
| 1.00 | 1.00 | 1.70 | Undisturbed | | 1 | A |
| 1.15 | 2.17 | 1.53 | Undisturbed | | 1 | A |
| 0.74 | 1.59 | 2.00 | Undisturbed | | 1 | A |
| 1.17 | 1.95 | 1.51 | Undisturbed | | 1 | A |
| 1.53 | 0.48 | 1.10 | Undisturbed | | 1 | A |
| 1.13 | 2.70 | 1.56 | Undisturbed | | 1 | A |
| 1.14 | 2.28 | 1.54 | Undisturbed | | 1 | A |
| 1.06 | 1.88 | 1.63 | Undisturbed | | 1 | A |
| 1.08 | 1.18 | 1.61 | Undisturbed | | 1 | A |
| 1.15 | 0.78 | 1.53 | Undisturbed | | 1 | A |
| 1.34 | 1.79 | 1.31 | Undisturbed | | 1 | A |
| 1.59 | 1.04 | 1.02 | Undisturbed | | 1 | A |
| 1.72 | 1.04 | 0.88 | Undisturbed | | 1 | A |
| 1.19 | 0.30 | 1.49 | Undisturbed | | 1 | A |
| 1.32 | 0.30 | 1.33 | Undisturbed | | 1 | A |
| 1.24 | 1.74 | 1.42 | Undisturbed | | 1 | A |
| 0.98 | 2.16 | 1.73 | Undisturbed | | 1 | A |
| 1.15 | 3.00 | 1.53 | Undisturbed | | 1 | A |
| 0.43 | 1.75 | 2.36 | Undisturbed | | 1 | A |
| 0.97 | 1.42 | 1.74 | Undisturbed | | 1 | A |
| 1.12 | 2.05 | 1.56 | Undisturbed | | 1 | A |
| 1.13 | 1.04 | 1.55 | Undisturbed | | 1 | A |
| 1.13 | 2.19 | 1.55 | Undisturbed | | 1 | A |
| 0.99 | 1.42 | 1.71 | Undisturbed | | 1 | A |
| 1.00 | 0.30 | 1.70 | Undisturbed | | 1 | A |
| 1.22 | 1.54 | 1.45 | Undisturbed | | 1 | A |
| 1.00 | 2.08 | 1.70 | Undisturbed | | 1 | A |
| 1.06 | 2.36 | 1.64 | Undisturbed | | 1 | A |
| 1.27 | 0.48 | 1.39 | Undisturbed | | 1 | A |
| 1.14 | 1.49 | 1.54 | Undisturbed | | 1 | A |
| 1.87 | 0.30 | 0.71 | Undisturbed | | 1 | A |
| 1.29 | 0.85 | 1.37 | Undisturbed | | 1 | A |
| 1.28 | 2.49 | 1.39 | Undisturbed | | 1 | A |
| 1.00 | 0.30 | 1.70 | Undisturbed | | 1 | A |
| 1.22 | 1.54 | 1.45 | Undisturbed | | 1 | A |
| 1.00 | 2.08 | 1.70 | Undisturbed | | 1 | A |
| 1.06 | 2.36 | 1.64 | Undisturbed | | 1 | A |
| 1.27 | 0.48 | 1.39 | Undisturbed | | 1 | A |
| 1.14 | 1.49 | 1.54 | Undisturbed | | 1 | A |
| 1.87 | 0.30 | 0.71 | Undisturbed | | 1 | A |
| 1.29 | 0.85 | 1.37 | Undisturbed | | 1 | A |
| 1.28 | 2.49 | 1.39 | Undisturbed | | 1 | A |
| 1.16 | 1.72 | 1.41 | 4 | | 2 | A |
| 1.30 | 1.00 | 1.67 | 4 | | 2 | A |
| 0.78 | 1.41 | 0.69 | 4 | | 2 | A |
| 1.17 | 1.49 | 1.43 | 4 | | 2 | A |
| 1.31 | 2.62 | 1.69 | 4 | | 2 | A |
| 1.33 | 1.76 | 1.73 | 4 | | 2 | A |
| 1.30 | 2.31 | 1.67 | 4 | | 2 | A |
| 1.24 | 2.03 | 1.56 | 4 | | 2 | A |
| 0.79 | 0.60 | 0.71 | 4 | | 2 | A |
| 1.52 | 3.02 | 2.08 | 4 | | 2 | A |
| 1.22 | 0.00 | 1.52 | 4 | | 2 | A |
| 1.17 | 1.53 | 1.43 | 4 | | 2 | A |
| 1.15 | 1.52 | 1.39 | 4 | | 2 | A |
| 1.13 | 0.00 | 1.35 | 4 | | 2 | A |
| 1.25 | 1.00 | 1.58 | 4 | | 2 | A |
| 0.90 | 1.83 | 0.92 | 4 | | 2 | A |
| 1.07 | 1.40 | 1.24 | 4 | | 2 | A |
| 1.41 | 0.48 | 1.88 | 4 | | 2 | A |
| 1.28 | 2.79 | 1.63 | 4 | | 2 | A |
| 1.56 | 3.05 | 2.16 | 4 | | 2 | A |
| 1.03 | 0.00 | 1.16 | 4 | | 2 | A |
| 1.06 | 0.00 | 1.22 | 4 | | 2 | A |
| 1.19 | 1.80 | 1.46 | 4 | | 2 | A |
| 1.28 | 2.47 | 1.63 | 4 | | 2 | A |
| 1.41 | 1.99 | 1.88 | 4 | | 2 | A |
| 1.06 | 1.75 | 1.22 | 4 | | 2 | A |
| 1.09 | 0.30 | 1.28 | 4 | | 2 | A |
| 1.07 | 1.18 | 1.24 | 4 | | 2 | A |
| 1.27 | 1.04 | 1.61 | 4 | | 2 | A |
| 1.44 | 2.74 | 1.93 | 4 | | 2 | A |
| 1.22 | 0.60 | 1.52 | 4 | | 2 | A |
| 1.27 | 2.23 | 1.61 | 4 | | 2 | A |
| 0.98 | 0.95 | 1.07 | 4 | | 2 | A |
| 1.22 | 1.93 | 1.52 | 4 | | 2 | A |
| 1.43 | 2.11 | 1.92 | 4 | | 2 | A |
| 0.89 | 1.83 | 0.90 | 4 | | 2 | A |
| 1.31 | 2.10 | 1.69 | 4 | | 2 | A |
| 1.46 | 0.00 | 1.97 | 4 | | 2 | A |
| 0.67 | 1.98 | 1.37 | 6 | | 2 | A |
| 1.44 | 2.32 | 1.57 | 6 | | 2 | A |
| 1.31 | 1.75 | 1.54 | 6 | | 2 | A |
| 1.60 | 2.66 | 1.61 | 6 | | 2 | A |
| 1.29 | 0.95 | 1.53 | 6 | | 2 | A |
| 1.15 | 0.70 | 1.50 | 6 | | 2 | A |
| 1.19 | 0.70 | 1.51 | 6 | | 2 | A |
| 1.33 | 1.11 | 1.54 | 6 | | 2 | A |
| 1.24 | 1.52 | 1.52 | 6 | | 2 | A |
| 1.11 | 0.30 | 1.49 | 6 | | 2 | A |
| 1.08 | 1.43 | 1.48 | 6 | | 2 | A |
| 1.15 | 1.18 | 1.50 | 6 | | 2 | A |
| 1.20 | 1.00 | 1.51 | 6 | | 2 | A |
| 0.87 | 0.90 | 1.42 | 6 | | 2 | A |
| 1.13 | 1.34 | 1.49 | 6 | | 2 | A |
| 1.01 | 1.57 | 1.46 | 6 | | 2 | A |
| 1.03 | 2.36 | 1.47 | 6 | | 2 | A |
| 1.25 | 3.00 | 1.52 | 6 | | 2 | A |
| 1.09 | 2.90 | 1.48 | 6 | | 2 | A |
| 1.18 | 2.21 | 1.50 | 6 | | 2 | A |
| 1.13 | 0.48 | 1.49 | 6 | | 2 | A |
| 1.33 | 1.91 | 1.54 | 6 | | 2 | A |
| 1.06 | 1.43 | 1.47 | 6 | | 2 | A |
| 1.28 | 2.40 | 1.53 | 6 | | 2 | A |
| 1.17 | 1.04 | 1.50 | 6 | | 2 | A |
| 1.04 | 2.04 | 1.47 | 6 | | 2 | A |
| 1.23 | 0.60 | 1.52 | 6 | | 2 | A |
| 1.10 | 2.03 | 1.48 | 6 | | 2 | A |
| 1.35 | 1.43 | 1.55 | 6 | | 2 | A |
| 1.40 | 0.30 | 1.56 | 6 | | 2 | A |
| 1.08 | 1.04 | 1.48 | 6 | | 2 | A |
| 1.00 | 1.46 | 1.46 | 6 | | 2 | A |
| 1.46 | 1.40 | 1.58 | 6 | | 2 | A |
| 1.07 | 1.65 | 1.48 | 6 | | 2 | A |
| 1.20 | 1.26 | 1.51 | 6 | | 2 | A |
| 1.05 | 1.54 | 1.47 | 6 | | 2 | A |
| 1.13 | 1.72 | 1.49 | 6 | | 2 | A |
| 1.29 | 2.47 | 1.79 | 10 | | 2 | A |
| 1.03 | 1.81 | 1.71 | 10 | | 2 | A |
| 1.24 | 2.73 | 1.77 | 10 | | 2 | A |
| 1.34 | 0.30 | 1.80 | 10 | | 2 | A |
| 0.70 | 0.70 | 1.62 | 10 | | 2 | A |
| 1.31 | 0.70 | 1.79 | 10 | | 2 | A |
| 1.37 | 1.88 | 1.81 | 10 | | 2 | A |
| 1.32 | 2.79 | 1.80 | 10 | | 2 | A |
| 1.19 | 2.76 | 1.76 | 10 | | 2 | A |
| 0.89 | 0.00 | 1.67 | 10 | | 2 | A |
| 1.06 | 2.55 | 1.72 | 10 | | 2 | A |
| 0.85 | 0.00 | 1.66 | 10 | | 2 | A |
| 0.95 | 0.48 | 1.69 | 10 | | 2 | A |
| 1.43 | 1.97 | 1.83 | 10 | | 2 | A |
| 1.12 | 2.45 | 1.74 | 10 | | 2 | A |
| 1.13 | 2.05 | 1.74 | 10 | | 2 | A |
| 1.24 | 2.27 | 1.77 | 10 | | 2 | A |
| 1.04 | 2.33 | 1.71 | 10 | | 2 | A |
| 1.17 | 2.47 | 1.75 | 10 | | 2 | A |
| 1.16 | 2.72 | 1.75 | 10 | | 2 | A |
| 1.35 | 1.83 | 1.80 | 10 | | 2 | A |
| 1.00 | 2.63 | 1.70 | 10 | | 2 | A |
| 1.17 | 2.48 | 1.75 | 10 | | 2 | A |
| 1.16 | 2.57 | 1.75 | 10 | | 2 | A |
| 1.19 | 1.26 | 1.76 | 10 | | 2 | A |
| 1.17 | 2.23 | 1.75 | 10 | | 2 | A |
| 1.22 | 1.83 | 1.77 | 10 | | 2 | A |
| 1.17 | 2.87 | 1.75 | 10 | | 2 | A |
| 1.06 | 1.20 | 1.72 | 10 | | 2 | A |
| 0.88 | 2.51 | 1.67 | 10 | | 2 | A |
| 1.09 | 2.00 | 1.73 | 10 | | 2 | A |
| 1.33 | 0.60 | 1.80 | 10 | | 2 | A |
| 1.13 | 2.09 | 1.74 | 10 | | 2 | A |
| 1.25 | 2.31 | 1.77 | 10 | | 2 | A |
| 1.17 | 1.11 | 1.75 | 10 | | 2 | A |
| 1.26 | 0.48 | 1.78 | 10 | | 2 | A |
| 1.59 | 1.18 | 1.87 | 10 | | 2 | A |
| 1.28 | 2.25 | 1.78 | 10 | | 2 | A |
| 1.22 | 2.21 | 1.77 | 10 | | 2 | A |
| 0.94 | 2.38 | 1.69 | 10 | | 2 | A |
| 1.64 | 1.04 | 1.89 | 10 | | 2 | A |
| 1.10 | 1.83 | 1.73 | 10 | | 2 | A |
| 1.25 | 0.78 | 1.77 | 10 | | 2 | A |
| 0.61 | 0.85 | 1.59 | 10 | | 2 | A |
| 1.19 | 2.67 | 1.76 | 10 | | 2 | A |
| 1.20 | 1.91 | 1.76 | 10 | | 2 | A |
| 1.05 | 1.49 | 1.72 | 10 | | 2 | A |
| 1.39 | 0.00 | 1.82 | 10 | | 2 | A |
| 0.89 | 2.85 | 2.25 | 13 | | 2 | A |
| 1.05 | 2.06 | 2.07 | 13 | | 2 | A |
| 0.79 | 1.32 | 2.37 | 13 | | 2 | A |
| 0.91 | 2.69 | 2.23 | 13 | | 2 | A |
| 1.34 | 1.62 | 1.73 | 13 | | 2 | A |
| 1.59 | 1.86 | 1.44 | 13 | | 2 | A |
| 1.19 | 2.19 | 1.90 | 13 | | 2 | A |
| 1.04 | 2.76 | 2.08 | 13 | | 2 | A |
| 1.12 | 2.56 | 1.99 | 13 | | 2 | A |
| 0.94 | 2.11 | 2.19 | 13 | | 2 | A |
| 1.35 | 0.85 | 1.72 | 13 | | 2 | A |
| 1.10 | 2.03 | 2.01 | 13 | | 2 | A |
| 1.12 | 0.90 | 1.99 | 13 | | 2 | A |
| 1.32 | 1.89 | 1.75 | 13 | | 2 | A |
| 0.89 | 2.30 | 2.25 | 13 | | 2 | A |
| 1.14 | 1.66 | 1.96 | 13 | | 2 | A |
| 0.67 | 2.58 | 2.50 | 13 | | 2 | A |
| 0.98 | 1.90 | 2.15 | 13 | | 2 | A |
| 1.17 | 0.60 | 1.93 | 13 | | 2 | A |
| 1.24 | 2.81 | 1.85 | 13 | | 2 | A |
| 1.14 | 2.29 | 1.96 | 13 | | 2 | A |
| 1.25 | 2.55 | 1.84 | 13 | | 2 | A |
| 1.28 | 2.53 | 1.80 | 13 | | 2 | A |
| 1.16 | 2.15 | 1.94 | 13 | | 2 | A |
| 1.10 | 1.40 | 2.01 | 13 | | 2 | A |
| 1.47 | 2.35 | 1.58 | 13 | | 2 | A |
| 1.10 | 2.68 | 2.01 | 13 | | 2 | A |
| 1.22 | 1.76 | 1.87 | 13 | | 2 | A |
| 1.24 | 1.88 | 1.85 | 13 | | 2 | A |
| 1.52 | 0.00 | 1.52 | 13 | | 2 | A |
| 1.03 | 2.77 | 2.09 | 13 | | 2 | A |
| 1.21 | 2.27 | 1.88 | 13 | | 2 | A |
| 1.14 | 1.43 | 1.96 | 13 | | 2 | A |
| 1.37 | 1.86 | 1.70 | 13 | | 2 | A |
| 1.13 | 1.80 | 1.97 | 13 | | 2 | A |
| 1.16 | 2.59 | 1.94 | 13 | | 2 | A |
| 1.41 | 1.11 | 1.65 | 13 | | 2 | A |
| 1.02 | 1.04 | 2.10 | 13 | | 2 | A |
| 1.61 | 1.30 | 1.42 | 13 | | 2 | A |
| 1.23 | 1.84 | 1.86 | 13 | | 2 | A |
| 1.19 | 2.93 | 1.90 | 13 | | 2 | A |
| 1.21 | 1.96 | 1.88 | 13 | | 2 | A |
| 0.98 | 1.91 | 2.15 | 13 | | 2 | A |
| 1.40 | 1.77 | 1.66 | 13 | | 2 | A |
| 1.00 | 1.61 | 2.12 | 13 | | 2 | A |
| 1.25 | 1.93 | 1.84 | 13 | | 2 | A |
| 1.31 | 1.45 | 1.77 | 13 | | 2 | A |
| 1.08 | 1.43 | 2.03 | 13 | | 2 | A |
| 1.10 | 2.79 | 2.01 | 13 | | 2 | A |
| 1.05 | 2.16 | 2.07 | 13 | | 2 | A |
| 1.28 | 1.45 | 1.80 | 13 | | 2 | A |
| 1.65 | 1.18 | 1.37 | 13 | | 2 | A |
| 1.58 | 1.65 | 1.46 | 13 | | 2 | A |
| 0.90 | 2.52 | 1.86 | Undisturbed | | 2 | A |
| 1.09 | 1.26 | 1.59 | Undisturbed | | 2 | A |
| 1.00 | 2.31 | 1.72 | Undisturbed | | 2 | A |
| 0.64 | 1.89 | 2.24 | Undisturbed | | 2 | A |
| 1.22 | 0.95 | 1.40 | Undisturbed | | 2 | A |
| 1.06 | 0.30 | 1.63 | Undisturbed | | 2 | A |
| 1.39 | 1.96 | 1.15 | Undisturbed | | 2 | A |
| 0.96 | 2.62 | 1.78 | Undisturbed | | 2 | A |
| 1.18 | 0.00 | 1.46 | Undisturbed | | 2 | A |
| 1.30 | 0.00 | 1.28 | Undisturbed | | 2 | A |
| 1.13 | 0.48 | 1.53 | Undisturbed | | 2 | A |
| 1.41 | 1.41 | 1.12 | Undisturbed | | 2 | A |
| 1.27 | 1.83 | 1.32 | Undisturbed | | 2 | A |
| 1.17 | 1.04 | 1.47 | Undisturbed | | 2 | A |
| 0.95 | 2.64 | 1.79 | Undisturbed | | 2 | A |
| 0.99 | 2.63 | 1.73 | Undisturbed | | 2 | A |
| 0.98 | 1.46 | 1.75 | Undisturbed | | 2 | A |
| 0.98 | 0.30 | 1.75 | Undisturbed | | 2 | A |
| 1.70 | 0.30 | 0.70 | Undisturbed | | 2 | A |
| 1.24 | 0.30 | 1.37 | Undisturbed | | 2 | A |
| 1.24 | 2.19 | 1.37 | Undisturbed | | 2 | A |
| 1.10 | 2.15 | 1.57 | Undisturbed | | 2 | A |
| 1.23 | 1.94 | 1.38 | Undisturbed | | 2 | A |
| 1.18 | 2.18 | 1.46 | Undisturbed | | 2 | A |
| 0.56 | 2.77 | 2.36 | Undisturbed | | 2 | A |
| 1.11 | 1.18 | 1.56 | Undisturbed | | 2 | A |
| 0.95 | 2.51 | 1.79 | Undisturbed | | 2 | A |
| 1.20 | 2.20 | 1.43 | Undisturbed | | 2 | A |
| 0.78 | 2.54 | 2.04 | Undisturbed | | 2 | A |
| 1.18 | 2.01 | 1.46 | Undisturbed | | 2 | A |
| 1.24 | 2.55 | 1.37 | Undisturbed | | 2 | A |
| 1.29 | 2.26 | 1.30 | Undisturbed | | 2 | A |
| 1.35 | 2.49 | 1.21 | Undisturbed | | 2 | A |
| 1.03 | 0.00 | 1.67 | Undisturbed | | 2 | A |
| 1.12 | 0.00 | 1.54 | Undisturbed | | 2 | A |
| 0.88 | 2.37 | 1.89 | Undisturbed | | 2 | A |
| 1.13 | 1.59 | 1.53 | Undisturbed | | 2 | A |
| 1.26 | 2.12 | 1.34 | Undisturbed | | 2 | A |
| 1.00 | 0.30 | 1.72 | Undisturbed | | 2 | A |
| 1.15 | 1.99 | 1.50 | Undisturbed | | 2 | A |
| 0.74 | 1.86 | 2.10 | Undisturbed | | 2 | A |
| 1.17 | 1.88 | 1.47 | Undisturbed | | 2 | A |
| 1.53 | 0.00 | 0.95 | Undisturbed | | 2 | A |
| 1.13 | 2.74 | 1.53 | Undisturbed | | 2 | A |
| 1.14 | 2.60 | 1.51 | Undisturbed | | 2 | A |
| 1.06 | 0.00 | 1.63 | Undisturbed | | 2 | A |
| 1.08 | 1.60 | 1.60 | Undisturbed | | 2 | A |
| 1.34 | 1.81 | 1.22 | Undisturbed | | 2 | A |
| 1.59 | 0.30 | 0.86 | Undisturbed | | 2 | A |
| 1.72 | 0.48 | 0.67 | Undisturbed | | 2 | A |
| 1.19 | 0.00 | 1.44 | Undisturbed | | 2 | A |
| 1.32 | 0.78 | 1.25 | Undisturbed | | 2 | A |
| 1.24 | 2.18 | 1.37 | Undisturbed | | 2 | A |
| 0.98 | 2.00 | 1.75 | Undisturbed | | 2 | A |
| 1.15 | 2.93 | 1.50 | Undisturbed | | 2 | A |
| 0.43 | 2.10 | 2.55 | Undisturbed | | 2 | A |
| 0.97 | 1.00 | 1.76 | Undisturbed | | 2 | A |
| 1.13 | 1.34 | 1.53 | Undisturbed | | 2 | A |
| 1.13 | 1.92 | 1.53 | Undisturbed | | 2 | A |
| 0.99 | 2.00 | 1.73 | Undisturbed | | 2 | A |
| 1.00 | 0.00 | 1.72 | Undisturbed | | 2 | A |
| 1.22 | 2.20 | 1.40 | Undisturbed | | 2 | A |
| 1.00 | 1.95 | 1.72 | Undisturbed | | 2 | A |
| 1.06 | 2.14 | 1.63 | Undisturbed | | 2 | A |
| 1.27 | 0.30 | 1.32 | Undisturbed | | 2 | A |
| 1.14 | 1.20 | 1.51 | Undisturbed | | 2 | A |
| 1.87 | 0.00 | 0.45 | Undisturbed | | 2 | A |
| 1.28 | 2.27 | 1.31 | Undisturbed | | 2 | A |
| -0.10 | 1.30 | 1.43 | 4 | | 1 | Pro |
| 0.44 | 2.12 | 1.99 | 4 | | 1 | Pro |
| -0.17 | 1.59 | 1.36 | 4 | | 1 | Pro |
| -0.53 | 0.95 | 0.99 | 4 | | 1 | Pro |
| 0.21 | 2.84 | 1.75 | 4 | | 1 | Pro |
| 0.08 | 1.30 | 1.62 | 4 | | 1 | Pro |
| 0.26 | 1.69 | 1.80 | 4 | | 1 | Pro |
| -0.59 | 0.78 | 0.92 | 4 | | 1 | Pro |
| 0.00 | 0.30 | 1.53 | 4 | | 1 | Pro |
| 0.60 | 1.79 | 2.15 | 4 | | 1 | Pro |
| -0.51 | 1.11 | 1.01 | 4 | | 1 | Pro |
| 0.79 | 1.79 | 2.35 | 4 | | 1 | Pro |
| -0.69 | 0.00 | 0.82 | 4 | | 1 | Pro |
| 0.12 | 2.39 | 1.65 | 4 | | 1 | Pro |
| 0.24 | 3.10 | 1.78 | 4 | | 1 | Pro |
| -0.61 | 1.90 | 0.90 | 4 | | 1 | Pro |
| -0.17 | 0.48 | 1.35 | 4 | | 1 | Pro |
| 0.30 | 1.32 | 1.84 | 4 | | 1 | Pro |
| 0.78 | 1.95 | 2.34 | 4 | | 1 | Pro |
| 0.36 | 2.29 | 1.91 | 4 | | 1 | Pro |
| 0.02 | 1.96 | 1.55 | 4 | | 1 | Pro |
| -0.27 | 0.70 | 1.25 | 4 | | 1 | Pro |
| 0.01 | 1.26 | 1.55 | 4 | | 1 | Pro |
| 0.20 | 1.08 | 1.74 | 4 | | 1 | Pro |
| 0.12 | 2.89 | 1.66 | 4 | | 1 | Pro |
| -0.61 | 1.08 | 0.89 | 4 | | 1 | Pro |
| -0.35 | 1.18 | 1.16 | 4 | | 1 | Pro |
| 0.30 | 1.89 | 1.85 | 4 | | 1 | Pro |
| 0.69 | 1.69 | 2.25 | 4 | | 1 | Pro |
| 0.41 | 2.56 | 1.96 | 4 | | 1 | Pro |
| 0.42 | 1.99 | 1.96 | 4 | | 1 | Pro |
| -0.64 | 0.30 | 0.87 | 4 | | 1 | Pro |
| -0.07 | 1.72 | 1.46 | 4 | | 1 | Pro |
| 0.05 | 2.05 | 1.58 | 4 | | 1 | Pro |
| 0.63 | 2.04 | 2.19 | 4 | | 1 | Pro |
| 0.24 | 2.62 | 1.46 | 6 | | 1 | Pro |
| 0.56 | 1.00 | 1.58 | 6 | | 1 | Pro |
| -0.03 | 0.60 | 1.36 | 6 | | 1 | Pro |
| 0.42 | 0.48 | 1.52 | 6 | | 1 | Pro |
| -0.02 | 0.30 | 1.37 | 6 | | 1 | Pro |
| -0.06 | 0.30 | 1.35 | 6 | | 1 | Pro |
| 0.76 | 1.88 | 1.65 | 6 | | 1 | Pro |
| 0.32 | 1.84 | 1.49 | 6 | | 1 | Pro |
| 0.43 | 0.70 | 1.53 | 6 | | 1 | Pro |
| 0.01 | 1.68 | 1.38 | 6 | | 1 | Pro |
| -0.66 | 1.40 | 1.14 | 6 | | 1 | Pro |
| 0.13 | 1.04 | 1.42 | 6 | | 1 | Pro |
| 0.01 | 0.00 | 1.38 | 6 | | 1 | Pro |
| 0.40 | 2.26 | 1.52 | 6 | | 1 | Pro |
| 0.11 | 3.01 | 1.41 | 6 | | 1 | Pro |
| 0.46 | 1.95 | 1.54 | 6 | | 1 | Pro |
| -0.30 | 2.02 | 1.26 | 6 | | 1 | Pro |
| 0.44 | 1.99 | 1.53 | 6 | | 1 | Pro |
| -0.06 | 2.57 | 1.35 | 6 | | 1 | Pro |
| 0.07 | 1.60 | 1.40 | 6 | | 1 | Pro |
| 0.18 | 2.02 | 1.44 | 6 | | 1 | Pro |
| -0.10 | 0.00 | 1.34 | 6 | | 1 | Pro |
| -0.74 | 1.08 | 1.10 | 6 | | 1 | Pro |
| 0.39 | 1.89 | 1.51 | 6 | | 1 | Pro |
| 0.33 | 1.20 | 1.49 | 6 | | 1 | Pro |
| -0.06 | 1.18 | 1.35 | 6 | | 1 | Pro |
| -0.35 | 1.38 | 1.25 | 6 | | 1 | Pro |
| -0.65 | 1.71 | 1.14 | 6 | | 1 | Pro |
| -0.83 | 0.78 | 1.07 | 6 | | 1 | Pro |
| 0.07 | 1.00 | 1.40 | 6 | | 1 | Pro |
| 0.45 | 2.32 | 1.54 | 6 | | 1 | Pro |
| -0.30 | 1.53 | 1.27 | 6 | | 1 | Pro |
| 0.33 | 1.38 | 1.49 | 6 | | 1 | Pro |
| 0.35 | 0.70 | 1.50 | 6 | | 1 | Pro |
| 0.23 | 1.59 | 1.46 | 6 | | 1 | Pro |
| -0.91 | 2.20 | 1.67 | 10 | | 1 | Pro |
| -0.38 | 0.90 | 1.52 | 10 | | 1 | Pro |
| -0.86 | 1.66 | 1.65 | 10 | | 1 | Pro |
| -0.74 | 1.48 | 1.62 | 10 | | 1 | Pro |
| -0.83 | 1.52 | 1.64 | 10 | | 1 | Pro |
| -0.15 | 1.20 | 1.46 | 10 | | 1 | Pro |
| 0.02 | 1.80 | 1.42 | 10 | | 1 | Pro |
| -0.38 | 2.16 | 1.52 | 10 | | 1 | Pro |
| -0.16 | 0.30 | 1.46 | 10 | | 1 | Pro |
| -0.82 | 1.68 | 1.64 | 10 | | 1 | Pro |
| 0.14 | 0.60 | 1.38 | 10 | | 1 | Pro |
| 0.43 | 0.00 | 1.31 | 10 | | 1 | Pro |
| -0.72 | 1.43 | 1.62 | 10 | | 1 | Pro |
| -0.14 | 2.05 | 1.46 | 10 | | 1 | Pro |
| 0.09 | 1.94 | 1.40 | 10 | | 1 | Pro |
| -0.94 | 1.58 | 1.67 | 10 | | 1 | Pro |
| -0.07 | 1.20 | 1.44 | 10 | | 1 | Pro |
| -0.91 | 2.34 | 1.67 | 10 | | 1 | Pro |
| -0.24 | 1.81 | 1.49 | 10 | | 1 | Pro |
| -0.99 | 0.95 | 1.69 | 10 | | 1 | Pro |
| -1.21 | 1.92 | 1.75 | 10 | | 1 | Pro |
| -0.42 | 2.47 | 1.53 | 10 | | 1 | Pro |
| -0.09 | 1.04 | 1.45 | 10 | | 1 | Pro |
| -0.67 | 1.98 | 1.60 | 10 | | 1 | Pro |
| -0.39 | 2.11 | 1.53 | 10 | | 1 | Pro |
| 0.14 | 2.11 | 1.38 | 10 | | 1 | Pro |
| -0.12 | 2.50 | 1.45 | 10 | | 1 | Pro |
| 0.26 | 1.32 | 1.35 | 10 | | 1 | Pro |
| -0.66 | 1.87 | 1.60 | 10 | | 1 | Pro |
| -1.11 | 0.48 | 1.72 | 10 | | 1 | Pro |
| -0.11 | 3.16 | 1.45 | 10 | | 1 | Pro |
| -0.45 | 2.22 | 1.54 | 10 | | 1 | Pro |
| -0.41 | 0.00 | 1.53 | 10 | | 1 | Pro |
| -0.48 | 2.52 | 1.55 | 10 | | 1 | Pro |
| -0.32 | 1.00 | 1.51 | 10 | | 1 | Pro |
| -0.33 | 1.08 | 1.51 | 10 | | 1 | Pro |
| -0.74 | 1.42 | 1.62 | 10 | | 1 | Pro |
| -0.26 | 1.30 | 1.49 | 10 | | 1 | Pro |
| -0.80 | 1.36 | 1.64 | 10 | | 1 | Pro |
| -0.34 | 1.60 | 1.51 | 10 | | 1 | Pro |
| -0.49 | 2.47 | 1.55 | 10 | | 1 | Pro |
| 0.26 | 0.30 | 1.35 | 10 | | 1 | Pro |
| -1.10 | 1.04 | 1.72 | 10 | | 1 | Pro |
| -0.89 | 2.56 | 1.82 | 13 | | 1 | Pro |
| 0.32 | 1.62 | 1.51 | 13 | | 1 | Pro |
| -0.82 | 1.08 | 1.81 | 13 | | 1 | Pro |
| -0.66 | 1.96 | 1.76 | 13 | | 1 | Pro |
| -0.07 | 0.00 | 1.61 | 13 | | 1 | Pro |
| -0.61 | 1.40 | 1.75 | 13 | | 1 | Pro |
| -0.47 | 1.94 | 1.71 | 13 | | 1 | Pro |
| 0.19 | 1.72 | 1.54 | 13 | | 1 | Pro |
| -0.89 | 2.81 | 1.82 | 13 | | 1 | Pro |
| -0.73 | 2.35 | 1.78 | 13 | | 1 | Pro |
| -0.82 | 0.95 | 1.80 | 13 | | 1 | Pro |
| -0.38 | 2.08 | 1.69 | 13 | | 1 | Pro |
| -0.74 | 0.60 | 1.78 | 13 | | 1 | Pro |
| -0.35 | 1.20 | 1.68 | 13 | | 1 | Pro |
| -1.05 | 2.42 | 1.87 | 13 | | 1 | Pro |
| -0.56 | 1.87 | 1.74 | 13 | | 1 | Pro |
| -0.61 | 2.11 | 1.75 | 13 | | 1 | Pro |
| -0.50 | 2.30 | 1.72 | 13 | | 1 | Pro |
| -0.56 | 1.74 | 1.74 | 13 | | 1 | Pro |
| 0.40 | 2.78 | 1.49 | 13 | | 1 | Pro |
| 0.26 | 2.06 | 1.52 | 13 | | 1 | Pro |
| -0.25 | 2.41 | 1.66 | 13 | | 1 | Pro |
| -0.48 | 2.38 | 1.72 | 13 | | 1 | Pro |
| -0.08 | 1.98 | 1.61 | 13 | | 1 | Pro |
| -0.01 | 0.30 | 1.59 | 13 | | 1 | Pro |
| -0.59 | 2.65 | 1.75 | 13 | | 1 | Pro |
| -0.33 | 2.64 | 1.68 | 13 | | 1 | Pro |
| -0.68 | 1.67 | 1.77 | 13 | | 1 | Pro |
| -0.88 | 1.72 | 1.82 | 13 | | 1 | Pro |
| -0.15 | 2.47 | 1.63 | 13 | | 1 | Pro |
| -0.03 | 2.09 | 1.60 | 13 | | 1 | Pro |
| -0.24 | 0.48 | 1.65 | 13 | | 1 | Pro |
| -0.45 | 2.55 | 1.71 | 13 | | 1 | Pro |
| 0.01 | 0.00 | 1.59 | 13 | | 1 | Pro |
| 0.26 | 1.00 | 1.52 | 13 | | 1 | Pro |
| 0.43 | 1.70 | 1.48 | 13 | | 1 | Pro |
| -0.16 | 2.95 | 1.63 | 13 | | 1 | Pro |
| -0.56 | 1.30 | 1.74 | 13 | | 1 | Pro |
| -1.21 | 1.74 | 1.91 | 13 | | 1 | Pro |
| 0.11 | 0.60 | 1.56 | 13 | | 1 | Pro |
| -0.64 | 1.91 | 1.76 | 13 | | 1 | Pro |
| -0.70 | 1.93 | 1.77 | 13 | | 1 | Pro |
| 0.34 | 1.30 | 1.50 | 13 | | 1 | Pro |
| -0.14 | 1.48 | 1.63 | 13 | | 1 | Pro |
| -0.04 | 2.20 | 1.60 | 13 | | 1 | Pro |
| -0.25 | 1.48 | 1.66 | 13 | | 1 | Pro |
| 0.01 | 0.60 | 1.59 | 13 | | 1 | Pro |
| -0.84 | 0.48 | 1.81 | 13 | | 1 | Pro |
| -0.84 | 1.04 | 1.81 | 13 | | 1 | Pro |
| -0.74 | 2.78 | 1.59 | Undisturbed | | 1 | Pro |
| -0.89 | 2.12 | 1.59 | Undisturbed | | 1 | Pro |
| -0.40 | 1.30 | 1.58 | Undisturbed | | 1 | Pro |
| -0.76 | 0.78 | 1.59 | Undisturbed | | 1 | Pro |
| -0.85 | 1.67 | 1.59 | Undisturbed | | 1 | Pro |
| -0.51 | 2.49 | 1.59 | Undisturbed | | 1 | Pro |
| -0.68 | 1.26 | 1.59 | Undisturbed | | 1 | Pro |
| -0.68 | 0.00 | 1.59 | Undisturbed | | 1 | Pro |
| -0.59 | 1.78 | 1.59 | Undisturbed | | 1 | Pro |
| -0.24 | 1.43 | 1.58 | Undisturbed | | 1 | Pro |
| -0.38 | 2.82 | 1.58 | Undisturbed | | 1 | Pro |
| -0.59 | 2.49 | 1.59 | Undisturbed | | 1 | Pro |
| -0.55 | 1.11 | 1.59 | Undisturbed | | 1 | Pro |
| -0.42 | 1.42 | 1.58 | Undisturbed | | 1 | Pro |
| -0.92 | 0.90 | 1.59 | Undisturbed | | 1 | Pro |
| -0.54 | 0.48 | 1.59 | Undisturbed | | 1 | Pro |
| -1.14 | 2.49 | 1.60 | Undisturbed | | 1 | Pro |
| -0.97 | 1.77 | 1.59 | Undisturbed | | 1 | Pro |
| -0.53 | 1.71 | 1.59 | Undisturbed | | 1 | Pro |
| -0.19 | 1.46 | 1.58 | Undisturbed | | 1 | Pro |
| -0.97 | 2.49 | 1.59 | Undisturbed | | 1 | Pro |
| -0.72 | 1.11 | 1.59 | Undisturbed | | 1 | Pro |
| -0.05 | 2.49 | 1.58 | Undisturbed | | 1 | Pro |
| -0.56 | 1.81 | 1.59 | Undisturbed | | 1 | Pro |
| -0.68 | 2.59 | 1.59 | Undisturbed | | 1 | Pro |
| -0.62 | 2.49 | 1.59 | Undisturbed | | 1 | Pro |
| 0.25 | 2.42 | 1.57 | Undisturbed | | 1 | Pro |
| -0.36 | 2.21 | 1.58 | Undisturbed | | 1 | Pro |
| -0.22 | 0.00 | 1.58 | Undisturbed | | 1 | Pro |
| -0.59 | 0.30 | 1.59 | Undisturbed | | 1 | Pro |
| -0.73 | 1.93 | 1.59 | Undisturbed | | 1 | Pro |
| 0.48 | 0.00 | 1.57 | Undisturbed | | 1 | Pro |
| -0.69 | 2.30 | 1.59 | Undisturbed | | 1 | Pro |
| -0.30 | 1.61 | 1.58 | Undisturbed | | 1 | Pro |
| -0.27 | 2.47 | 1.58 | Undisturbed | | 1 | Pro |
| 0.03 | 2.17 | 1.58 | Undisturbed | | 1 | Pro |
| -1.09 | 1.59 | 1.60 | Undisturbed | | 1 | Pro |
| -0.97 | 1.95 | 1.59 | Undisturbed | | 1 | Pro |
| -0.49 | 2.70 | 1.59 | Undisturbed | | 1 | Pro |
| -0.34 | 2.28 | 1.58 | Undisturbed | | 1 | Pro |
| -0.46 | 1.88 | 1.59 | Undisturbed | | 1 | Pro |
| 0.16 | 1.18 | 1.57 | Undisturbed | | 1 | Pro |
| -0.20 | 1.04 | 1.58 | Undisturbed | | 1 | Pro |
| -0.74 | 1.04 | 1.59 | Undisturbed | | 1 | Pro |
| -1.11 | 0.30 | 1.60 | Undisturbed | | 1 | Pro |
| -0.89 | 0.30 | 1.59 | Undisturbed | | 1 | Pro |
| -0.21 | 1.74 | 1.58 | Undisturbed | | 1 | Pro |
| -0.57 | 2.16 | 1.59 | Undisturbed | | 1 | Pro |
| -0.54 | 3.00 | 1.59 | Undisturbed | | 1 | Pro |
| -0.45 | 1.75 | 1.58 | Undisturbed | | 1 | Pro |
| -0.91 | 1.42 | 1.59 | Undisturbed | | 1 | Pro |
| -0.57 | 1.04 | 1.59 | Undisturbed | | 1 | Pro |
| -0.40 | 2.19 | 1.58 | Undisturbed | | 1 | Pro |
| -0.46 | 1.42 | 1.59 | Undisturbed | | 1 | Pro |
| -0.44 | 0.30 | 1.58 | Undisturbed | | 1 | Pro |
| -0.91 | 1.54 | 1.59 | Undisturbed | | 1 | Pro |
| -0.32 | 0.00 | 1.58 | Undisturbed | | 1 | Pro |
| -0.25 | 2.08 | 1.58 | Undisturbed | | 1 | Pro |
| -0.56 | 2.36 | 1.59 | Undisturbed | | 1 | Pro |
| 0.08 | 0.48 | 1.58 | Undisturbed | | 1 | Pro |
| -0.96 | 1.49 | 1.59 | Undisturbed | | 1 | Pro |
| -1.08 | 0.30 | 1.60 | Undisturbed | | 1 | Pro |
| -0.85 | 0.85 | 1.59 | Undisturbed | | 1 | Pro |
| -0.39 | 2.49 | 1.58 | Undisturbed | | 1 | Pro |
| -0.10 | 1.00 | 1.06 | 4 | | 2 | Pro |
| 0.44 | 2.08 | 1.75 | 4 | | 2 | Pro |
| -0.17 | 1.61 | 0.98 | 4 | | 2 | Pro |
| 0.46 | 1.76 | 1.77 | 4 | | 2 | Pro |
| -0.53 | 0.00 | 0.52 | 4 | | 2 | Pro |
| 0.21 | 3.02 | 1.46 | 4 | | 2 | Pro |
| 0.79 | 1.32 | 2.20 | 4 | | 2 | Pro |
| 0.26 | 1.53 | 1.52 | 4 | | 2 | Pro |
| -0.59 | 0.48 | 0.45 | 4 | | 2 | Pro |
| 0.00 | 1.15 | 1.19 | 4 | | 2 | Pro |
| 0.60 | 1.70 | 1.95 | 4 | | 2 | Pro |
| -0.51 | 0.30 | 0.55 | 4 | | 2 | Pro |
| 0.74 | 1.40 | 2.13 | 4 | | 2 | Pro |
| -0.69 | 0.48 | 0.32 | 4 | | 2 | Pro |
| 0.12 | 1.32 | 1.34 | 4 | | 2 | Pro |
| 0.24 | 2.05 | 1.50 | 4 | | 2 | Pro |
| 0.39 | 1.88 | 1.69 | 4 | | 2 | Pro |
| -0.17 | 0.00 | 0.97 | 4 | | 2 | Pro |
| 0.30 | 1.23 | 1.58 | 4 | | 2 | Pro |
| 0.78 | 1.80 | 2.18 | 4 | | 2 | Pro |
| 0.61 | 1.99 | 1.98 | 4 | | 2 | Pro |
| 0.02 | 1.75 | 1.21 | 4 | | 2 | Pro |
| -0.27 | 0.30 | 0.85 | 4 | | 2 | Pro |
| 0.01 | 1.18 | 1.21 | 4 | | 2 | Pro |
| 0.20 | 1.04 | 1.45 | 4 | | 2 | Pro |
| 0.12 | 1.74 | 1.35 | 4 | | 2 | Pro |
| -0.40 | 0.60 | 0.68 | 4 | | 2 | Pro |
| 0.39 | 2.23 | 1.68 | 4 | | 2 | Pro |
| -0.35 | 0.95 | 0.74 | 4 | | 2 | Pro |
| 0.30 | 1.93 | 1.58 | 4 | | 2 | Pro |
| 0.69 | 1.83 | 2.07 | 4 | | 2 | Pro |
| 0.41 | 2.11 | 1.72 | 4 | | 2 | Pro |
| 0.52 | 2.10 | 1.86 | 4 | | 2 | Pro |
| -0.64 | 0.00 | 0.38 | 4 | | 2 | Pro |
| -0.07 | 1.98 | 1.21 | 6 | | 2 | Pro |
| 0.05 | 1.30 | 1.30 | 6 | | 2 | Pro |
| 0.63 | 1.75 | 1.71 | 6 | | 2 | Pro |
| 0.24 | 1.65 | 1.43 | 6 | | 2 | Pro |
| 0.56 | 1.46 | 1.66 | 6 | | 2 | Pro |
| 0.42 | 1.23 | 1.56 | 6 | | 2 | Pro |
| -0.46 | 0.00 | 0.94 | 6 | | 2 | Pro |
| -0.06 | 1.18 | 1.22 | 6 | | 2 | Pro |
| 0.76 | 1.52 | 1.80 | 6 | | 2 | Pro |
| 0.32 | 1.43 | 1.49 | 6 | | 2 | Pro |
| 0.43 | 1.18 | 1.57 | 6 | | 2 | Pro |
| 0.01 | 1.00 | 1.27 | 6 | | 2 | Pro |
| -0.66 | 0.30 | 0.80 | 6 | | 2 | Pro |
| 0.13 | 1.57 | 1.36 | 6 | | 2 | Pro |
| 0.40 | 1.43 | 1.54 | 6 | | 2 | Pro |
| 0.08 | 1.91 | 1.32 | 6 | | 2 | Pro |
| 0.46 | 1.76 | 1.59 | 6 | | 2 | Pro |
| -0.17 | 1.91 | 1.14 | 6 | | 2 | Pro |
| 0.44 | 1.43 | 1.58 | 6 | | 2 | Pro |
| -0.06 | 1.43 | 1.22 | 6 | | 2 | Pro |
| 0.07 | 1.04 | 1.31 | 6 | | 2 | Pro |
| 0.20 | 1.72 | 1.41 | 6 | | 2 | Pro |
| -0.10 | 1.15 | 1.19 | 6 | | 2 | Pro |
| -1.14 | 0.60 | 0.46 | 6 | | 2 | Pro |
| 0.41 | 1.73 | 1.55 | 6 | | 2 | Pro |
| 0.33 | 1.43 | 1.50 | 6 | | 2 | Pro |
| -0.35 | 0.30 | 1.01 | 6 | | 2 | Pro |
| -0.65 | 0.48 | 0.80 | 6 | | 2 | Pro |
| -0.83 | 0.30 | 0.68 | 6 | | 2 | Pro |
| 0.07 | 1.40 | 1.31 | 6 | | 2 | Pro |
| 0.45 | 1.65 | 1.58 | 6 | | 2 | Pro |
| -0.30 | 1.26 | 1.05 | 6 | | 2 | Pro |
| 0.33 | 1.54 | 1.50 | 6 | | 2 | Pro |
| 0.35 | 1.42 | 1.51 | 6 | | 2 | Pro |
| 0.23 | 1.72 | 1.42 | 6 | | 2 | Pro |
| -0.91 | 1.46 | 0.62 | 6 | | 2 | Pro |
| -0.38 | 1.81 | 1.68 | 10 | | 2 | Pro |
| 1.16 | 0.00 | 0.93 | 10 | | 2 | Pro |
| -0.86 | 1.08 | 1.92 | 10 | | 2 | Pro |
| 0.74 | 0.00 | 1.14 | 10 | | 2 | Pro |
| -0.83 | 1.96 | 1.90 | 10 | | 2 | Pro |
| -0.15 | 1.72 | 1.57 | 10 | | 2 | Pro |
| 0.04 | 1.88 | 1.48 | 10 | | 2 | Pro |
| -0.38 | 2.79 | 1.68 | 10 | | 2 | Pro |
| -0.16 | 1.32 | 1.57 | 10 | | 2 | Pro |
| -0.81 | 2.55 | 1.89 | 10 | | 2 | Pro |
| 0.47 | 0.00 | 1.27 | 10 | | 2 | Pro |
| 0.28 | 0.48 | 1.36 | 10 | | 2 | Pro |
| -0.14 | 1.97 | 1.57 | 10 | | 2 | Pro |
| 0.09 | 2.45 | 1.45 | 10 | | 2 | Pro |
| -0.94 | 2.09 | 1.96 | 10 | | 2 | Pro |
| -0.91 | 0.70 | 1.94 | 10 | | 2 | Pro |
| -0.24 | 2.27 | 1.62 | 10 | | 2 | Pro |
| -0.99 | 1.59 | 1.98 | 10 | | 2 | Pro |
| -1.18 | 1.08 | 2.07 | 10 | | 2 | Pro |
| -1.21 | 1.72 | 2.09 | 10 | | 2 | Pro |
| -0.42 | 2.63 | 1.70 | 10 | | 2 | Pro |
| -0.67 | 2.48 | 1.82 | 10 | | 2 | Pro |
| -0.39 | 2.57 | 1.69 | 10 | | 2 | Pro |
| 0.14 | 1.26 | 1.43 | 10 | | 2 | Pro |
| -0.12 | 2.23 | 1.56 | 10 | | 2 | Pro |
| 0.26 | 1.83 | 1.37 | 10 | | 2 | Pro |
| -0.70 | 1.20 | 1.84 | 10 | | 2 | Pro |
| -1.11 | 1.32 | 2.04 | 10 | | 2 | Pro |
| -0.11 | 2.51 | 1.55 | 10 | | 2 | Pro |
| -0.45 | 2.09 | 1.72 | 10 | | 2 | Pro |
| -0.49 | 2.31 | 1.73 | 10 | | 2 | Pro |
| -0.34 | 1.11 | 1.66 | 10 | | 2 | Pro |
| -0.33 | 1.18 | 1.66 | 10 | | 2 | Pro |
| 0.26 | 2.25 | 1.37 | 10 | | 2 | Pro |
| 0.03 | 2.38 | 1.48 | 10 | | 2 | Pro |
| -0.80 | 2.37 | 1.89 | 10 | | 2 | Pro |
| -1.20 | 0.78 | 2.08 | 10 | | 2 | Pro |
| -0.34 | 0.85 | 1.66 | 10 | | 2 | Pro |
| -0.49 | 2.67 | 1.74 | 10 | | 2 | Pro |
| 0.26 | 1.91 | 1.37 | 10 | | 2 | Pro |
| 0.09 | 1.49 | 1.45 | 10 | | 2 | Pro |
| 1.14 | 0.00 | 0.94 | 10 | | 2 | Pro |
| -0.84 | 0.95 | 1.91 | 10 | | 2 | Pro |
| -0.89 | 2.85 | 1.93 | 10 | | 2 | Pro |
| 0.32 | 0.30 | 1.83 | 13 | | 2 | Pro |
| -1.00 | 1.32 | 2.01 | 13 | | 2 | Pro |
| 0.39 | 2.69 | 1.82 | 13 | | 2 | Pro |
| -1.07 | 1.62 | 2.02 | 13 | | 2 | Pro |
| -0.47 | 1.86 | 1.94 | 13 | | 2 | Pro |
| 0.20 | 2.19 | 1.85 | 13 | | 2 | Pro |
| -0.89 | 2.76 | 1.99 | 13 | | 2 | Pro |
| -0.73 | 2.56 | 1.97 | 13 | | 2 | Pro |
| -0.70 | 2.11 | 1.97 | 13 | | 2 | Pro |
| -0.82 | 1.76 | 1.98 | 13 | | 2 | Pro |
| -0.38 | 2.03 | 1.93 | 13 | | 2 | Pro |
| -0.74 | 1.58 | 1.97 | 13 | | 2 | Pro |
| -0.33 | 1.89 | 1.92 | 13 | | 2 | Pro |
| -1.05 | 2.30 | 2.01 | 13 | | 2 | Pro |
| -0.55 | 1.66 | 1.95 | 13 | | 2 | Pro |
| -0.61 | 2.58 | 1.96 | 13 | | 2 | Pro |
| -0.50 | 1.90 | 1.94 | 13 | | 2 | Pro |
| -0.42 | 1.38 | 1.93 | 13 | | 2 | Pro |
| 0.40 | 0.78 | 1.82 | 13 | | 2 | Pro |
| 0.26 | 1.28 | 1.84 | 13 | | 2 | Pro |
| -0.25 | 2.55 | 1.91 | 13 | | 2 | Pro |
| -0.48 | 2.53 | 1.94 | 13 | | 2 | Pro |
| -0.04 | 2.15 | 1.88 | 13 | | 2 | Pro |
| -0.01 | 1.40 | 1.88 | 13 | | 2 | Pro |
| -0.59 | 2.35 | 1.95 | 13 | | 2 | Pro |
| 0.17 | 2.68 | 1.85 | 13 | | 2 | Pro |
| -0.68 | 1.76 | 1.97 | 13 | | 2 | Pro |
| -0.88 | 1.88 | 1.99 | 13 | | 2 | Pro |
| -0.15 | 2.77 | 1.89 | 13 | | 2 | Pro |
| -0.03 | 2.27 | 1.88 | 13 | | 2 | Pro |
| -0.80 | 1.43 | 1.98 | 13 | | 2 | Pro |
| -0.24 | 1.86 | 1.91 | 13 | | 2 | Pro |
| -0.74 | 1.80 | 1.97 | 13 | | 2 | Pro |
| -0.45 | 2.59 | 1.94 | 13 | | 2 | Pro |
| -0.80 | 1.52 | 1.98 | 13 | | 2 | Pro |
| 0.01 | 1.04 | 1.87 | 13 | | 2 | Pro |
| -0.74 | 2.30 | 1.97 | 13 | | 2 | Pro |
| 0.43 | 1.84 | 1.82 | 13 | | 2 | Pro |
| -0.16 | 2.93 | 1.90 | 13 | | 2 | Pro |
| -0.56 | 1.96 | 1.95 | 13 | | 2 | Pro |
| -1.21 | 1.91 | 2.03 | 13 | | 2 | Pro |
| -0.66 | 1.77 | 1.96 | 13 | | 2 | Pro |
| -0.64 | 1.93 | 1.96 | 13 | | 2 | Pro |
| 0.34 | 1.45 | 1.83 | 13 | | 2 | Pro |
| -0.30 | 1.43 | 1.91 | 13 | | 2 | Pro |
| -0.04 | 2.79 | 1.88 | 13 | | 2 | Pro |
| -0.25 | 2.16 | 1.91 | 13 | | 2 | Pro |
| 0.01 | 1.45 | 1.87 | 13 | | 2 | Pro |
| -0.84 | 1.18 | 1.99 | 13 | | 2 | Pro |
| -0.84 | 1.65 | 1.99 | 13 | | 2 | Pro |
| -0.74 | 2.52 | 1.97 | 13 | | 2 | Pro |
| -0.89 | 1.26 | 1.55 | Undisturbed | | 2 | Pro |
| -0.40 | 2.31 | 1.61 | Undisturbed | | 2 | Pro |
| -0.76 | 0.30 | 1.56 | Undisturbed | | 2 | Pro |
| -0.85 | 1.96 | 1.55 | Undisturbed | | 2 | Pro |
| -0.51 | 2.62 | 1.60 | Undisturbed | | 2 | Pro |
| -0.82 | 0.00 | 1.56 | Undisturbed | | 2 | Pro |
| -0.68 | 0.00 | 1.57 | Undisturbed | | 2 | Pro |
| -0.59 | 0.48 | 1.59 | Undisturbed | | 2 | Pro |
| -0.22 | 1.42 | 1.63 | Undisturbed | | 2 | Pro |
| -0.44 | 1.83 | 1.61 | Undisturbed | | 2 | Pro |
| -0.38 | 2.64 | 1.61 | Undisturbed | | 2 | Pro |
| -0.59 | 2.63 | 1.59 | Undisturbed | | 2 | Pro |
| -0.43 | 1.46 | 1.61 | Undisturbed | | 2 | Pro |
| -0.42 | 0.30 | 1.61 | Undisturbed | | 2 | Pro |
| -0.92 | 0.30 | 1.54 | Undisturbed | | 2 | Pro |
| -0.54 | 0.30 | 1.59 | Undisturbed | | 2 | Pro |
| -1.14 | 2.19 | 1.51 | Undisturbed | | 2 | Pro |
| -0.97 | 2.15 | 1.53 | Undisturbed | | 2 | Pro |
| -0.53 | 1.94 | 1.59 | Undisturbed | | 2 | Pro |
| -0.19 | 2.18 | 1.64 | Undisturbed | | 2 | Pro |
| -0.97 | 2.77 | 1.53 | Undisturbed | | 2 | Pro |
| -0.72 | 1.18 | 1.57 | Undisturbed | | 2 | Pro |
| -0.05 | 2.51 | 1.66 | Undisturbed | | 2 | Pro |
| -0.56 | 2.20 | 1.59 | Undisturbed | | 2 | Pro |
| -0.62 | 0.00 | 1.58 | Undisturbed | | 2 | Pro |
| -0.68 | 2.54 | 1.57 | Undisturbed | | 2 | Pro |
| -0.57 | 2.01 | 1.59 | Undisturbed | | 2 | Pro |
| 0.25 | 2.55 | 1.70 | Undisturbed | | 2 | Pro |
| -0.36 | 2.26 | 1.62 | Undisturbed | | 2 | Pro |
| 0.48 | 0.00 | 1.73 | Undisturbed | | 2 | Pro |
| -0.69 | 2.37 | 1.57 | Undisturbed | | 2 | Pro |
| -0.30 | 1.59 | 1.62 | Undisturbed | | 2 | Pro |
| -0.27 | 2.12 | 1.63 | Undisturbed | | 2 | Pro |
| 0.03 | 1.99 | 1.67 | Undisturbed | | 2 | Pro |
| -1.09 | 1.86 | 1.52 | Undisturbed | | 2 | Pro |
| -0.97 | 1.88 | 1.53 | Undisturbed | | 2 | Pro |
| -0.49 | 2.74 | 1.60 | Undisturbed | | 2 | Pro |
| -0.14 | 2.60 | 1.65 | Undisturbed | | 2 | Pro |
| -0.46 | 0.00 | 1.60 | Undisturbed | | 2 | Pro |
| 0.16 | 1.60 | 1.69 | Undisturbed | | 2 | Pro |
| -0.20 | 0.30 | 1.64 | Undisturbed | | 2 | Pro |
| -0.74 | 1.36 | 1.57 | Undisturbed | | 2 | Pro |
| -0.89 | 0.78 | 1.55 | Undisturbed | | 2 | Pro |
| -0.21 | 2.18 | 1.64 | Undisturbed | | 2 | Pro |
| -0.57 | 2.00 | 1.59 | Undisturbed | | 2 | Pro |
| -0.54 | 2.93 | 1.59 | Undisturbed | | 2 | Pro |
| -0.45 | 2.10 | 1.60 | Undisturbed | | 2 | Pro |
| -0.91 | 1.00 | 1.54 | Undisturbed | | 2 | Pro |
| -0.57 | 1.18 | 1.59 | Undisturbed | | 2 | Pro |
| -0.40 | 1.92 | 1.61 | Undisturbed | | 2 | Pro |
| -0.46 | 2.00 | 1.60 | Undisturbed | | 2 | Pro |
| -0.44 | 0.00 | 1.61 | Undisturbed | | 2 | Pro |
| -0.91 | 2.20 | 1.54 | Undisturbed | | 2 | Pro |
| -0.32 | 0.00 | 1.62 | Undisturbed | | 2 | Pro |
| -0.25 | 1.95 | 1.63 | Undisturbed | | 2 | Pro |
| -0.56 | 2.14 | 1.59 | Undisturbed | | 2 | Pro |
| -0.96 | 1.20 | 1.54 | Undisturbed | | 2 | Pro |
| -0.33 | 2.27 | 1.62 | Undisturbed | | 2 | Pro |
